# Advances in mucoadhesive drug delivery systems: Mechanistic insights, pharmacological performance, and translational pathways

**DOI:** 10.1016/j.crphar.2026.100261

**Published:** 2026-09-21

**Authors:** Farwa Zahra, Ahmed Raza Hashmi, Ram Narayanan Ravi, Mahendran Sekar, Ling Shing Wong, Ravindran Jaganathan, Rajeswari Ravindran

**Affiliations:** a Department of Pharmacy, Superior University Sargodha Campus, Sargodha, Punjab, Pakistan; b School of Pharmacy, Monash University Malaysia, Bandar Sunway, Subang Jaya, Selangor, Malaysia; c Jeffrey Cheah School of Medicine and Health Sciences, Monash University Malaysia, Bandar Sunway, Subang Jaya, Selangor, Malaysia; d Preclinical Department, Faculty of Medicine, Universiti Kuala Lumpur, Royal College of Medicine Perak, Ipoh, Perak, Malaysia; e Faculty of Pharmacy, University of Cyberjaya, Persiaran Bestari, Cyber 11, Cyberjaya, Selangor, 63000, Malaysia; f Faculty of Health and Life Sciences, INTI International University, Nilai, Malaysia

**Keywords:** Mucoadhesive drug delivery system, Pharmacological performance, Controlled drug release, Polymer–mucin interactions, Translational drug development

## Abstract

Mucoadhesive drug delivery systems (MDDS) have emerged as an important strategy in modern pharmacology to enhance drug residence time, improve mucosal absorption, and optimize therapeutic performance across diverse administration routes. By enabling intimate and prolonged interactions with mucosal tissues, MDDS directly influence pharmacokinetic and pharmacodynamic outcomes while supporting patient-centric, non-invasive drug delivery. Recent advances in polymer engineering, surface functionalization, and formulation design have further expanded the capability of MDDS to overcome biological barriers and address key limitations associated with conventional dosage forms. This review provides a comprehensive and mechanistically driven analysis of MDDS, focusing on the fundamental principles of mucoadhesion and the physicochemical and biological factors governing adhesive strength, mucus penetration, and controlled drug release. A wide range of mucoadhesive delivery platforms, including films, gels, tablets, patches, hydrogels, and particulate systems, are discussed in relation to their structure–property–function relationships and pharmacological performance across oral, nasal, ocular, gastrointestinal, vaginal, rectal, and pulmonary routes. The role of natural, semi-synthetic, and synthetic polymers, together with advanced functionalization strategies such as thiolation, ligand incorporation, stimuli-responsive networks, and nanotechnology integration, is critically evaluated with respect to enhancing adhesion, permeability, and therapeutic efficacy. Beyond formulation design, the review emphasizes translational and drug discovery-relevant considerations, including Quality by Design (QbD), manufacturing scalability, regulatory and safety expectations, and clinically evaluated and Food and Drug Administration-approved mucoadhesive products. Selected clinical trials and case studies of successful and unsuccessful formulations are examined to highlight key challenges in clinical translation and to identify emerging opportunities. By bridging mechanistic understanding with applied pharmacology and translational development, this review positions MDDS as a versatile and clinically viable platform for advancing next-generation drug delivery strategies and accelerating their progression from discovery to patient care.

## Highlights

• Mucoadhesive drug delivery systems enhance therapeutic performance by prolonging drug residence at mucosal interfaces.
• Mechanistic factors governing mucoadhesion, drug permeation, and controlled release across diverse mucosal routes are analyzed.
• Polymer selection and functionalization are pivotal to optimizing therapeutic efficacy and patient-centric outcomes.
• Clinically evaluated and FDA-approved mucoadhesive products highlight both translational success and development challenges.
• Regulatory, manufacturing, and quality-by-design considerations are essential for successful clinical translation.

## Graphical abstract

**Figure:**
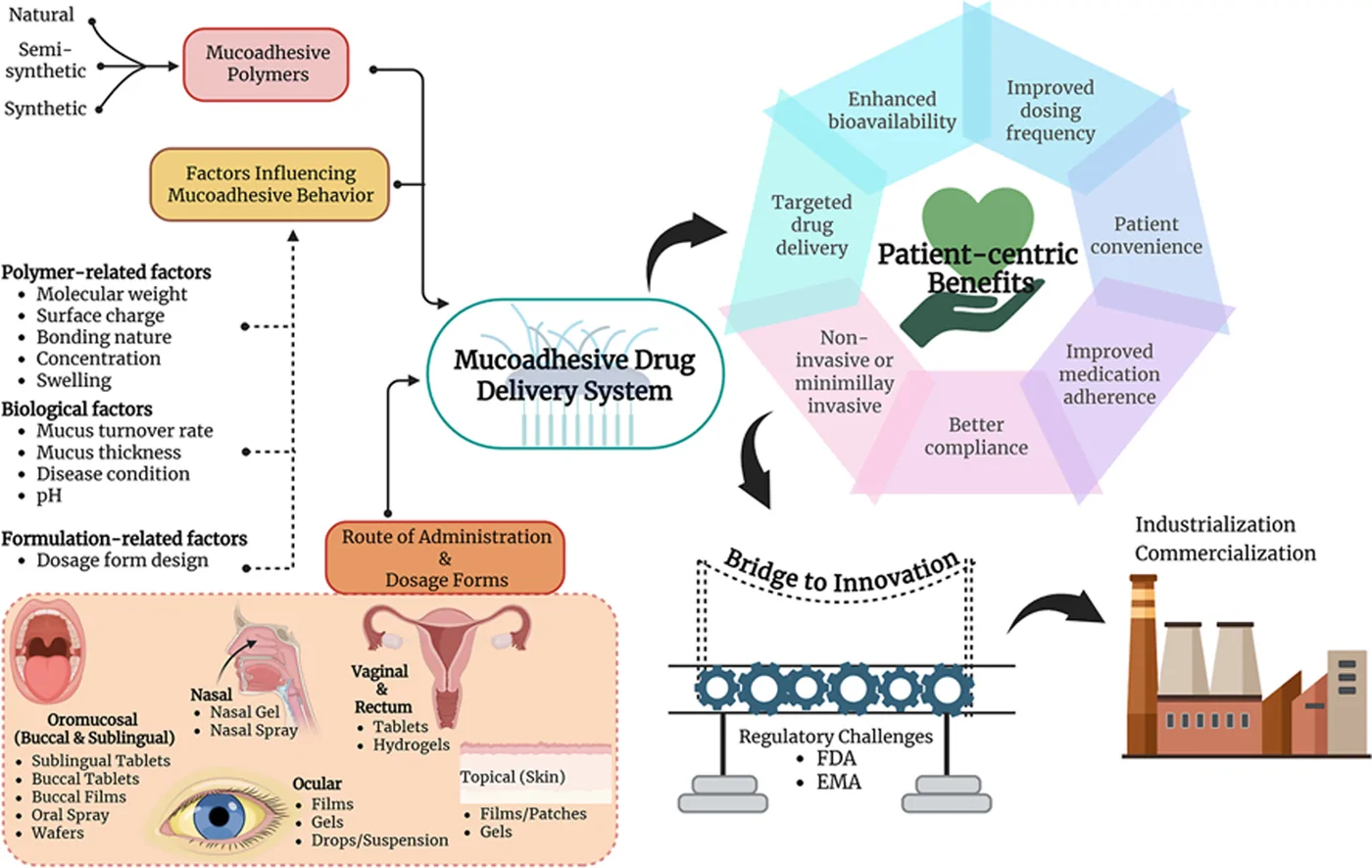

## 1 Introduction

In recent times, patient-centric drug design has achieved significance and is considered an additional performance indicator in pharmaceutical sciences (Menditto et al., 2020). Since then, to achieve clinical outcomes, there has been an increasing awareness of the need to place the patient as a primary stakeholder, raising the regulatory requirements for special populations across the globe (Stegemann et al., 2016; Grimsrud et al., 2015). Beyond the stability and pharmacokinetics, patient usability and acceptance for certain factors such as administration schedule, ease of use, tolerability, patient comfort, and taste masking effect should also be prioritized in the product development process, as they play an important role in long-term adherence, particularly in chronic therapies (Menditto et al., 2020; Religioni et al., 2025; Xiong et al., 2025). Poor adherence to medication is an emerging issue, strongly linked to negative therapeutic outcomes, increasing the disease burden, and higher patient costs (Religioni et al., 2025). Conventional dosage forms often come with limitations such as frequent dosing, short residence time, and patient discomfort; these factors contribute to poor adherence in real-world use (Karna et al., 2015).

Novel Drug Delivery Systems (DDS) are increasingly employed to improve the therapeutic outcomes (Sangavi et al., 2025). These strategies aim to mitigate the impact of mechanistic and physiological limitations, offering targeted delivery, controlled release, and flexible dosing regimens (Sharma et al., 2025). Among these, Mucoadhesive Drug Delivery System (MDDS) has gained recognition over conventional delivery systems (Pandey et al., 2023). MDDS enhance the local and systemic effects of drugs as they directly interact with the mucosal surface, provide site-specific targeting, increase residence time, and reduce dosing frequency, which leads to improved drug bioavailability, influencing patient compliance and therapeutic outcomes (Pandey et al., 2023).

Though many of the advanced drug delivery technologies, such as liposomes, implants, and nanoparticles (NPs), exist, MDDS uniquely blend simplicity, non-invasiveness, and compatibility with the mucosal milieu (Kulkarni et al., 2023; Hashmi et al., 2025). This review demonstrates that multiple mucosal routes, including oral, nasal, ocular, rectal, and vaginal, can be exploited for MDDS using diverse dosage forms such as films, gels, solutions, pastes, and patches. The availability of an increasing number of mucoadhesive products approved by the U.S Food and Drug Administration (FDA) shows their translational feasibility. However, even with this success, certain challenges are still present, such as interpatient variation in the mucosal environment, long-term stability and patient acceptance, manufacturing scalability issues, and regulatory challenges (Gagliardi et al., 2025).

This review synthesizes current knowledge on MDDS by first establishing the mechanistic basis of mucoadhesion and examining how physicochemical properties, biological variables, and formulation design collectively govern adhesive performance at mucosal interfaces. The discussion then extends to the spectrum of mucoadhesive platforms developed for different administration routes, highlighting how their design influences therapeutic applicability and practical limitations. Particular emphasis is placed on the selection and modification of natural, semi-synthetic, and synthetic polymers, with attention to functionalization approaches that modulate adhesion, permeability, and drug release kinetics. Beyond formulation science, the review situates MDDS within a broader translational and industrial context, addressing patient-centered considerations, clinically evaluated products, regulatory expectations, and Quality by Design (QbD) strategies that shape real-world development. By integrating insights from clinical trials, marketed formulations, and emerging research directions, this review provides a structured framework to guide the rational design and successful translation of MDDS.

## 2 Principles of mucoadhesive drug delivery systems

### 2.1 Classification

Mucoadhesion is a special type of bioadhesion that can be defined as the ability of a material to bind to a biological surface. For instance, the attachment of an epithelial tissue to a mucus coat is a perfect example of mucoadhesion (Haugstad et al., 2015). Mucoadhesion is also defined as an interaction that occurs between a mucoadhesive polymer and mucin, which is a glycoprotein on the mucous membrane (Leung et al., 1987). MDDS utilize the property of bioadhesive polymers, which get sticky upon hydration (Koirala et al., 2021). They are widely used for delivering drugs to specific target sites in the body and to the entire body. One of the main reasons for their versatility in terms of drug delivery is that they facilitate easy accessibility to the drugs while avoiding hepatic metabolism (first-pass effect). This enables increased safety and patient compliance (Alexander et al., 2011). They have shown excellent positive results in the treatment of various disease conditions, such as advanced oral malignancies (Bajoria et al., 2025), diabetes (Muñoz-Correa et al., 2024), cancer (Baskaran et al., 2024), glaucoma (Cheng et al., 2024), nasal infections (Mardikasari et al., 2024), glioblastoma (Ramalho et al., 2024), ulcerative colitis (Zhao et al., 2024), schizophrenia (Ahmad et al., 2024), pneumonia (Elnahas et al., 2024), and skin injury wounds (Sharma et al., 2024). They can be administered via different routes such as buccal (Viswanathan et al., 2025), sublingual (Sadat Hosseini et al., 2022), nasal (Peng et al., 2024), ocular (Shan et al., 2025), vaginal (Ci et al., 2017), and rectal (Sun et al., 2025). Based on the administration routes, they are available in different dosage forms, such as:

a) **Oromucosal:** Buccal tablets (Arpa et al., 2024), buccal film (Kurćubić et al., 2023), patches (Arpa et al., 2023), sublingual tablets (Ma'ali et al., 2023), wafers (Hanson et al., 2021), oral sprays (Tamane et al., 2023) (e.g., Belbuca® – FDA-approved buccal film for management of pain).
b) **Nasal:** Sprays (Pyrć et al., 2021), gels (Suhagiya et al., 2023), and NPs (Jamshidnejad-Tosaramandani et al., 2024) (e.g., Neffy® – FDA-approved nasal spray for type I allergic reactions, including anaphylaxis).
c) **Gastrointestinal (GI):** Matrix tablets (Yusif et al., 2016), NPs (Sunoqrot et al., 2017), GI patches (Lee et al., 2022) (e.g., Yaldigo®/Asacol 1600 – FDA-approved tablet with colon-targeting technology for ulcerative colitis).
d) **Vaginal and Rectal:** Hydrogel (Ramadan et al., 2018; Perinelli et al., 2018) (e.g., mucoadhesive gel containing propolis standardized extract for vulvovaginal candidiasis, which is currently in a Phase I clinical trial).
e) **Ocular and Pulmonary:** In-situ forming gels (Khattab et al., 2019), ocular drops (Liu et al., 2016), ocular films (Nanda et al., 2018), microspheres (Kapanigowda et al., 2015), and NPs (Popov et al., 2016; Taghe et al., 2019) (e.g., Refresh Liquigel® – FDA-approved in-situ forming gel for relieving dry and irritated eyes).

### 2.2 Mechanisms of mucoadhesion

The mechanism of mucoadhesion can be explained by numerous theories, such as wetting, adsorption, fracture, electronic, mechanical, and diffusion (Chatterjee et al., 2017).

Wetting theory (Jawadi et al., 2022) explains that the phenomenon of adhesion is the result of molecular interaction between two materials and surface forces. The critical parameter in this phenomenon is the spreadability, which determines the liquid's ability to wet the substrate surface. Adsorption theory states that the surface forces between molecules are responsible for the adhesion of a material to a particular surface. According to this theory, there will be two different chemical bonds created as a result of the surface forces (Ahuja et al., 1997). Adsorption can be interpreted by determining the amount of non-adherent particles or the amount of particles attached to the mucosal surface after making the polymer particles in contact with the surface of the mucosa (Ponchel et al., 1998).

The electronic theory follows the principle, as the name indicates, that there are different electronic structures for the mucin and mucoadhesive polymer, which results in the creation of an electric charge with a double layer at the interface. In this double layer, adhesion takes place because of attraction (Derjaguin et al., 1994). Fracture theory (Łysik et al., 2025) is based on the separation of an adhesive material from the surface of mucus with the help of mechanical force. This concept is generally beneficial in determining the strength of mucoadhesive bonds and detachment forces only. This theory does not cover the vital principle that is highly relevant for gel-based systems, which is the interpenetration that occurs between mucin and polymers at the molecular level.

The mechanical theory states that surface roughness is a critical parameter in deciding the adhesion of liquids to mucous surfaces by an interlocking mechanism. The interfacial area for adhesive interaction can also be increased by filling the irregularities of the rough surface (Lee et al., 2000). The last and final theory is the diffusion theory, which is based on the principle of macromolecular chain interpenetration and diffusion. The process of diffusion takes place between the polymeric matrix and mucus because of the concentration gradient at the interface, which is the driving force during the interaction of a mucoadhesive polymer and the mucosal surface. Hence, the rate of diffusion is completely dependent upon the concentration gradient and diffusion coefficient (Voyutskii, 1963).

### 2.3 Factors influencing mucoadhesion

#### 2.3.1 Physicochemical factors

The physicochemical factors affecting mucoadhesion can be categorized based on polymer-related properties such as molecular weight (MW) (Mortazavi et al., 1994), concentration (Madsen et al., 1998), surface charge (Jackson et al., 2000), hydrogen bonding (Patel et al., 2003), and swelling (Park et al., 1987).

MW is one of the critical parameters to achieve mucoadhesion, but its influence is unclear from a mechanistic point of view (Lai et al., 2009). Jeffrey et al. (Watchorn et al., 2022) examined the influence of MW on mucoadhesion with various ranges of mucoadhesive polymers. It was found in this study that as the MW of the polymers increases, there is a reduction in the affinity of mucoadhesive interactions. This clearly suggests that each polymer has an optimal MW for mucoadhesion. However, the trend was not completely clear, as there was a contrasting behavior after modifying the MW of each polymer. For example, there was no change in the molecular interaction after modifying the MW of polyvinylpyrrolidone (PVP). This highlights the influence of polymer mobility, which is independent of the MW, on its mucoadhesive behavior. The next factor is concentration; the concentration of the polymer is one of the essential factors in creating a strong adhesive bond (Bremecker, 1983). Burgalassi et al. (2015) investigated the effect of concentration on mucoadhesion. It was found that the concentration of polymer is significant due to its impact on the viscosity, which influences the formulation. Shin et al. (2000) reported that there was a significant increase in the viscosity and mucoadhesiveness when there was an increase in the concentration of poly(acrylic acid) (PAA) (up to 2%). However, above this limit, there was no change in bioadhesiveness. Understanding this relationship between concentration and mucoadhesiveness enables the fabrication of a desirable MDDS.

The third factor is surface charge; surface charge is very important for gastric retention and for mucoadhesion due to electrostatic forces. Jackson et al. (2000) evaluated the role of resin surface charge on gastric mucoadhesion. It was found that the resin, which is oppositely charged when compared to cholestyramine, does not possess the same properties, and the polymer coating reduced the effects; the surface charge played a critical role in terms of mucoadhesion and gastro-retention. In terms of secondary chemical interactions for mucoadhesion to take place, hydrogen bonding is one of the most important factors. Park et al. (1987) reported that, in order to have the ability to form hydrogen bonds, polymers should have ideal functional groups, which can ultimately result in effective mucoadhesion. Hence, polymer flexibility is very important in enhancing the hydrogen bonding potential. The last physicochemical factor affecting mucoadhesion is swelling; mucoadhesion on the gastric mucosa is dependent upon the adequate swelling and wetting of the mucoadhesive polymers. Agarwal et al. (2015) determined the physicochemical properties of mucoadhesive gastroretentive tablets. It was found that batches with a slower rate of swelling exhibited better mucoadhesion in comparison to batches with a higher rate of swelling. This study highlights the importance of swelling rate for mucoadhesion. All the above-mentioned factors must be carefully considered in designing an ideal MDDS.

#### 2.3.2 Biological factors

The different types of biological factors that influence mucoadhesion include mucin turnover (Rubinstein et al., 1994), pH (Pham et al., 2021), disease state (Jiménez-castellanos et al., 1993), and mucus thickness (Varum et al., 2010). Mucin is a component of the mucus that is generated in the stomach and shed throughout the entire gastrointestinal tract (GIT). The rate of mucus turnover is highly dependent on GI digestion activity, and the rate of mucus turnover is higher in areas with increased levels of gastric motility and proteolytic activity (Rubinstein et al., 1994). Mucoadhesion is a complex phenomenon in which mucus turnover plays a critical role. Mucus turnover is an inhibitory factor that resists mucoadhesion, and hence, for mucoadhesion to take place, the attraction between the mucus layer and the material should be strong enough to overcome this resisting force (Durrer et al., 1994). Tirosh et al. (1998) investigated the migration of adhesive and non-adhesive particles under modified mucus secretion conditions in the rat intestine. It was found that changes in mucus turnover have a significant influence on particle adherence, especially in the rat colon, which possesses relatively low mucus content. The second biological factor is pH; the change in pH will have a distinct impact on mucoadhesive polymers based on their properties. Nogueira et al. (2013) evaluated the influence of the gastric environment on the adhesion of *Helicobacter pylori (H. pylori)* to chitosan (CS) (mucoadhesive polymer). It was reported in this study that CS exhibited greater adhesion towards *H. pylori* at a low pH of 2.6. The morphology of *H. pylori* during adhesion is rod-shaped. However, pepsin reduced the adhesion of CS to *H. pylori* by changing the shape to coccoid at a higher pH of 6.0. This study highlights that the adhesion of mucoadhesive polymers is facilitated by the gastric condition, but pepsin could be an inhibiting factor. The distinct feature of the GIT with wide pH gradient ranging from highly acidic in the stomach (pH 1 to 2) to neutral or mildly alkaline in the intestine (pH 6 to 7.4). Along the GIT, the pH varies between 1.0 and 8.0, starting from the oral cavity to the colon (Celli et al., 2007). These pH variations greatly influence the structural, rheological, and conformational modifications in mucus due to the changes in the electrostatic charges of mucin glycosylated regions, exposure of hydrophobic domains of mucin, and non-covalent mucin-mucin interactions (Leal et al., 2017). Mucin gelation is induced by the changes in the physical properties of the main gel-forming mucins (mucin 2 (MUC2) in the intestine and mucin 5AC in the stomach) in acidic conditions (Schömig et al., 2016). The adhesiveness is significantly affected by the pH-dependent variations in viscosity (Perioli et al., 2013). Various *ex vivo* studies have reported that weak adhesion occurs at approximately pH 7.0 (neutral pH), and in contrast, strong adhesion at approximately pH 2.0 (low pH), highlighting the important role in the optimum functioning of mucus under fluctuations in the environmental conditions (Sumarokova et al., 2018). The high concentration of sulphate sugar residues (pKa < 1.0) and sialic acid (pKa ∼2.6) influences the net-negative charge of mucus at physiological pH due to their pKa (Badran et al., 2018). Hence, the isoelectric point of mucus is found within the pH range of 2.0 to 3.0, as the mucin presents a charge shift. Its conformational structure changes as the pH decreases (Caicedo et al., 2015) from a viscoelastic polymer solution (neutral pH) to a “weak gel” behavior (highly acidic pH) (Khutoryanskiy, 2011). At neutral physiological pH, mucin generally adopts an isotropic, randomly coiled conformation of its glycosylated chains (Georgiades et al., 2014), facilitated by electrostatic interactions involving polysaccharide side chains and disulfide linkages between cysteine-rich terminal regions. These interactions promote interchain entanglement, thereby increasing mucin viscosity (Abodinar et al., 2016). In contrast, under acidic conditions, protonation of carboxylate groups involved in salt bridges can alter intramolecular interactions and promote a more extended, rod-like conformation. Such pH-dependent conformational changes may consequently influence the accessibility and interaction of mucin chains with mucoadhesive polymers, potentially altering the strength and stability of polymer-mucin association (Olechno et al., 2025). Computational approaches, particularly molecular dynamics (MD) simulations, offer a promising strategy for investigating these interactions at the molecular level. For instance, MD simulations have been employed to examine the interactions of MUC2 with CS and thiolated CS derivatives and rank them based on the free energy of receptor-ligand interaction (Cavalcanti da Silveira et al., 2020). It was reported that thiolated CS derivatives exhibited different binding affinities toward a modeled MUC2 domain, with CS-Thiobutylamidine Conjugate (TBA-CS) and N-acetylcysteine (NAC)-CS showing the most favorable binding free energies (−273.0 and −269.6 kJ/mol, respectively), followed by unmodified CS (−211.2 kJ/mol). The stronger interaction of TBA-CS was primarily attributed to electrostatic interactions involving its protonated amidine group and the MUC2 residues aspartate 157 and glutamic acid 274, along with a greater number of hydrogen bonds. These findings demonstrate the potential of MD-based approaches to elucidate how polymer functionalization influences mucin–polymer association at the molecular level. Although the study did not explicitly examine different GI pH conditions, its MD/MM-PBSA analysis demonstrates the feasibility of resolving the molecular determinants of MUC2–polymer association, suggesting that future simulations incorporating pH-dependent protonation states could provide further insight into how the changing GI environment modulates these interactions.

The next factor is disease state; both topical and systemic diseases influence mucoadhesion. The disease state can indirectly affect mucoadhesion by affecting the rate of mucus turnover (Lehr et al., 1991). The properties of mucus change during disease conditions such as ulcerative colitis, gastric ulcers, cystic fibrosis, and other inflammatory conditions in the GIT. However, there is no clear understanding of the exact changes in the structure of the mucus. Hence, designing an MDDS under these conditions should involve preliminary evaluation of the mucoadhesive property in these conditions. The last biological factor is mucus thickness; the thickness of the mucus layer in the GIT ranges from 10 to 750 μm in humans (Gustafsson et al., 2012) and 100 to 800 μm in rats (Atuma et al., 2001). In humans, the small intestine comprises the thinnest mucus layer in comparison to the colon. Elderman et al. (2017) reported that old mice have reduced mucus thickness in comparison to young mice. Hence, understanding the changes in mucus with aging in humans will be very helpful in designing MDDS for geriatric populations, as there is no complete understanding yet.

#### 2.3.3 Formulation-specific factors

The formulation-specific factors that impede mucoadhesion include dosage form design (Akhmetova et al., 2025) and particle size (Abd El-Hameed et al., 1997). The selection of an appropriate dosage form is a critical step in designing an MDDS, where the selection is based on the route of administration, the need for controlled release, and the requirement of targeted action. The different categories of mucoadhesive dosage forms in the market include buccal tablets, buccal films, lozenges, oral solutions, oral patches, sublingual tablets, oral gels, etc. Each dosage form has unique properties and applications for various diseases. The most widely used dosage forms are tablets and films owing to their advantages of ease of administration and high patient compliance (Iacovelli et al., 2021; Protopapa et al., 2025). The particle size is a critical factor in diffusion across mucus, as it obstructs the diffusion of particles of any size. Hence, the particle size is more related to mucopenetration rather than mucoadhesion. As the particle size increases, the transport rate across mucus decreases. So, NPs made of polystyrene with a size of 500 nm have difficulty in penetrating the mucus in comparison to the NPs with a size of 100 nm (Yildiz et al., 2015; Lamson et al., 2020). However, the adhesion and penetration of the NPs can be enhanced by the process of surface functionalization. For example, among the NPs with a size of 500 nm, those functionalized with sulphate were able to penetrate nearly 1.7 times more than those functionalized with amino acids (Bandi et al., 2020). This highlights the relationship between particle size and surface functionalization, which should be considered in designing an ideal MDDS.

#### 2.3.4 Overview of mucoadhesion assessment methods

In the development of MDDS, there is a pressing need for reliable methods to evaluate mucoadhesion and to determine the *in vivo* interaction between the delivery system and the mucosa. This is an important step in determining the prospective use of formulations for clinical use. The methods can be classified into *in vitro*, *in vivo,* and *ex vivo*. *In vivo* and *ex vivo* are accurate methods, but for ethical reasons their use is often limited (Bassi da Silva et al., 2017; Djekic et al., 2020). The *in vitro* methods are convenient, avoid the use of animals, and enable reliable prediction of the *in vivo* mucoadhesive potential of MDDS. However, one of the limitations is that they do not completely reflect the *in vivo* processes (Khutoryanskiy, 2011).

Different types of *in vitro* mucoadhesion tests are:

(a) Tensile Strength: This method is based on the measurement of the force required to break the adhesive bond between mucosal tissue and polymer (Abu-Huwaij et al., 2011). Solid (Pund et al., 2011) and semi-solid dosage forms (De Souza Ferreira et al., 2017) can be evaluated using this method. Samprasit et al. (2026) evaluated the tensile strength of functionalized CS-based mucoadhesive buccal films loaded with triamcinolone acetonide. The films demonstrated tensile strengths of 6.7 to 15.3 N/mm^2^ that are suitable for oral applications. However, tensile strength decreased as CS composition increased. This reduction is due to the decline in film rigidity and flexibility, as CS's structure interferes with and weakens the poly(vinyl alcohol) (PVA) network.
(b) Spectrophotometric method: There are different kinds of methods utilizing spectrophotometry to evaluate the mucoadhesion interaction between the polymers and mucin (Kulkarni et al., 2016; Rossi et al., 2000; Fernandes et al., 2013). These techniques can be categorized into turbidimetric and calorimetric studies (Patil et al., 2010; Guerini et al., 2022). Turbidimetry is based on measuring the mixture's turbidity by assessing the formation of an interaction product between mucin and the polymer. Generally, absorbance at 500-650 nm is measured in accordance with the European Pharmacopoeia. A plot of turbidity vs mucin concentration enables the determination of the adhesive interaction potential (Caramella et al., 2015; Takeuchi et al., 2005). Isothermal Titration Calorimetry (ITC) is a powerful technique used for the study of thermodynamics of specific interactions between compounds in solution (Zhang et al., 2018; Filippov et al., 2018). This is the only technique that can provide various parameters, including free energy (binding constant-K_a_), enthalpy (ΔH), entropy (ΔS) and stoichiometry, in a single experiment. It directly measures the heat released or absorbed during the thermodynamic binding between a polymer and mucin (Yermak et al., 2022). This technique has been reported in studies of mucoadhesive interactions between mucins and polycarboxylic acids (Albarkah et al., 2015), epigallocatechin (Zhao et al., 2012), and CS (Meng-Lund et al., 2014). Yuan et al. (2026) reported the formulation of a mucoadhesive hydrogel for colon-specific delivery of *Lacticaseibacillus rhamnosus GG* comprising sulfhydryl-modified low-methoxyl pectin (PEC-SH) and CS-oligosaccharide (COS). ITC was employed to determine the interaction of intestinal mucin with PEC, PEC-SH, and composite gel particles. The ITC showed the strongest binding affinity of the PEC-SH/COS composite gel with mucin, indicated by the highest binding constant of 37.07 × 10^−6^ M^−1^. Sogias et al. (2008) reported the factors that contribute to the mucoadhesive properties of CS. The mucin/polymer interaction was indicated by increased turbidity due to aggregation of mucin particles with CS adsorbed on their surfaces. An excess of polymer causes a gradual decrease in turbidity due to particle disaggregation. In addition, hydrogen bonding and hydrophobic effects play important roles (Menchicchi et al., 2014). Contrary to polycationic CS, polycarboxylic acids with potential hydrogen donor and acceptor functional groups primarily form hydrogen bonds with mucin, as proven by turbidimetric titration at pH = 3 (Albarkah et al., 2015). Furthermore, turbidimetric and ITC data showed that the strength of the interactions depends on the polymer's MW.
(c) Surface Plasmon Resonance (SPR): Different kinds of techniques, including SPR, were developed to determine the molecular interactions in real time. SPR operates on the principle of quantifying interactions by analyzing variations in the refractive index induced by the binding of analytes to immobilized ligands (Topor et al., 2023). It is also one of the widely used techniques for obtaining quantitative insights into the behavior of the mucoadhesive polymer. Bravo-Osuna et al. (2012) evaluated the potential of SPR in determining the interaction between various polymers and transmembrane ocular mucins. It was reported that only the positively charged polymer, CS, showed optimal interaction with mucin, thereby indicating its mucoadhesive nature. The negatively charged polymers, i.e., hyaluronic acid and carboxymethyl cellulose (CMC), and the neutral polymers, i.e., hydroxypropyl methylcellulose (HPMC) did not show any signs of mucoadhesion. SPR-based biosensors provide highly efficient, real-time, and label-free analysis compared to conventional methods (Vachali et al., 2015). The limitations of traditional SPR include the increased time required for developing a mucin-coated SPR chip (Bravo-Osuna et al., 2012) and capturing data using a single polarization mode, namely p-polarization. However, the limitations can be overcome using plasmon-waveguide resonance, which operates on dual polarization modes, namely p- and s-polarization (Zhang et al., 2009).

## 3 Types of mucoadhesive delivery platforms

The MDDS can be classified based on the route of administration, such as oromucosal, nasal, GI, vaginal, rectal, ocular, and pulmonary, as mentioned in Fig. 1.

**Fig. 1:**
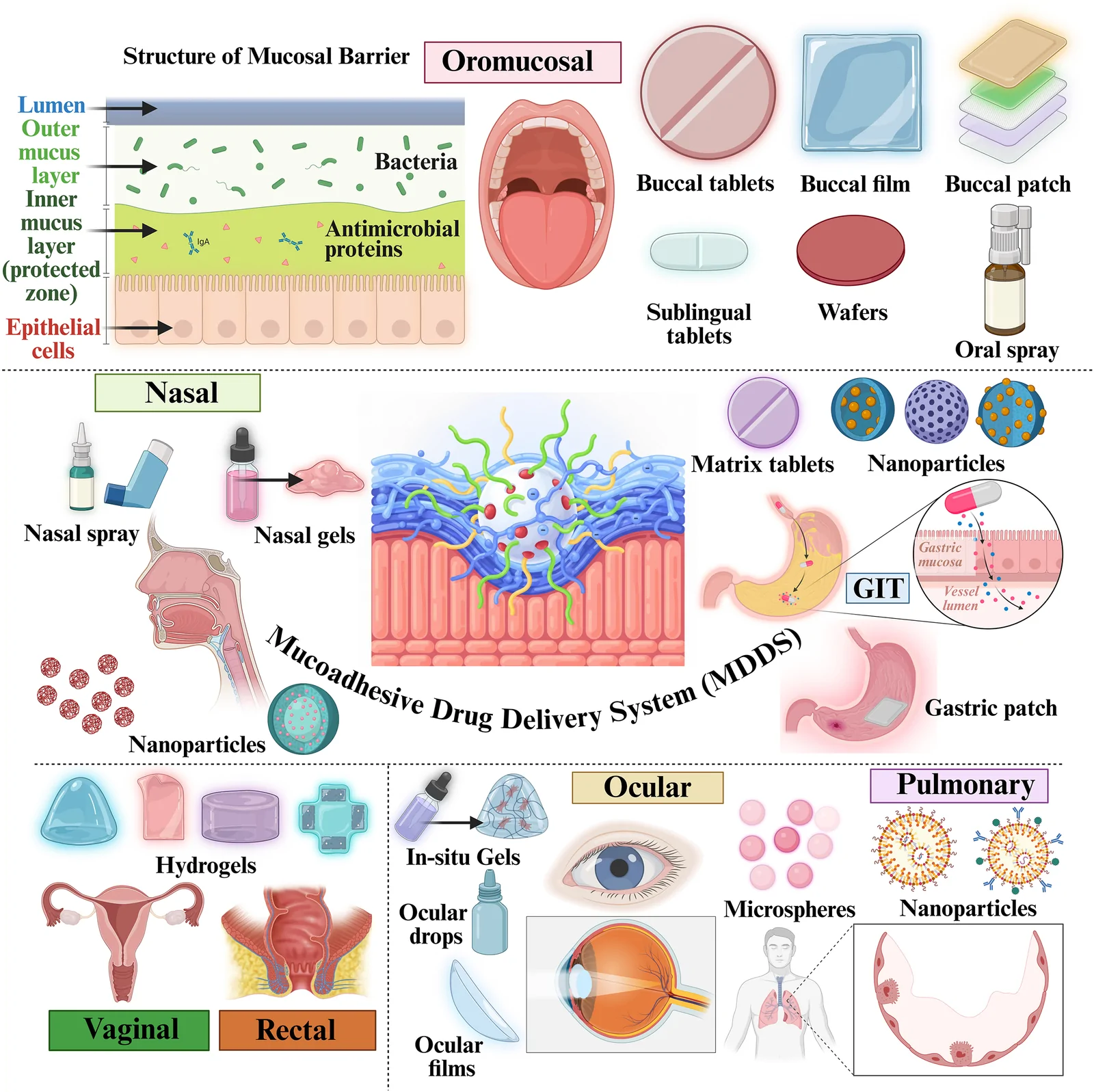
Mucoadhesive drug delivery platforms categorized by route of administration, showcasing associated dosage forms for drug delivery.

### 3.1 Oromucosal formulations (Buccal and Sublingual)

MDDS is an innovative delivery system that facilitates retention of various drugs for extended time periods at oral absorption sites such as buccal and sublingual. Compared to traditional delivery of drugs through the oral route, buccal and sublingual routes allow the absorption of drugs directly into the bloodstream by circumventing the first-pass metabolism and digestive tract (Laoasoke et al., 2024). The different kinds of technologies currently available include buccal tablets (Liu et al., 2025a), buccal film (Cheng et al., 2025), patches (Panahandeh et al., 2025), sublingual tablets (Park et al., 2025), wafers (Chettupalli et al., 2025), and oral sprays (Liu et al., 2025b). Arpa et al. (2024) designed a bilayer mucoadhesive buccal tablet (MBT) loaded with tizanidine hydrochloride for the treatment of pain-related spasms, multiple sclerosis, and stroke-related spasticity. The mucoadhesive polymers used in the study were CS glutamate, CS chloride, and ethyl cellulose. The developed tablets showed excellent swelling and mucoadhesion behavior. The entire drug was released in 8 h. *In vivo* studies in rabbits showed that the bioavailability was three times higher when compared to the Sirdalud® oral tablet (commercially available tablet).

Sheeba et al. (Fr et al., 2025) developed a mucoadhesive buccal film loaded with glipizide for treating diabetes. The polymer utilized in this study was HPMC E15, along with the combination of natural proteins such as albumin, gelatin, and casein. The developed formulation exhibited a sustained release for nearly 8 h with a percentage release of 89.29%, following zero-order kinetics. Bebawy et al. (2024) fabricated a buccal patch loaded with lidocaine for treating tooth pain in pediatrics. The various polymers used include HPMC, PVA, sodium-CMC (Na-CMC), and CS. The developed formulation exhibited a sustained release for 24 h. Another important highlight of this study is the uniformity in drug content in comparison to the variability observed in oral gels that are commercially available. Hence, it does not require frequent re-administration, which reduces the adverse effects. Asar et al. (2025) developed a sublingual tablet loaded with avanafil for treating erectile dysfunction. 2-hydroxypropyl-β-cyclodextrin (HPβ-CD) was the polymer used. The developed tablets exhibited superior properties in terms of greater residence time (nearly 5.98-fold) and increased plasma exposure (72 h) in comparison to the commercial tablet (Spedra). Mehrjardi et al. (2024) fabricated a mucoadhesive wafer loaded with phenytoin for treating wounds. CS, alginate, and HPMC were used as polymers. The developed wafers exhibited extended release and excellent mucoadhesion behavior. *In vivo* studies revealed that the wafers promoted faster healing of wounds in comparison to the conventional phenytoin ointment.

Maslii et al. (2024) designed an oral spray comprising clove carbon dioxide extract and essential oils of lavender and grapefruit for treating infectious and inflammatory diseases of the oral cavity. The polymers used include hydroxyethyl cellulose (HEC), hydroxypropyl cellulose (HPC), Na-CMC, sodium-alginate (Na-alginate), xanthan, PVP, and PVA. The developed spray showed increased viscosity, cohesion, and adhesion. However, the spray quality was reduced, thereby resulting in a small contact area of the mucous membrane. But the increased viscosity allows the drug to be in contact with the site for extended periods of time by forming a protective film. These studies highlight the potential of various oromucosal systems to treat a wide range of diseases. Many oromucosal systems are available commercially for various applications. In addition to the commercially available dosage forms, many are in the clinical trial stage to validate the safety and efficacy before reaching patients, as demonstrated in Table 1.

**Table 1:** Different kinds of mucoadhesive drug delivery systems in various phases of the clinical trials.

Dosage Form/Trial No	Active Pharmaceutical Ingredient	Condition	Clinical Trial Phase/Status	Study Design	Sample Size	Primary and Secondary outcome measures
Mucoadhesive Film/NCT02075749	Triamcinolone Acetonide/Licorice	Radiotherapy-induced oral mucositis (OM)	Phase I/Completed	Double-blind, randomized prospective trial	60	Pain score; Mucositis grade
Mucoadhesive Film/NCT02453503	Triamcinolone Acetonide/Licorice	Oral Lichen Planus (OLP)	Phase II/Completed	Double-blind, randomized trial	60	Visual Analogue Scale (VAS)
Mucoadhesive Film/NCT05219747	*Acmella oleracea* extract	No specific condition (Healthy individuals)	Phase I/Not yet recruiting	Double-blind, randomized trial	72	Potential for skin allergy reaction by skin prick test; Potential for skin allergy reaction by patch skin test (2^nd^, 4^th^ and 7^th^ day); Duration of mucosal adhesion; Local anesthetic effect; Adverse effect; Participant's satisfaction
Mucoadhesive Paste/NCT01680042	Phenytoin	Post-oral biopsy	Phase I/Recruiting	Double-blind, randomized trial	40	Healing biopsy wound by VAS
Mucoadhesive Strips/NCT05574049	Tetrahydrocannabivarin	Healthy non-diabetic obese adults	Early Phase I /Completed	Quadruple-blind, randomized trial	56	Weight loss; Decreased abdominal girth; Systolic and diastolic blood pressure; Aspartate Aminotransferase changes; Alanine Transaminase changes
Mucoadhesive hydrogels/NCT04317183	Chamomile	Chemotherapy-induced OM	Phase II/Recruiting	Open-label, randomized trial	45	Changes in severity of OM at different time points; Pain and discomfort severity at different time points using a numeric rating scale
Mucoadhesive patch/NCT07100613	Clobetasol/Resveratrol	OLP; OLP Related Stress; Oral Mucosal Disease; Oral Pain	Phase II/Recruiting	Triple-blind, randomized trial	45	Change in Symptom Intensity (Pain/Burning); Change in Clinical Severity of Lesions; Change in Oral Health-Related Quality of Life (QoL); Compliance with Treatment; Epithelial Structural Improvement
Mucoadhesive oral wound rinse/NCT02015559	N/A	Estrogen Receptor-positive Breast Cancer; HER2-negative Breast Cancer; Oral Complications; Progesterone Receptor-positive Breast Cancer; Recurrent Breast Cancer; Stage IIIB Breast Cancer; Stage IIIC Breast Cancer; Stage IV Breast Cancer	Phase II/Completed	Open-label, randomized trial	61	Rate of grades 1-4 stomatitis assessed using Common Terminology Criteria for Adverse Events (CTCAE); Rate of grade 3/4 stomatitis.
Mucoadhesive thermosensitive hydrogel/NCT06135259	Erythropoietin/Triamcinolone acetonide	OLP	Phase III/Not yet recruiting	Triple-blind, randomized trial	18	Clinical Scoring; Visual Analogue Score; Imaging and measuring surface area of the oral lesion; Tumor necrosis factor Salivary level
Mucoadhesive tablets/NCT03612414	N/A	Xerostomia due to hyposecretion of salivary gland	Phase I/Completed	Triple-blind, randomized trial	60	Effect of formulation on salivation; Safety; Effect on dry mouth symptoms; Effect on the pH of saliva
Mucoadhesive gel/NCT03511261	Curcumin	Oral Submucous Fibrosis	Phase II/Recruiting	Single-blind, randomized trial	200	Burning sensation and pain; Interincisal distance, tongue protrusion, and cheek flexibility
Mucoadhesive suspension/NCT06124976	Gemcitabine	Urothelial Carcinoma of the Renal Pelvis and Ureter; Transitional Cell Cancer of the Renal Pelvis and Ureter	Phase II/Recruiting	Open-label, non-randomized trial	70	Complete response rate; Durable efficacy; Objective response rate; Event-free survival; Number of adverse events; Peak Plasma Concentration (C_max_); Pharmacokinetics (T_max_)
Mucoadhesive mouthwash/NCT03706781	Cetylpyridinium Chloride + Benzydamine Hydrochloride	No specific condition (Healthy individuals)	Phase I/Completed	Open-label, randomized trial	26	Change in Local Adverse Reactions; Systemic availability of benzydamine (free base) through C_max_; Systemic availability of benzydamine (free base) through AUC(0-t); Incidence of Adverse Events (AEs); Palatability
Buccal soluble film/NCT00696137	Fentanyl	Respiratory Depression	Phase III/Completed	Open-label, non-randomized trial	5	The Number of Deaths in the Long-Term Follow-up
Mucoadhesive Buccal strip/NCT06733519	Nalmefene	Pruritus; Primary Biliary Cholangitis	Phase III/Not yet recruiting	Triple-blind, randomized trial	40	Safety and tolerability of repeat doses of TH104; Difference in daily itch score between TH104 and placebo; Change in participants reported outcome for PBC-40 questionnaire; Change in participants reported outcome for 5-D itch questionnaire.
Mucoadhesive gel/NCT04649697	Rebamipide	Rheumatoid Arthritis	Phase III/Recruiting	Quadruple-blind, randomized trial	39	Change in the Clinical improvement of oral ulcers; Subjective degree of pain; size of oral ulcer; healing time of the ulcer.
Mucoadhesive film/NCT03615820	N/A	Oral Recurrent Aphthous Ulcer	Phase I/Completed	Open-label, randomized trial	24	Determination of the onset of ulcer size reduction; Duration of complete ulcer healing
Mucoadhesive gel/NCT06214273	Chamomile	Chemotherapy-induced OM	Phase II/Not yet recruiting	Double-blind, randomized trial	45	Discomfort and pain; OM severity; total healing time of oral ulcers
MBT/NCT00390780	Miconazole Lauriad	HIV Infection	Phase III/Completed	Quadruple-blind, randomized trial	578	Clinical cure (defined as a complete resolution of signs and symptoms) after 14 days of treatment at the test of cure visit (day 17-day 22) using Murray scoring scale; clinical success at test-of-cure visit (day 17-22) (using Murray scoring scale); partial response at test of cure visit (days 17-22) using Murray scoring scale; mycological cure at the test of cure visit (day 17-22); relapse at the late post-therapy visit (day 35-38); oral discomfort using VAS; general and local tolerability and oral Discomfort
Mucoadhesive patch/NCT01388023	N/A	Halitosis; Gingivitis	Phase II/Completed	Single-blind, randomized trial	60	Reduction of VSC measurements with halimeter; reduction of halitosis as measured by judge score
Mucoadhesive NPs spray/NCT07082621	NAC	OM	Phase I/Completed	Single-blind, randomized trial	40	Change in pain scores; change in OM severity; Serum gastrin 17; Change in patient QoL.
Oral mucoadhesive gel/NCT07100145	Coconut oil	OLP	Phase IV/Not yet recruiting	Triple-blind, randomized trial	30	Clinical score; salivary levels of tumor necrosis factor-α and Interleukin-10
Mucoadhesive paste/NCT06988527	Honey; Triamcinolone acetonide	Erosive OLP; Atrophic Lichen Planus	Phase III/Recruiting	Double-blind, randomized trial	56	Clinical Assessment of OLP lesions using OLP score; Pain assessment using VAS; Long-term satisfaction of patients with OLP treated with honey, recurrence rates, and QoL after one year of treatment
Mucoadhesive hydrogel/NCT02735317	Chlorhexidine	Nasopharyngeal Neoplasms; Stomatitis	Phase II/Not yet recruiting	Open-label, randomized trial	90	Incidence of grade ≥ 3 mucositis; OM Assessment Scale; OM Daily Questionnaire, mouth and throat soreness question 2 (Q2); World Health Organization score; Interruption time during the schedule of radiotherapy; Change in body weight from baseline.

### 3.2 Nasal formulations

Different kinds of drugs can be delivered via the intranasal route for systemic, topical, and central nervous system action. The main target compounds for this route include those with poor intestinal absorption, GI instability, and reduced bioavailability due to first-pass metabolism. This is one of the important strategies for bypassing the blood-brain barrier and delivering the drug directly to the brain (Abo El-Enin et al., 2022; Nguyen et al., 2022). The different kinds of mucoadhesive technologies available for the nasal route include sprays (Pyrć et al., 2021), gels (Suhagiya et al., 2023), and NPs (Jamshidnejad-Tosaramandani et al., 2024).

Zucca et al. (2025) developed a thermo-responsive nasal spray loaded with HPβ-CD and randomly methylated-β-CD for treating respiratory infections. The polymer utilized in this study was sangelose (HPMC stearoxy ether). The developed formulation exhibited a transition of solution to gel at 29 to 34°C. They form a protective gel barrier on the mucosa at 37°C. The formulation showed properties ideal for nasal administration, such as excellent sprayability and spreadability. *In vitro* evaluation also exhibited non-toxicity. The droplet size was found to be greater than 100 μm, which is essential to prevent droplet exhalation. It was also reported that inhaled particles are significantly trapped due to the uniform deposition of the nasal spray on the nasal conchae. Salamah et al. (2025) fabricated an in situ gelling nasal spray with the objective of brain-targeted delivery. The formulation was loaded with cilostazol for treating cerebrovascular diseases. The polymers used are Span® 60, Tween® 80, Phytagel®, CS, and poloxamer-407 (P407). The developed spray exhibited excellent viscosity and mucoadhesive behavior. In both the nasal cavity and brain, the in situ gel formation enhanced drug release and permeability.

Kolhe et al. (2025) designed a thermosensitive in situ nasal gel loaded with artemether for treating malaria. P407 was used as a polymer in this study. The developed gel exhibited a significant permeation of nearly 95% at 10 h. The formulation was stable for six months with minor variations. *In vitro* antimalarial studies demonstrated that this formulation is a promising alternative to conventional therapies, as its efficacy closely aligns with the standard treatments. Singh et al. (2025) fabricated a thermosensitive in situ nasal gel loaded with risperidone for treating psychosis. The developed formulation is a strategy for nose-to-brain targeting. P407 and HPMC were used as polymers in this study. The in situ gels exhibited significant gelling capacity and mucoadhesive behavior. The formulation demonstrated sustained release and followed first-order kinetics. *In vivo* studies in rats showed that the area under the curve (AUC) from zero to infinity (AUC_0–∞_) in the brain was nearly 5.4-fold higher compared to the conventional oral solution. Kalra et al. formulated NPs loaded with rivastigmine for treating Alzheimer's disease. Eudragit RL 100 was used as a mucoadhesive polymer in this study. The NPs exhibited a significant mucoadhesive strength of nearly 85%. In comparison to transdermal patches, the NPs demonstrated greater drug transport percentage and drug targeting efficiency. The release was found to be in a sustained release fashion. Finally, the concentration of the drug in the mouse brain was comparatively higher following intranasal administration in comparison to other routes.

### 3.3 GI formulations

MDDS can be used for targeted drug delivery in a sustained and controlled release manner to the GIT to treat loco-regional diseases such as *H. pylori* infection (Bachir et al., 2025), inflammatory bowel diseases (Lim et al., 2025), etc. The currently available mucoadhesive technologies targeting the GIT include matrix tablets (Yusif et al., 2016), NPs (Sunoqrot et al., 2017), and GI patches (Lee et al., 2022).

Nigusse et al. (2021) fabricated a matrix tablet loaded with ranitidine hydrochloride for treating erosive esophagitis. The polymers used in this study were HPMC K100M, microcrystalline cellulose, and Na-CMC. The formulated matrix tablet demonstrated good bioadhesive strength and a swelling index of 29.69 g and 315%, respectively. The drug release profile exhibited a sustained release pattern over 12 h, and approximately 94% of the drug was released. Khizer et al. (2023) designed 3D-printed matrix tablets loaded with gabapentin for treating the condition of overactive bladder. Polyethylene oxide was used as the polymer. The extrusion technique was used for the 3D printing of the tablets. The formulated tablets exhibited an excellent floating time of nearly 10 h. The mucoadhesion studies on porcine stomach skin showed that the formulation exhibited good mucoadhesive behavior. The drug release pattern followed a sustained release profile. *In vivo* studies on white albino rabbits revealed that the oral absorption of the tablets was significantly higher compared to the commercial oral solution.

Rehman et al. (2024) developed pH-sensitive NPs-loaded with doxorubicin for cancer management. CS and Na-alginate were used as polymers to impart mucoadhesion to the dosage form. The developed NPs demonstrated excellent mucoadhesion and swelling properties. The drug release profile was found to follow a sustained release pattern for 24 h, and nearly 68% of the drug was released at a particular pH (5.6). The formulation had a shelf life of >2 years, based on the accelerated stability studies. Jain et al. (2022) formulated NPs loaded with metformin for the management of diabetes (Type-2). Seed starch from jackfruit seeds was used as a polymer in this study. The formulated NPs demonstrated extended floating in the GIT for 24 h. The release pattern followed a sustained release fashion for 24 h, and nearly 94% of the drug was released. *In vivo* studies on streptozotocin-induced diabetic rats demonstrated that NPs exhibited significantly greater potential in reducing the blood glucose levels compared to the free drug. Bandi et al. (2023) reported the formulation of a mucoadhesive gastric patch loaded with Regorafenib for the management of cancer. The different kinds of polymers used in this study are Eudragit RS100, polycaprolactone, Carbopol 934, PVP K90, CS, polyethylene glycol (PEG) 400, polylactic acid, and polydimethylsiloxane. The mucoadhesion studies in rat gastric mucosa showed that the gastric patch had excellent mucoadhesive strength of nearly 18 kPa. The drug release pattern followed a sustained-release manner for nearly a month, followed by zero-order kinetics. *In vivo* studies in rats demonstrated that the patch was able to sustain the plasma concentration of the drug for almost 8 days, and there were no histological changes compared to normal gastric tissue. There was a significant reduction in tumor volume within 7 days after a single dose of the gastric patch.

### 3.4 Vaginal and rectal formulations

Vaginal (Akhmetova et al., 2025) and rectal (Al-Mahmood et al., 2025) routes are among the effective routes but are underexplored for drug administration, which provides both local and systemic therapeutic effects. One of the unique advantages of vaginal delivery is that the relatively large surface of the vaginal mucosa enables high blood flow (Sahoo et al., 2013). Similarly, rectal delivery of drugs promotes high absorption and bioavailability compared to the oral route (Kim et al., 1998; Choi et al., 1998). Among the widely reported mucoadhesive technologies for vaginal and rectal drug delivery, hydrogels (Ramadan et al., 2018; Perinelli et al., 2018) are the most prominent.

Permana et al. (2023) fabricated the thermoresponsive hydrogel loaded with metronidazole for treating bacterial vaginosis. The polymers used in the study were gellan gum, CS, Pluronic® F127, and Pluronic® F68. The developed hydrogel demonstrated a sustained release profile for nearly 24 h. The sol-gel transition occurred ideally at vaginal temperature. The formulation exhibited a significant residence time at the vaginal tissue for nearly 24 h, based on *ex vivo* studies. *In vivo* evaluation using rats showed a reduction in the viability (>95%) of bacterial species *E. coli* and *S. aureus*. Afloarea et al. (2024) designed a hydrogel loaded with progesterone for the management of premature birth. The polymers utilized were CS, hyaluronic acid, and N-isopropylacrylamide. The developed hydrogel exhibited significant swelling and bioadhesive properties. It was found that the increase in CS concentration enhanced the mucoadhesion. *In vitro* cytotoxicity studies revealed that the formulation does not induce any cytotoxicity. The release of the drug followed a controlled release pattern. Reis et al. (2022a) developed a nano-hydrogel loaded with diphenyl diselenide for the management of trichomoniasis. The polymers utilized include Eudragit® RS100, locust bean gum, and Tween 80®. The developed hydrogel exhibited excellent mucoadhesive behavior. The nanoformulation enhanced the free radical scavenging of the drug. *Ex vivo* permeation studies using cow vaginal mucosa demonstrated significant permeability of the formulation. Toxicological screening using Hen's Egg Test Chorioallantoic Membrane (HET-CAM) showed no lysis, coagulation, or hemorrhage, which indicates the non-irritating nature of the dosage form. Salatin et al. (2022) developed a rectal hydrogel loaded with sumatriptan for the management of migraine and cluster headaches. PF-127 and CS were used as polymers in this study. The formulation demonstrated a good mucoadhesive strength of 732.48 N/cm^2^. The transition of sol to gel took place ideally at 32°c, which is very suitable for rectal delivery. The hydrogel network significantly reduced the release rate of the drug compared to the free drug, thereby providing extended release. The formulation had a shelf life of 3 months, based on the accelerated stability studies. Sakran et al. (2022) fabricated a rectal ethosomal hydrogel loaded with domperidone for the prevention of emesis associated with chemo and radiotherapy. The polymers utilized in this study include lecithin, Carbopol 934, Tween 80, and Span 60. The developed hydrogel demonstrated high mucoadhesion towards rectal mucosa. The drug release profile was found to follow a sustained release pattern for 6 h, and nearly 76% of the drug was released. *Ex vivo* permeation studies using rat intestinal mucosa demonstrated the increased permeation (nearly 4 times) of the hydrogel compared to free suspension. *In vivo* studies in Wistar rats showed that the hydrogel increased the C_max_ and AUC_0–48_ (∼2-fold) compared to free gel.

### 3.5 Ocular and pulmonary formulations

Traditional ocular dosage forms like eye drops and salves have numerous limitations, such as poor bioavailability, short retention times, frequent reapplication, and reduced patient compliance (Han et al., 2023). These limitations can be addressed by the use of mucoadhesive delivery systems (Chughtai et al., 2025). In the case of the pulmonary route, the increased residence time of drug compounds at the pulmonary mucosa is essential for the uptake of drugs (Edsbäcker et al., 2006). This can be achieved with mucoadhesive dosage forms that contain mucoadhesive agents that increase the residence time (Sugiura et al., 2025). The currently available mucoadhesive technologies targeting ocular and pulmonary delivery include in-situ forming gels (Khattab et al., 2019), ocular drops (Liu et al., 2016), ocular films (Nanda et al., 2018), microspheres (Kapanigowda et al., 2015), and NPs (Popov et al., 2016; Taghe et al., 2019). Recent advances in ocular hydrogel-based systems have further emphasized the roles of mucoadhesion, controlled drug release, nanotechnology integration, stimuli responsiveness, and advanced fabrication approaches, while scalability, mechanical stability, and manufacturing costs remain important translational considerations (Sabbagh et al., 2025).

Esteruelas et al. (2022) developed an *in-situ* forming gel loaded with Riluzole for treating glaucoma. Poly (lactic-co-glycolic acid) (PLGA)-503H was used as the polymer. The formulated *in-situ* gel demonstrated excellent mucoadhesive strength of 1374 dyne/cm^2^ compared to simulated tear fluid. The viscosity of the developed gel was lower (nearly 100 times) compared to gels with high mucoadhesion. This interesting feature of high mucoadhesion and low viscosity allows efficient distribution across the ocular surface. The drug release pattern followed a controlled release manner for 24 h, and 75% of the drug was released. The release kinetics fitted into the Korsmeyer-Peppas model. The analysis of ocular irritation using the HET-CAM method showed the non-irritating nature of the dosage form. Similarly, *in vivo* ocular tolerability studies using albino rabbits also showed the non-irritating nature of the gels. *In vivo* ocular uptake studies in C57BL6 mice showed the distribution of the drug within 24 h in the posterior ocular segment. Szalai et al. (2022) fabricated mucoadhesive eye drops loaded with dexamethasone for treating ophthalmic inflammations. The polymers utilized in this study include 2-HPβ-CD, 2-hydroxypropyl-γ-cyclodextrin, (2,6-di-O-methyl)-β-cyclodextrin, sulfobutylether-β-cyclodextrin, Kolliphor® P407, and HPMC. The eye drops exhibited good mucoadhesive behavior. The permeability of the eye drops was significantly higher compared to the standard suspension. The release of the drug for the initial 2 h followed the same pattern as the suspension, then showed an extended release.

Gupta et al. used a male albino rabbit model for evaluating the influence of platform architecture on ocular residence. For this purpose, precorneal clearance and biodistribution dynamics were investigated by using gamma scintigraphy (Gupta et al., 2013). Researchers designed a novel formulation, PLGA NPs-laden CS in situ gel for ophthalmic delivery of sparfloxacin. The performance of the designed formulation was compared with a marketed sparfloxacin eye drop formulation, a sparfloxacin nanosuspension, and a CS in situ gel. Scintigraphic dynamic imaging over 30 min demonstrated that precorneal retention followed the sequence of marketed formulations < nanosuspension < in situ gel < NPs-laden in situ gel. Due to the cationic mucoadhesive nature of the CS matrix, which electrostatically binds to the anionic conjunctiva and limits the rapid clearance of the entrapped PLGA NPs, the NPs-laden in situ gel exhibited the slowest clearance rate and remained on the ocular surface for the longest duration. These findings confirm that combining colloidal NPs carriers with a mucoadhesive, pH-sensitive polymeric gel matrix successfully overcomes rapid nasolacrimal clearance, offering a superior synergistic approach to prolonging ocular drug residence (Gupta et al., 2013).

Abilova et al. (2025) designed an ocular film loaded with pilocarpine for the management of glaucoma. Gellan gum and poly(2-ethyl-2-oxazoline) are the polymers used in this study. The developed ocular film demonstrated significant mechanical strength and mucoadhesion. Both properties increased with an increase in the concentration of gellan gum. *In vivo* studies in rabbits showed a sustained effect up to 240 min in comparison to eye drops. It was also found that there were no side effects (excessive tear production, tissue redness, or inflammation of the eyes) in rabbits, making the developed formulation an ideal strategy for treating glaucoma. Amore et al. (2017) formulated a microparticle loaded with a corticosteroid for treating chronic obstructive pulmonary Disease. The polymers used are CS oligosaccharide lactate, Pluronic F-68, and Na-alginate. The Na-alginate-based microparticles exhibited better mucoadhesive properties compared to CS-based microparticles due to the increased amount of interaction between Na-alginate and mucin. The drug release studies showed the retainment of nearly 40% of the drug even after 2 days. Dong et al. (2020) developed mucoadhesive NPs loaded with baicalein for treating respiratory disorders. CS and Pluronic F127 were used as polymers in this study. The developed NPs demonstrated a sustained release of drugs for 4 h. The amount of drug release was nearly 23% for CS-based NPs and 31% for Pluronic F127-based NPs. *In vivo* studies using Kunming mice showed sparse (CS-based) and widespread distribution (Na-alginate-based) in the mouse airways. The lung distribution of both the NPs was significantly higher than that of the free drug.

Mucoadhesive delivery can be provided via films, gels, NPs, and hybrid systems; however, the biological and formulation requirements of the intended mucosal route should be considered for assessing their performance. Films and patches allow prolonged localized exposure and controlled release but could be limited by their flexibility, hydration capacity, and large-scale manufacturing (Nair et al., 2023; Jacob et al., 2021). On the other hand, hydrogels and in situ gels serve as three-dimensional drug reservoirs, which can be made to respond to stimuli in terms of gelation (Sabbagh et al., 2025). NPs allow better control over their size, surfaces, encapsulation and release, and permeability of the formulation (Zheng et al., 2025a). However, these systems are not necessarily interchangeable or universally superior. Their relative performance depends on several factors, such as targeted mucosa, mucoadhesion, mucopenetration, residence time, and clearance mechanisms (Allahbedashti et al., 2026).

*In vivo* evidence further supports the selection of MDDS platforms to maintain drug exposure under dynamic mucosal conditions. Mirzaeei et al. constructed erythromycin-loaded NPs-impregnated mucoadhesive ocular inserts. For *in vivo* evaluation, the designed inserts were placed into the rabbits’ cul-de-sac, followed by the collection of tear fluid via a non-invasive sampling technique. Inserts exhibited AUC_0-120_ of 30,000–36,000 μg h/mL and C_max_ > 1800 μg/mL at 4 h. Moreover, drug concentration >5 μg/mL for up to 120 h was maintained (Mirzaeei et al., 2022). Similarly, bilayer mucoadhesive films were prepared for buccal administration to enhance the bioavailability of tizanidine HCl and meloxicam. *In vivo* pharmacokinetic parameters like AUC, C_max_, T_max,_ and t_1/2_ were evaluated using albino rabbits. A bilayer tizanidine/meloxicam buccal film provided sustained tizanidine release and permeation over 8 h in rabbits, with an increased tizanidine half-life from 2.51 to 9.91 h and AUC_0–t_ from 149.1 to 1043.4 ng h/mL compared with oral solution (Zaman et al., 2018). Overall, these studies illustrate that residence time and a controlled-release system may result in significant drug release and effects from a pharmacokinetic or pharmacodynamic perspective in the *in vivo* situation. However, these results are route- and formulation-dependent, stressing that the *in vitro* release patterns must be confirmed by *in vivo* pharmacokinetic, pharmacodynamic, or tissue-retention studies to establish controlled release behavior under dynamic mucosal conditions.

## 4 Polymeric materials used in mucoadhesive systems

The contribution of polymers to mucoadhesive delivery systems development shouldn't be overlooked. The mucosal sites, i.e., buccal, sublingual, nasal, ocular, rectal, and vaginal, assist drug delivery by bypassing the first-pass hepatic metabolism and promote the drug's systemic circulation for prolonged therapeutic utility. Detailed awareness regarding the polymeric materials is mandatory in terms of their utilization for the fabrication of mucoadhesive systems (Surendranath et al., 2022). Mucoadhesive systems have an adequate potential for enhancing drugs' retention and facilitating sustained, controlled, and targeted drug delivery, which offers greater pharmacological activity because of polymer-induced mucoadhesion. Furthermore, for bioadhesion and mechanical strength, these delivery systems rely on a wide variety of natural, semi-synthetic, and synthetic polymers (Kumar et al., 2022). To strengthen mucosal interactions and tune various attributes like swelling, permeability, and degradation for controlling drug release kinetics, polymers are frequently modified to fulfill the challenging performance targets (Shatabayeva et al., 2024). At present, multiscale design and hybrid constructs significantly modify the utilization of mucoadhesive systems (Bayer, 2022). However, rigorous safety and regulatory evaluations are required for translating the mucoadhesive excipients into approved products (Vigani et al., 2023). Moreover, the current European Medicines Agency (EMA) guidelines highlight the implications of various aspects, mainly including the assessments of biocompatibility, potential hypersensitivity, impurity profiles, and proper labeling (Agency, 2018). Collectively, all these factors govern the translation potential of mucoadhesive systems.

### 4.1 Natural, semi-synthetic, and synthetic polymers

The MDDS usually adhere to the mucosal membranes to enhance the retention and absorption of drugs that rely on mucoadhesive polymers. These polymers are categorized on the basis of their origin, aqueous solubility, bonding nature, and type of charge (Kumar et al., 2020; Subramanian, 2021), as mentioned in Fig. 2. This division facilitates the selection of polymers while fabricating the MDDS. Moreover, on the basis of origin, the polymers are further classified into natural, semisynthetic, and synthetic polymers (Ugoeze, 2020). Examples of frequently employed mucoadhesive polymers are described in Table 2 to highlight their significance in the pharmaceutical sector. Additionally, studies were mentioned to emphasize the effects of mucoadhesive polymers on mucoadhesion, retention time, and drug release, along with the toxicity profile.

**Fig. 2:**
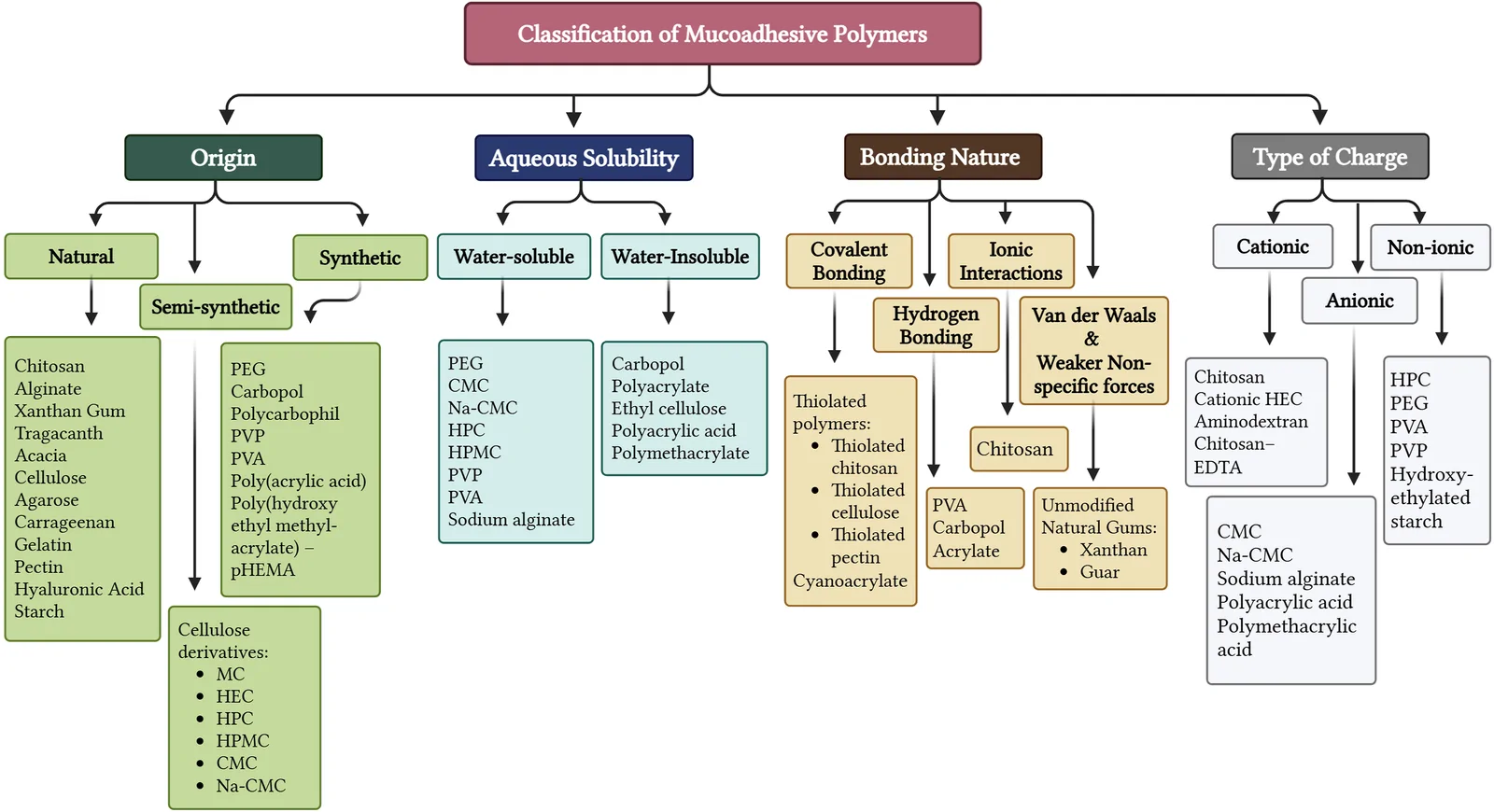
Schematic demonstration of mucoadhesive polymer categories on the basis of their origin, aqueous solubility, bonding nature and type of charge on their surfaces.

**Table 2:** Comparative mucoadhesive and drug-release performance of frequently employed mucoadhesive polymers in pharmaceutical delivery systems.

Class of Polymer	Mucoadhesive Polymer		Incorporated Drug	Mucoadhesive Dosage Form	Target site	Study model	Mucoadhesive Performance/Residence	Drug-Release Profile	References
Natural	CS		Insulin	NPs	Nasal mucosa → CNS	***In vitro:*** Simulated nasal fluid for evaluating drug release kinetics. ***Ex vivo:*** ovine nasal epithelium in a Franz diffusion cell.	Positive ζ potential (+21.6 mV) facilitated strong binding with negatively charged sialic acid residues in nasal mucus. NPs preserved mucosal integrity, as no epithelial damage was observed after 1 h of exposure, exhibiting excellent mucosal tolerability.	From CS-NPs, insulin release followed a biphasic release pattern; within the first 15 min, rapid burst drug release was observed. Followed by sustained release > 90% within 4 h.	Jamshidnejad-Tosaramandani et al. (2023)
			Latanoprost and Timolol	CS–GQD Nanocomposite suspension	Ocular surface → glaucoma management	***In vitro:*** Simulated tear fluid (pH 7.4). HEC cells were utilized for cytocompatibility evaluation. ***Ex vivo:*** HET-CAM was used for assessing ocular tolerance.	Protonated CS amine groups assisted to develop interaction with ocular mucosa sialic acid residues. After complex formation, increased hydrodynamic size (1584 nm) demonstrated strong mucin-binding evidence, leading to prolonged ocular residence.	The resulting formulation exhibited sustained drug release for up to 72 h.	Kumara et al. (2024)
	Alginate		Posaconazole	Alginate/pectin Buccal films	Oral mucosa, i.e., buccal cavity for antifungal activity	***In vitro:*** Simulated saliva solution. *Candida albicans, Candida krusei, and Candida parapsilosis* were incorporated in the study for evaluating the antifungal activity by using the plate diffusion technique. ***Ex vivo:*** Porcine buccal mucosa for investigating the mucoadhesion and residence time.	Alginate and pectin interacted with mucin via hydrogen bonding and electrostatic interaction. Mucoadhesive strength ranged from 0.276 ± 0.05 to 0.80 ± 0.06 N, with adhesion work ranging from 126.38 ± 28.57 to 218.04 ± 99.43 μJ. Residence time up to 205.00 ± 10.00 min was observed because of polymers cross-linking network.	Accelerated drug release in non-cross-linking films was observed because of pectin inclusion, as it increases the swelling ratio. In contrast, cross-linking restricts swelling and prolongs drug release. Release followed mainly zero-order kinetics (R^2^ = 0.79–0.99) and Fickian diffusion (n = 0.05–0.17).	Szekalska et al. (2023)
			Not drug-specific	Gel and powder of Na-alginate	Nasal cavity (rats)	***In vivo:*** Male Wistar rats (250-300 g)	*In vivo* nasal retention time ranged from 13.6 to 215.3 min. After 240 min, 48.0% and 58.7% of alginate powder and CMC-Na powder were retained in the nasal cavity.	Not reported	Inoue et al. (2025)
	Xanthan gum		Novel anti-Candida peptide	Thermoresponsive mucoadhesive hydrogel	Mucosal tissues and superficial skin *→ C. albicans* infections	***In vitro:*** Mouse embryonic fibroblast cell line for cytocompatibility and *C. albicans* for evaluating the antifungal activity. ***Ex vivo:*** Freshly obtained porcine skin tissue	XG-pNIPAM hydrogel demonstrated ∼5.4 N mucoadhesive force and ∼6.3 N for xanthan gum on mucin. Hydrogel remained attached to porcine tissue for 24 h *ex vivo.*	0.234% (w/v) AMP14d was loaded within 5% (w/v) XG-pNIPAM hydrogel. Within 24 h, 90% cumulative drug release was achieved, with ∼5% at 0.5 h, ∼21% at 1 h, ∼26% at 2 h, ∼46% at 4 h, ∼56% at 6 h, and ∼58% at 8 h.	Barberi et al. (2024)
			Nystatin (antifungal), Hydrocortisone acetate (anti-inflammatory), Lidocaine HCl (local anesthetic)	Buccal films	Buccal mucosa (oral cavity) → candidiasis treatment	***In vitro:*** Simulated saliva and *C. albicans* were incorporated for assessing antifungal activity. ***Ex vivo:*** Porcine buccal mucosa	XG-based films demonstrated 0.292 ± 0.003 to 0.608 ± 0.002 mJ/cm^2^ Work of Adhesion (WoA). Bioadhesive film retention at targeted site increased.	Films contained 0.5% nystatin, 0.4% hydrocortisone acetate, and 0.8% lidocaine HCl. Over 8 h, nystatin released up to ∼51%, hydrocortisone acetate ∼80%, and lidocaine HCl ∼98%.	Arslan et al. (2022)
	Pectin		Fluconazole	Buccal films containing FZ-SLNs	Oral (buccal mucosa)	***In vitro:*** Franz-type diffusion cell and agar diffusion method for investigating drug release and antifungal activity. ***Ex vivo:*** Porcine buccal mucosa for permeation and mucoadhesion	From designed films, FC3 exhibited the highest adhesion (∼0.12 N mm along with the ∼0.057 N detachment force. Improved buccal residence time was reported.	Initial burst release followed by sustained release behavior was monitored. Cumulative final drug release for FC3 was the highest (∼67%) at 6 h.	Hirun et al. (2024)
			N/A	Hydrogel films	Topical (skin) → wound dressings	***In vitro:*** Kirby–Bauer disc diffusion method for investigating the antimicrobial activity against *Staphylococcus aureus*. ***In vivo:*** Streptozotocin-induced diabetic Sprague Dawley (SD) rats for analyzing the wound healing behavior.	Not reported	Not reported	Nordin et al. (2022)
	Hyaluronic acid (HA)		Latanoprost	Liposome suspension with 0.2% w/v HA	Ocular (topical eye drops)	***In vitro:*** hTERT-HCECs cells and IM-HConEpiC cells. ***In vivo:*** Normotensive albino male rabbits.	Drug concentration was measured in rabbit tear fluid (up to 60 min) for indirect assessment of the drug's ocular surface retention.Designed LAT-HA-LIP formulation demonstrated drug concentrations of 32.45 ± 11.21, 25.00 ± 4.12, and 25.75 ± 7.37 μg/mL at 10, 30, and 60 min, respectively. It was claimed that no significant statistical difference was found in comparison to the commercial benchmark Monoprost®.	*In vitro* drug release was not reported. However, onset of action was noticed at 1 h post-instillation, with the peak effect at 3 h, showing a maximum intraocular pressure reduction of 24.02 ± 8.26% for LAT-HA-LIP. Response lasted for 48 h after single application.	Brugnera et al. (2024)
			Dimethyl fumarate	Lipid-polymer hybrid NPs	Intranasal → nose-to-brain	***In vitro:*** Human nasal septum squamous cell carcinoma cell line and human neuroblastoma cell line was used as cell models. PermeaPad® plate was incorporated as an *in vitro* barrier model for evaluating permeation behavior. ***In vivo:*** Male SD rats.	LPH showed higher mucoadhesion ∼1650 Pa as compared to LPHD ∼1360 Pa. Quantitative residence time was not reported. However, hyaluronic acid-formulated NPs exhibited high dynamic viscosity (183 ± 0.4 cP for LPH and 134 ± 1.3 cP for LPHD), which may help reduce mucociliary clearance and prolong nasal retention.	Clear *in vitro* drug release data were not reported. *In vitro* permeation was evaluated on PermeaPad® over 5 h, with mucin LPHD showing higher permeation (2.5 × 10^−7^ cm/s) as compared to LPD (1.2 × 10^−7^ cm/s.	Serri et al. (2025)
Semi-synthetic	HEC		N/A	Methacrylated HEC containing mucoadhesive wafers	Oral mucosa (buccal cavity)	***In vitro:*** Caco-2 cell line. ***Ex vivo:*** Buccal mucosa from freshly excised sheep upper and lower lips. ***In vivo:****Schmidtea mediterranea* planaria	Glycidyl methacrylate-modified HEC showed a 3.27-fold (∼0.54 N) increased adhesion force in contrast to unmodified HEC. Transmucosal residence time tends to increase because of chemically modified HEC.	Not reported	Buang et al. (2023)
			Insulin	Hydrogel with drug-loaded liposomes	Nasal mucosa → nose-to-brain/nose-to-systemic delivery	***In vitro:*** Franz diffusion cell. ***In vivo:*** Streptozotocin-induced type-I diabetic male Wistar rats.	Increased retention time in nasal mucosa was observed.	Hydrogel with insulin-loaded liposomes demonstrated slowed and sustained drug release. After 24 h. HEC + insulin-loaded liposomal formulation showed 59.69% of drug release. Moreover, TAT- and penetratin-functionalized liposomes showed even more slowed drug release behavior 42.46% and 41.83%, respectively.	Von Zuben et al. (2022)
	HPMC		Meloxicam	NPs (mucoadhesive, thermoresponsive, and nanostructured system)	Nasal mucosa → nose-to-brain	***In vitro:*** Franz diffusion cell ***In vivo:*** Porcine nasal mucosa for mucoadhesion evaluation and *Artemia salina* (brine shrimp) nauplii for cytotoxicity investigation.	Optimized formulation F2 [17.5% P407, 0.10% HPMC, 0.05% meloxicam] illustrated a detachment force of ∼0.30 N and WoA of ∼0.43 N mm (*ex vivo*), presenting better adhesion to nasal mucosa in comparison to drug-free formulation.	Biphasic drug release behavior was observed, initial burst release (>60% at 15 min) followed by slow and sustained release (∼94% up to 480 min).	Botan et al. (2024)
			Benzophenone-4	Hydrogels containing HPMC or HMPC-PVP	Skin → topical application	***In vitro:*** Dialysis tube (MW cut-off: 14,000 Da). ***Ex vivo:*** Porcine skin	Hydrogel formulations (HPMC–PVP), H12P6 and H12P9 exhibited 6.19-fold (253.64 ± 14.87 g.s) and 9.89-fold (405.08 ± 13.17 g.s) higher adhesiveness as compared with HPMC 12% alone. Reported values indicated the improved skin retention and contact.	HPMC homopolymer gels showed sustained, approximately zero-order release of benzophenone-4 during the first 6 h, with the release rate decreasing from 8.9%/h (12% HPMC) to 7.2%/h (13% HPMC) as polymer concentration increased. The H12P6 binary hydrogel showed extremely restricted drug release, with no detectable benzophenone-4 released over 24 h; cumulative release remained below the LOD of 1.08 μg/mL.	Pan et al. (2023)
	Na-CMC		Sumatriptan succinate	Mucoadhesive microspheres	Nasal cavity	***Ex vivo:*** Goat nasal mucosa strip	Optimized formulation – F6 (contained 220.71 mg SCMC and 150 mg HPMC K15M) showed highest and most consistent mucoadhesion in comparison to tested formulations, thereby facilitating prolonged mucosal retention.	Highly consistent and uniform drug release was demonstrated by F6 with a cumulative discharge rate of 94.07 ± 1.85% over 8 h.	Vyas et al. (2024)
			Gemcitabine and papain	Mucoadhesive gel	Bladder wall → intravesical (bladder cancer)	***In vitro:*** Human endothelial and fibroblast cell lines for biocompatibility evaluation. Porcine stomach mucin for rheological evaluation. ***Ex vivo:*** Porcine bladder tissue.	Gel formulation (containing gellan gum + nanopapain) exhibited better mucosal retention than plain gellan gum, with a bioadhesive force of −0.170 Pa at 4.5 s^−1^. Gellan gum formulation demonstrated better wash-off resistance as compared to CMC + PVA gels.	Gellan gum formulation showed sustained release over the first 6 h, though releasing faster than the CMC + PVA formulations. Formulation C4 CMC + PVA, achieved 97.2 ± 1.5% drug release within 24 h.	de Lima et al. (2023)
Synthetic	PEG		Clotrimazole	Drug-loaded polymeric micelles into hydrogel	Vaginal mucosa → vaginal candidiasis	***In vitro:*** HEK293T cells (cytotoxicity analysis) and *C.albicans* (antifungal activity evaluation).	Adhesive properties of the PEG-PCL-PEG hydrogel prolonged the drug's residence time.	Hydrogel containing clotrimazole-loaded micelles showed slow and sustained release behavior with a cumulative drug release of 44.8% up to 6 days.	Hosseinzadeh et al. (2023)
			Retinyl palmitate, Ketorolac	Mucoadhesive films	Intravaginal	***In vitro:*** Primary culture fibroblasts. ***Ex vivo:*** Porcine uterine tissue	Upon fluid contact, the films formed a mucoadhesive gel, followed by swelling for up to 100 min and complete degradation within 3–4 days. The films also exhibited good resistance to sliding.	Films B and D achieved 100% cumulative release within 30-60 min (containing HPMC (14.97% and 20.94%), PEG 400 (84.30% and 78.38%), and retinyl palmitate (0.73% and 0.67%), respectively). Film G (HPMC - 20.75%, PEG 400 - 77.16%, and ketorolac 2.06%) achieved 100% cumulative release within 2 h. High polymeric concentration slowed drug release.	Hernández-González et al. (2024)
	Carbopol (PAA derivative)		Miconazole nitrate	Drug-loaded bilosomes in Gel	Skin → topical antifungal activity	***In vitro:*** HET-CAM model. *C. albicans* and *Aspergillus niger* fungal strains.	Not reported	Biphasic drug release behavior, initial rapid release followed by sustained release. At 24 h, the cumulative drug release of optimized bilosomes and optimized bilosomes gel reached 94.76 ± 3.7% and 58.54 ± 4.1%, respectively.	Imam et al. (2023)
			Voriconazole and Secnidazole	Mucoadhesive vaginal gel	Vaginal mucosa → bacterial and fungal infections	***In vitro:*** Cup plate agar diffusion method along with fresh cultures of *S. aureus* and *C. albicans* for antimicrobial activity. ***Ex vivo:*** Sheep vaginal mucosal tissue	Optimized formulation (F8) exhibited 1795.36 ± 25 dyn/cm^2^ detachment force. Mucoadhesive gel showed mucoadhesion for >5 h.	Drug release was investigated for 2 h; optimized formulation (F8) showed sustained release behavior with 98.98% and 100% cumulative release of secnidazole and voriconazole.	Vr et al. (2024)
	Thiolated polymers	Thiolated XG	Miconazole nitrate	Thiolated XGNPs	Intestinal mucosa via oral route	***In vitro:*** Caco-2 cell line (cytocompatibility) and *C. albicans* using agar-well diffusion (antifungal activity). ***Ex vivo:*** Sheep intestinal mucosa (mucoadhesion) and albino rat intestinal mucosa (permeation study) ***In vivo:*** Albino rats.	TXGM and NTG1 (optimized formulation) exhibited 24.8 and 24.4 h, *ex vivo* mucoadhesion in contrast to unmodified xanthan gum (6.2 h).	Optimized thiolated polymeric NPs formulation gave sustained and controlled drug release (90.5% at 24 h).	Namazi et al. (2024)
		Thiolated CS	Tobramycin	Thiolated CSNPs	Ocular surface	***In vitro:*** ARPE-19 human retinal pigment epithelial cell line (cytotoxicity). Nutrient agar plates inoculated with *P. aeruginosa* (antibacterial activity). ***Ex vivo:*** Sheep corneas and eyelids. ***In vivo:*** Sheep	Thiolated CS and optimized formulation (NT8) exhibited 7.6 and 7.8 ± 0.54 h, *ex vivo* mucoadhesion, respectively.	The optimized formulation achieved a maximum sustained cumulative release of over 90.34% at 8 h.	Javed et al. (2023)

*Quantitative values are reported only when explicitly provided in the original studies to ensure accurate representation of the reported data. CNS: Central nervous system, ζ potential: Zeta-potential, CS–GQD: CS–graphene quantum dot, HEC cells: Human corneal epithelial cells, CaCO_3_: Calcium carbonate, HCl: Hydrochloride, XG: Xanthan gum, FZ-SLNs: Fluconazole-loaded solid lipid nanoparticles, CMC: Carboxymethyl cellulose, PVA: Poly(vinyl alcohol), PEG: Polyethylene glycol, GDL: glucono-δ-lactone, XG-pNIPAM: Xanthan gum/poly-N-isopropylacrylamide, AMP14d: 14-amino-acid antimicrobial peptide, hTERT-HCECs: Human immortalized corneal epithelial cells, IM-HConEpiC: Human immortalized conjunctival epithelial cells, LAT-HA-LIP: Latanoprost-hyaluronic acid-liposome formulation, LPH: Lipid-Polymer nanoparticles + Hyaluronic acid, LPHD: Lipid-Polymer nanoparticles + Hyaluronic acid + Drug, P407: Poloxamer 407, PVP: Polyvinylpyrrolidone, HPMC: Hydroxypropyl methylcellulose, H12P6: HPMC-12% w/w and PVP-6% w/w, H12P9: HPMC-12% w/w and PVP-9% w/w, SCMS: Sodium carboxymethylcellulose, HEK293T: Human embryonic kidney cells, PCL: Poly(ε-caprolactone), HET-CAM: Hen's egg test-chorioallantoic membrane model, *C. albicans*: *Candida albicans*, *S. aureus*: *Staphylococcus aureus*, TXGM: Thiolated xanthan gum, NTG1: Miconazole-loaded thiolated xanthan gum nanoparticles, *P. aeruginosa*: *Pseudomonas aeruginosa*, CS: Chitosan, NT8: Nanoparticulate formulation of tobramycin + thiolated chitosan.

The natural polymers are particularly obtained from natural sources and mainly include CS, alginate, xanthan gum, tragacanth, acacia, cellulose, agarose, carrageenan, gelatin, pectin, hyaluronic acid, and starch (Zhao et al., 2022). These polymers have ideal properties, such as biodegradability, biocompatibility, low toxicity, and being less expensive, and they allow better adhesion properties without chemical modifications (Yermak et al., 2022). However, the batch-to-batch variation, susceptibility to microbial contamination, and the fact that few have restricted stability or mechanical strength have hampered their full potential and brought about the development of semi-synthetic and synthetic polymers (Garg et al., 2018).

The semi-synthetic polymers are chemically modified natural polymers that attain the favorable attributes of natural polymers along with the enhancement of stability, mechanical strength, and better adhesiveness with desired drug release behavior (Hossain et al., 2025; Ruso et al., 2017). For instance, cellulose derivatives like methyl cellulose (MC), HEC, HPC, HPMC, CMC, and Na-CMC are mainly employed as semisynthetic polymers in MDDS (Germershaus et al., 2015). However, the extent of chemical modification may introduce toxicity and reduce the biodegradability of polymers (Alopaeus et al., 2020).

Furthermore, the synthetic polymers, i.e., PEG, carbopol, polycarbophil, PVP, PVA, PAA, and poly(Hydroxyethyl Methacrylate) (pHEMA), are man-made products designed via manipulation of other polymers or compounds (Ugoeze, 2020). These polymers offer significant stability, mechanical strength, and mucoadhesiveness; for instance, in order to acquire enhanced mucoadhesion and prolonged residence time, PAA derivatives deliver a remarkable contribution (Nafee et al., 2004). Moreover, functionalization of these polymers via thiol, catechol, and acrylate groups brings strong mucoadhesive features (Brannigan et al., 2019). However, in contrast to natural polymers, the synthetic ones are possibly less biocompatible and biodegradable (Zhao et al., 2022).

Apart from conventional polymeric classification, emerging strategies like hybrid polymeric systems and interpenetrating polymeric networks facilitate the merging of complementary material features within a single platform. Hybrid polymer networks, like combinations of CS and alginate, have gotten significant attention as highly versatile systems that overcome the processing and performance limitations of their individual natural components (Yang et al., 2013; Kilicarslan et al., 2018). Kilicarslan et al. designed a clindamycin-loaded polyelectrolyte complex films by ionic cross-linking between cationic CS and anionic alginate for local periodontal therapy. After detailed investigation, it was concluded that the designed films assisted in protecting the acid-sensitive active ingredient, delayed drug release, and improved localized drug retention (Kilicarslan et al., 2018). Yang et al. constructed an interpenetrating polymer network by using methoxy PEG-grafted carboxymethyl CS (mPEG-g-CMC) and alginate to design pH-sensitive hydrogel beads. While evaluating, physically mixed mPEG/CMC hydrogels were compared with mPEG-g-CMC/alginate hydrogel, which exhibited higher protein loading capacity. The mPEG-g-CMC/alginate hydrogel slightly reduced burst release at acidic pH (1.2) and improved release at pH 7.4. These features highlighted the potential of a designed system for site-specific protein delivery to the intestine (Yang et al., 2013). Furthermore, excellent mucopenetration was observed for CS-alginate polyelectrolyte complex NPs loaded with amoxicillin for *H. pylori* eradication (Arora et al., 2011).

### 4.2 Functionalization for enhanced adhesion and controlled release

Mucoadhesive polymers are often functionalized for enhancing the performance of MDDS in order to achieve a controlled drug release profile and improve adhesion behavior. For this purpose, surface modification (Sepulveda-Cordova et al., 2024), incorporation of bioactive agents (Gabor et al., 2004), utilization of smart polymeric networks (Xiao et al., 2021; Gao et al., 2025), and nanotechnology integration are being extensively explored to improve mucoadhesion, therapeutic efficiency, and control drug release, as illustrated in Fig. 3.

**Fig. 3:**
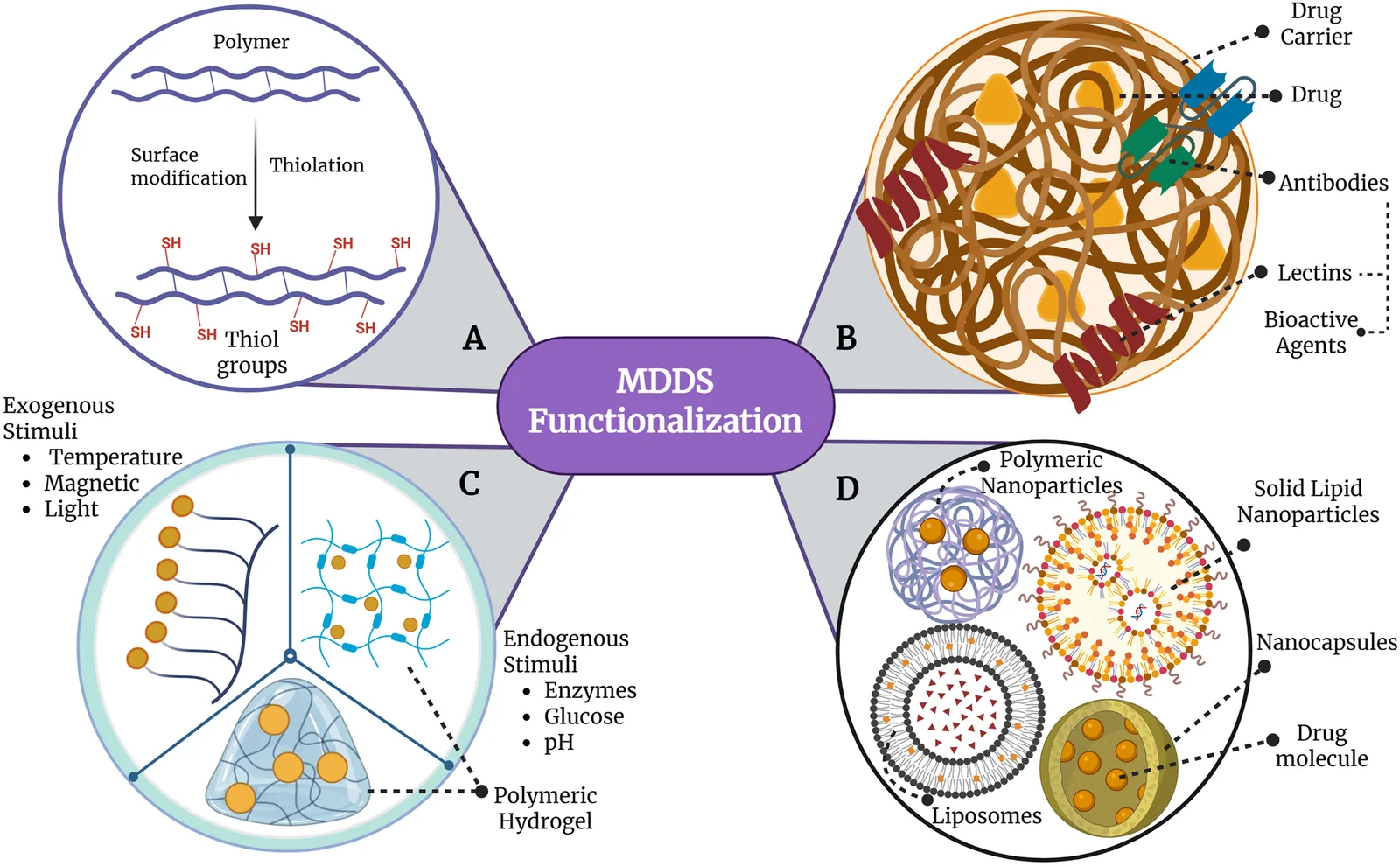
Schematic illustration of MDDS functionalization: [A] polymeric modification via thiolation which incorporates thiol groups to polymer; [B] incorporation of bioactive agents or ligands into drug carriers to assist site-specific binding; [C] smart (stimuli-responsive) polymers that facilitate controlled and targeted drug delivery; and [D] integration of nanocarriers along with MDDS that facilitates better drug encapsulation, improved retention time, and regulation of drug release behavior.

#### 4.2.1 Surface modification via thiolation

Thiolation is a promising approach for surface modification of polymers. After modification, thiol groups holding polymers–thiomers are formed. These thiomers are capable of developing disulfide bridging with cysteine-rich domains of mucus glycoprotein (Cook et al., 2024), because the sulfhydryl (-SH) moieties are introduced onto the backbone of preformed polymer (Ghosal et al., 2024). This kind of modification not only improves mucoadhesion but also contributes to enhanced permeation (Leichner et al., 2019). Davoudi et al. conducted a study and designed thiolated corn starch to explore its mucoadhesion and permeation properties (Davoudi et al., 2025). After evaluation, it was found that the modified starch has adequate thiol groups and demonstrated no cytotoxicity. Moreover, the mucoadhesion and permeation were improved by 91.1-fold and 2-fold, respectively, as compared to unmodified starch. In conclusion, it was summarized that the remarkable improvement in mucoadhesion and permeation was delivered by modified starch, highlighting its potential as a suitable excipient for mucosal drug delivery.

However, thiomers with free thiol (-SH) groups are highly susceptible to oxidation after swelling in water; consequently, the premature oxidation of thiol moieties reduces their availability at the action site (Kast et al., 2001). To conquer this challenge, researchers introduce the concept of preactivated or S-protected thiomers (Basu et al., 2025). In preactivated thiomers, S-protecting ligands such as 6-Mercaptonicotinic Acid (6-MNA) (Perrone et al., 2017), L-cysteine, and NAC are employed to preserve thiol reactivity by locking them in reversible disulfide bonds, which are later exchanged with mucin thiols to establish stronger, more stable adhesion (Shahzadi et al., 2020; Wibel et al., 2021). A study was performed to improve the oral delivery of peptides via MNA-activated thiolated CS (TG-CS) to support non-invasive delivery that ensures patient comfort (Pratap-Singh et al., 2023). The incorporation of MNA-activated TG-CS into insulin NPs greatly increased both mucoadhesion and permeation compared to unmodified and TG-CS systems. Pre-activation of MNA stabilized the thiol groups and facilitated better disulfide exchange with mucins, resulting in sustained and improved mucoadhesion at the mucosal level. Residence time was increased not just by this improved interaction but also by greater exposure to epithelial tissues. Simultaneously, the formulation was found to have increased permeation capacity as reflected by significantly higher delivery of insulin across buccal cell monolayers and higher absorption by HepG2 cells. The increased permeability was attributed to MNA-TG-CS's potential for transient opening of tight junctions, as reflected by a highly significant reduction in trans-epithelial electrical resistance. Collectively, these findings confirm that the dual mechanism of intensified mucoadhesion and tight junction modulation is responsible for the increased delivery effectiveness of MNA-TG-CS–based NPs (Pratap-Singh et al., 2023). Table 3 indicates the comparative mucoadhesive properties of thiolated and non-thiolated polymers.

**Table 3:** Comparative mucoadhesive properties of thiolated and non-thiolated polymers.

Polymer	Modification	Mucosal/Mucin Model	Relative improvement	Experimental Method	References
Alginate	Covalent attachment of cysteine to alginate	Porcine intestinal mucosa	Total Work of Adhesion (TWA) of 25.8 ± 0.6 and 101.6 ± 36.1 μJ for alginate and the alginate–cysteine conjugate, approximately 4-fold higher.	Tensile analysis	Bernkop-Schnürch et al. (2001)
PAA	PAA-cysteine conjugate	Native porcine intestinal mucosa	3-7-fold stronger mucoadhesiveness was observed for the PAA-cysteine conjugate tablets compared to unmodified PAA tablets. For both thiolated and unmodified polymers, the mucoadhesiveness was 2-4-fold enhanced below pH 5.	Mucoadhesion studies	Guggi et al. (2004)
PAA	PAA-cysteine conjugate I (90.5 ± 15.8 μmol thiol groups/g polymer) and II (511.6 ± 52 μmol thiol groups/g polymer).	Intestinal porcine mucosa	Tablets based on conjugate II remained attached to freshly excised porcine mucosa for about 25 times longer than unmodified PAA.	Mucoadhesion studies	Marschütz et al. (2002)
CS	TBA-CS; TBA-CS 54; TBA-CS 208; TBA-CS 264.	Intestinal porcine mucosa	TWA of TBA-CS 54 was increased 1.3-fold compared to unmodified CS. TBA-CS 208 and 264 showed a 39- and 118-fold increase in the TWA. The contact time of TBA-CS conjugates on mucosal tissue increased with the increasing amount of thiol groups covalently attached to the polymer from 2.7 h up to more than 360 h. The greatest improvement was achieved with medium-molecular-mass TBA-CS 264, which showed an improvement ratio of more than 277.	Tensile studies; mucoadhesion analysis	Roldo et al. (2004)
CS	l-cysteine-modified thiolated CS (CS-Cys), 6-MNA-S-protected thiolated CS (CS-Cys-MNA), and l-cysteine-S-protected thiolated CS (CS-Cys-Cys)	Porcine intestinal mucosa	The strength of mucus interaction followed the rank order plain; < CS < CS-Cys-Cys < CS-Cys < CS-Cys-MNA. Mucus diffusion followed the rank order; CS-Cys < CS-Cys-Cys < CS < CS-Cys-MNA < plain.	Mucus retention and mucus diffusion assay	Wibel et al. (2021)

#### 4.2.2 Incorporation of bioactive agents

The site-specific binding of drug carriers to receptors or sugar residues on mucosal epithelial cells is facilitated by the incorporation of bioactive agents or ligands, i.e., lectins, sugars, or antibodies, into drug carriers. This strategy brings two major improvements: (1) prolonging residence time at the targeted site, and (2) these ligands can facilitate the receptor-mediated uptake of drugs into cells. Colaço et al. performed a study (Colaço et al., 2024), in which researchers emphasized nasal vaccine delivery by utilizing glycan ligands, such as mannose and lactobionic acid, to target C-Type Lectin Receptors (CLRs) on mucosal immune cells. By binding to these receptors, lectin-based systems not only promote antigen uptake but also provide site-specific retention within the nasal mucosa, underlining their potential as receptor-mediated mucoadhesive strategies. It was summarized that targeting the CLRs assists in designing more effective mucosal delivery (Colaço et al., 2024). Furthermore, alginate is an anionic mucoadhesive polymer extensively utilized for drug delivery, 3D bioprinting, and tissue engineering. Functionalization of alginate with lectin facilitates site-specific drug delivery and enhances bioavailability because the alginate-lectin conjugate can resist acidic and enzymatic degradation while providing receptor-mediated adhesion to mucosal surfaces (Putri et al., 2021). Research was performed by Li et al. (2016), in which specialized doxorubicin-loaded polymer micelles were designed; after that, these micelles were conjugated with Concanavalin A, a lectin, to deliberately enhance the mucoadhesion of these specialized micelles. After evaluation, it was observed that lectin-conjugated micelles improved adhesion to the mucosal surface and released the drug in a controlled manner, highlighting the potential of this approach for improving mucoadhesion and effective drug delivery.

#### 4.2.3 Smart (stimuli-responsive) polymers for controlled and targeted drug delivery

Smart polymeric networks can respond to local stimuli such as pH and temperature, thereby modulating drug release kinetics (Tian et al., 2025). The stimulus-generated modifications in swelling, mucoadhesion, or drug release for specific delivery routes facilitate defining the translational potential of these systems.

In a study, hyaluronic acid was cross-linked with methacrylic and acrylic acids to formulate mesalamine-loaded hydrogels with the objective of designing a stimuli-responsive delivery system for colonic administration. For evaluation, swelling and drug release studies were conducted at 37°C in both acidic and basic media at pH 1.2, 4.5, 6.8, and 7.4. In the results, it was observed that the system stayed stable in acidic conditions and swelled and released the drug at pH 7.4, which indicated the pH-sensitive response of the designed hydrogels. The hyaluronic acid, methacrylic acid, and acrylic acid concentrations are the crucial factors that influenced swelling, drug loading, and release behavior. Overall, this study explained that the pH-responsive systems have an adequate potential for targeted drug delivery in a controlled way at higher colonic pH (Liaqat et al., 2024).

Further quantitative evidence has been reported for pH-sensitive and mucoadhesive semi-interpenetrating polymer networks (semi-IPN). Ajaz et al. fabricated the semi-IPN for dexamethasone sodium phosphate colonic delivery by using HPβ-CD-g-PAA/gelatin (Ajaz et al., 2022). The designed system exhibited pH-dependent swelling behavior and mucoadhesive features. *In vitro* investigation revealed the pH-dependent drug release kinetics, as at pH 1.2 only a minute drug fraction was released; however, 90.58% of the drug was released at pH 7.4 over 72 h. The increased release at the higher pH was associated with ionization of carboxylic groups, enhanced swelling, and electrostatic repulsion within the polymer network. Moreover, the biocompatibility of the designed system was confirmed after showing non-toxic effects on the rabbit's vital organs (Ajaz et al., 2022).

Moreover, in another study, researchers employed phenylalanine-phenylalanine dipeptide for cross-linking the N-acetylated CS and alginate in order to fabricate the pH-responsive hydrogel system. The hydrogels demonstrated high drug release behavior, e.g., 51% of hydrocortisone and 77.6% of sulfasalazine within 5 h in a colon-simulated pH environment. Release kinetics indicated targeted and controlled drug release by the pH-responsive system (Khatibi et al., 2024). However, for performance evaluation, it is essential to consider the desired degree of swelling along with the system integrity, mucoadhesion, and drug release behavior.

Furthermore, a thermoresponsive Pluronic F127 gel combined with thiolated glycol CS derivatives and loaded with gemcitabine and celecoxib demonstrated strong mucoadhesion and prolonged drug release in intravesical delivery, highlighting the significant potential of stimuli-responsive and mucoadhesive polymers in cancer therapy (Ricci et al., 2023). In contrast to pH-sensitive systems intended for targeting the GIT, thermosensitive systems can use body temperature to achieve in situ gelation and increased retention at the bladder surface, as an example of the importance of selecting stimuli that match the biological environment and mode of delivery (Ricci et al., 2023).

#### 4.2.4 Harnessing nanotechnology for MDDS

Nanocarriers are suitable drug carriers for drug encapsulation, enhance their physical stability, and facilitate their efficient delivery by overcoming the conventional delivery system challenges (Chamundeeswari et al., 2019). However, the coating of NPs with mucoadhesive polymers ensures better mucoadhesion, prolonged retention, and a controlled drug release pattern (Lugli et al., 2025). R. Jalal et al. experimented with efficient delivery of ciprofloxacin via mucoadhesive methacrylated CS-NPs (Jalal et al., 2023). It was concluded that methacrylated CS-NPs have been shown to enhance encapsulation efficiency, sustain drug release, and increase mucosal retention compared to unmodified CS-NPs, demonstrating the significance of nanotechnology integration in strengthening the MDDS. In another study, a team of researchers designed an intranasal in situ gel by incorporating topiramate-loaded PLGA-NPs (Tanna et al., 2024). An initial burst release followed by sustained release for the next 24 h was monitored. The increased drug delivery from nose-to-brain showed the effective mucoadhesion and permeation of the gel, underscoring the potential of NPs integration in MDDS for enhancing the therapeutic effectiveness via targeted and controlled drug delivery. The incorporation of NPs in MDDS is an important tool to develop delivery systems with enhanced mucoadhesion and permeation features that directly reflect the performance of MDDS, as evidenced by extensive scientific research.

### 4.3 Regulatory and safety considerations of mucoadhesive excipients

For the successful development of MDDS, it is mandatory to subject them to regulatory scrutiny to address safety and efficacy considerations. MDDS are borderline products that are primarily categorized by their principal mode of action (Directive, 1993). That's why they have the status of Substance-Based Medical Devices (SB-MD) under the European Regulation for Medical Devices 2017/745 (MDR), which was fully implemented in May 2021 (Vigani et al., 2023). Additionally, the weak, reversible, and non-specific interactions are responsible for mucoadhesion mechanisms along with other effects like swelling and hydration, which support their classification as medical devices, as stated in Medical Device Coordination Group (MDCG) 2022—5 guidance under European Regulation (EU) 2017/745. SB-MDs are generally classified as invasive devices. For mucoadhesive polymers, due to their typically high MW and non-systemic absorption, they usually fall into Class IIa or IIb under Rule 21 of the new regulation, assuming they are medical devices. Moreover, according to the FDA, products that lie on the border between drugs and devices are handled as combination products (FDA, 2018).

It is evident in the literature that natural and semisynthetic polymers like hyaluronic acid, CS and cellulose derivatives often have favorable safety profiles because of their biocompatibility, biodegradability, and low toxicity (Serrano-Sevilla et al., 2019). In contrast, synthetic or modified polymers like thiomers are more vulnerable and require extensive evaluation for safety, toxicity, irritation, and immunogenicity. For instance, Linear Polyethylenimine (L-PEI) is used in pharmaceutical formulations, but the presence of a secondary amine group in L-PEI exhibits low biocompatibility and a high toxicity profile (Fu et al., 2024). This emphasizes that synthetic mucoadhesive polymers need careful investigation in terms of safety and efficacy. Additionally, it is essential to characterize the physicochemical features of mucoadhesive polymers, i.e., solubility, purity, degree of functionalization, and MW, because of their direct influence on the swelling behavior, mucoadhesion, residence time, safety, and toxicity (Vigani et al., 2023).

## 5 Patient-centric advantages of mucoadhesive drug delivery

To interact closely with mucosal surfaces is the prime intent in designing MDDS that directly address patients’ unique needs and preferences by offering several advantages (Pandey et al., 2023). Unlike traditional dosage forms, MDDS offer a combination of benefits that primarily include reduced dosing frequency, improved bioavailability, controlled drug release, targeted drug delivery, user-friendly administration, and better therapeutic outcomes (Alawdi et al., 2021; Rai et al., 2023). Fig. 4 illustrates the patient-centered benefits of MDDS.

**Fig. 4:**
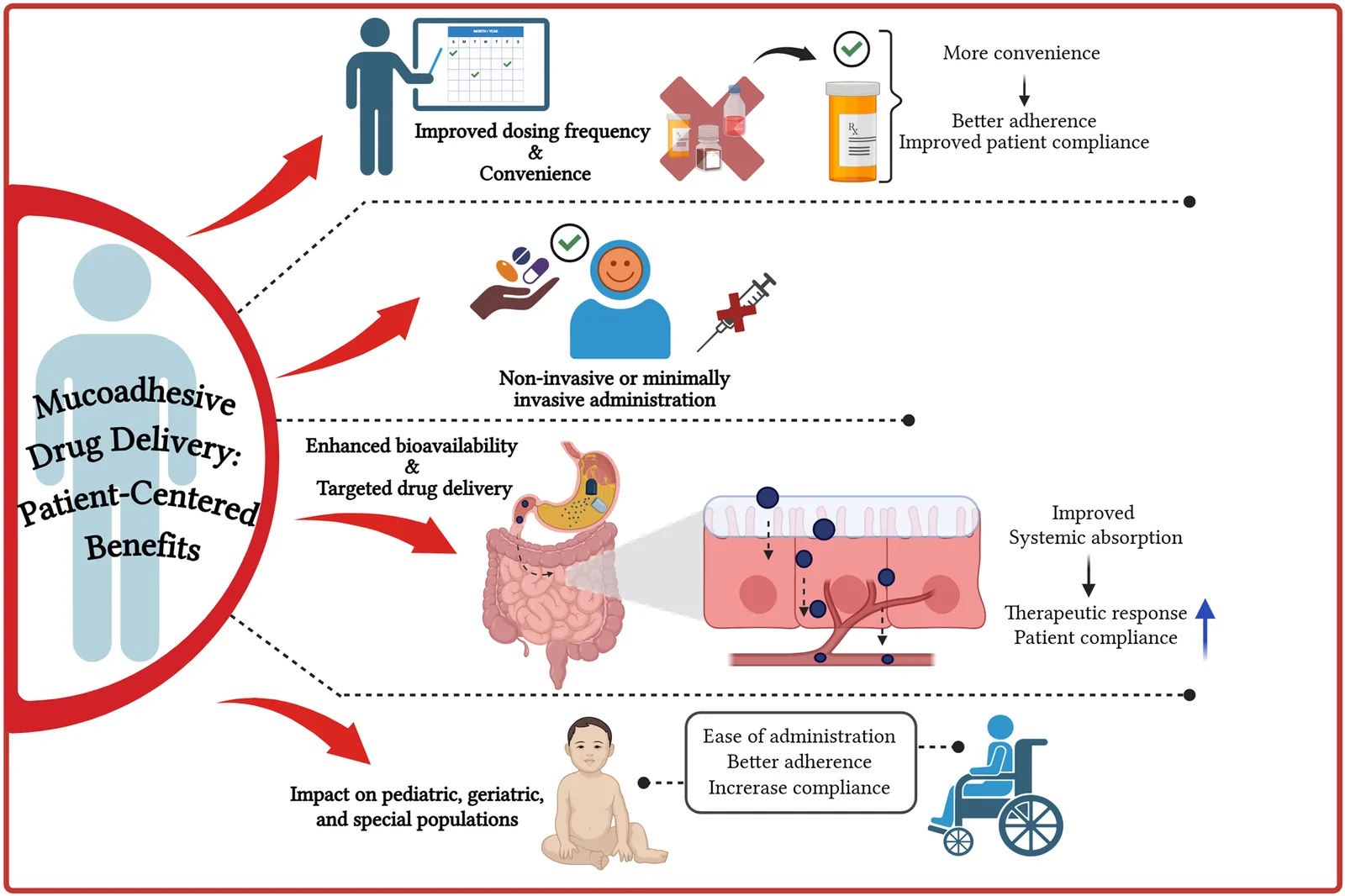
Visual demonstration of patient-centered benefits of mucoadhesive drug delivery.

### 5.1 Improved dosing frequency and convenience

Maintaining sustained drug release at the application site is one of the chief patient-centric advantages of MDDS. The formulation develops prolonged contact with the mucosal surface, which ensures gradual drug release. This reduces the need for frequent dosing, which improves patient compliance and reduces the risk of missed doses. Additionally, in chronic situations, it facilitates improving QoL because of fewer administrations that lessen the treatment burden. A study was conducted by Sugio et al. (2024) with the aim of assessing the clinical and mycological effectiveness of mucoadhesives for oral candidiasis. In the end, it was summarized that the MDDS have a promising potential for lowering the daily dosage frequency and improving patient compliance as compared to conventional therapies. For oropharyngeal candidiasis, miconazole-containing mucoadhesive tablets reduced the need for conventional miconazole gel application from four times a day to once a day (Bensadoun et al., 2008). Furthermore, in a trial study, it was analyzed that the clinical and mycological effectiveness are the same for both 50 mg miconazole mucoadhesive tablets and 10 mg clotrimazole troches, but mucoadhesive tablets are required once a day instead of five times a day in the case of troches (Vazquez et al., 2010). Oransa et al. performed a study to develop cinnarizine-loaded CS-coated niosomes to enhance gastric residence time and oral bioavailability of the model drug (Oransa et al., 2022). The optimized formulation showed a sustained drug release profile up to 8 h, and the 2.79- and 1.92-fold improved oral bioavailability was observed for the designed formulation as compared to the free drug and uncoated niosomal formulation, meaning that dosing frequency may be lowered. All these scenarios underscore the potential of MDDS for improved dosing frequency and generate convenience for patients, as they didn't require frequent drug administration for therapeutic effectiveness. Moreover, the MDDS is a convenient approach, as it allows maintaining therapeutically effective drug concentration for a sufficient time at the infected area (Adepu et al., 2021). Consequently, these systems are suitable for synthetic and natural drugs and show rapid absorption at the administration site (Uzunoğlu et al., 2021).

### 5.2 Non-invasive or minimally invasive administration

Non-parenteral drug delivery, i.e., oral, buccal, sublingual, ocular, vaginal, and rectal, is facilitated by mucoadhesive systems (Samanthula et al., 2025). Patients usually prefer non-invasive therapies for disease management because they're safer and more convenient for them as compared to invasive procedures such as injectables. A few hurdles, like needle phobia, site injury, pain, and risk of systemic toxicities, make injectables less convenient and can reduce patient adherence (Lambrinou et al., 2020). Consequently, non-invasive or minimally invasive therapies are patient-friendly, and the chances of medication adherence are usually high, which directly facilitates better therapeutic outcomes. In a study, pentoxifylline-loaded mucoadhesive microspheres were designed for non-invasive nasal delivery (Tadavi et al., 2023). The ionic gelation technique was utilized, and microspheres with a particle size ranging from 27.01 to 33.78 μm were obtained. *In vitro* analysis showed the mucoadhesion of microspheres in the range of 76.14-87.58%. At the end, it was claimed that mucoadhesive microspheres have significant potential for effective non-invasive nasal delivery and assist patients in drug administration.

Oral cancer is a global health challenge due to its high incidence, metastasis, and poor prognosis, and traditional treatments like surgery, chemotherapy, and radiotherapy often cause toxicity, disfigurement, and compromised QoL. To address this problem, mucoadhesive nanocarriers play a remarkable role in addressing solubility, targeting, and bioavailability problems, making them a promising strategy for oral cancer therapy (Pandey et al., 2022). Pornpitchanarong et al. designed doxorubicin-loaded catechol-modified CS/hyaluronic acid NPs to treat oral carcinoma (Pornpitchanarong et al., 2020). Fabricated NPs demonstrated strong mucoadhesion and sustained drug release behavior on porcine buccal mucosa. Additionally, it was found that they induced apoptosis more efficiently compared to the free drug. These findings reflect the potential of non-invasive drug delivery to treat oral cancer.

Usually, MDDS is considered a non-invasive strategy for disease management, but sometimes it involves minimally invasive administration as well. Meiser et al. designed microneedle arrays and incorporated them with imiquimod nanocrystals (Meiser et al., 2024). These microneedles exhibited improved drug release and skin permeation, reducing the dose by 93% compared to semisolid forms. They also maintained effective drug levels even with less than 1 h of contact time, which evaded the need for frequent administration and longer contact time. Findings reflect the potential of microneedle arrays for skin drug delivery with enhanced permeation and reduced systemic side effects, improved patient compliance, and therapeutic outcomes (Meiser et al., 2024). For ocular drug delivery, the blood-retinal barrier and corneal impermeability make it difficult to treat ocular problems. Furthermore, patient safety and compliance are also compromised via conventional drug administration (Bao et al., 2024). Liu et al. fabricated hyaluronic acid and polymethyl methacrylate‐based ocular microneedles and incorporated them with manganese oxide nanocluster‐decorated graphdiyne nanosheets for the effective treatment of keratitis (Liu et al., 2024). So, these microneedles aid in enhancing drug delivery across the ocular barrier (bioavailability), exhibit strong antimicrobial and anti-inflammatory activities, and promote corneal healing. Additionally, *in vitro*, *ex vivo*, and *in vivo* studies proved that the designed formulation has better therapeutic effectiveness compared to commercially available ophthalmic voriconazole, without any cytotoxicity and/or microbial resistance (Liu et al., 2024). Overall, microneedles are minimally invasive in contrast to other mucoadhesive systems but are still more convenient and patient-friendly for the management of diverse diseases.

### 5.3 Enhanced bioavailability and targeted delivery

MDDS develop intimate contact with the mucosal surface that facilitates prolonged retention time, enhances drug permeation or absorption, bypasses hepatic first-pass metabolism, and increases systemic bioavailability (Golshani et al., 2022). Furthermore, targeted drug delivery directly to mucosal sites like oral (Kumar et al., 2022), buccal, sublingual, nasal (Shim et al., 2020), ocular, and vaginal (Abidin et al., 2023) remarkably lowers systemic exposure and lessens side effects.

Zhang et al. designed folic acid-modified pectin NPs for oral insulin delivery (Zhang et al., 2022). Folic acid modification aimed to target the enterocytes. Accordingly, transepithelial transport of insulin was promoted via folic acid receptor-mediated pathways. Furthermore, *in vivo* studies demonstrated that the modified NPs prominently reduced the blood glucose level in type I diabetic rats and further enhanced oral insulin bioavailability as compared to unmodified NPs. This study clearly stated the potential of mucoadhesives for targeted delivery and enhanced bioavailability (Zhang et al., 2022). A study performed by Alastal et al. in which researchers formulated dihydroergotamine-loaded CS NPs for intranasal administration, with the aim of improving its systemic absorption for migraine treatment, as this drug is rapidly metabolized after oral administration (Alastal et al., 2023). The designed formulation exhibited initial burst release followed by sustained release, and drug-loaded NPs via intranasal administration demonstrated significantly improved absolute bioavailability of 82.5 ± 12.3% as compared to intranasal drug solution (53.2 ± 7.7%) in male SD rats. Consequently, enhanced bioavailability along with targeted delivery highlights the significant potential of MDDS.

Ocular drug administration is quite difficult because tears and blinking rapidly wash off the drug, and frequent administration is required. Recently, mucoadhesive micellar loteprednol etabonate-loaded eye drops were formulated by the team of researchers to overcome the ocular drug delivery challenges and treat eye inflammation more efficiently (Zheng et al., 2025b). Phenylboronic acid was employed for micelle functionalization that facilitates strong adhesion to the mucin layer of the cornea. The resulting formulation exhibited sustained drug release over 12 days and strong mucoadhesion. Moreover, it was analyzed that the once-daily administration of the designed formulation delivers an equal therapeutic response as the commercially available Oceanside®, which requires four daily doses. These findings highlight the potential of mucoadhesion for improving drug retention, prolonged release, and enhanced bioavailability that directly leads to patient convenience and better therapeutic response (Zheng et al., 2025b).

El-Enin et al. designed salbutamol sulphate-loaded mucoadhesive vaginal core-in-cup tablets to arrest preterm labor and hamper the side effects associated with invasive and non-invasive conventional formulations (El-Enin et al., 2020). Different combinations of mucoadhesive polymers, including carbopol, HPMC, HEC, and PEG, were incorporated. During evaluation, it was observed that formulations like F4, F5, and F6 had HEC, PEG, and Carbopol as the core, respectively, and Carbopol 934 as the mucoadhesive cup demonstrated strong bioadhesion and prolonged drug release. Moreover, F6 proved to be the best among all formulations because of its improved pharmacokinetic profile (Tmax, AUC _(0-24),_ and t_1/2_). So it was summarized that the vaginal mucoadhesive tablets have the promising potential to bypass first-pass metabolism, enhance drug bioavailability, and improve patient compliance (El-Enin et al., 2020).

### 5.4 Impact on pediatric, geriatric, and special populations

Patients with swallowing difficulties and chronic conditions usually desire alternative dosage forms that are easy to administer; similarly, patient groups like children and elderly people often require the same. The customized delivery system is suitable for such patients to enhance medication adherence (Roostar et al., 2025). In one study, Chachlioutaki et al. designed omeprazole-containing buccal foams for patients with swallowing difficulties, i.e., pediatric and geriatric patients (Chachlioutaki et al., 2023). For the formulation, maltodextrin was used as a sweetener along with different mucoadhesive polymers, i.e., alginate, CS, gelatin, and HPMC, to modulate the structural, mechanical, and physicochemical features of buccal foams. The foams containing HPMC and alginate exhibited prolonged residence time, strong mucoadhesion, and improved drug transport nearly 30 times higher than a suspension across porcine buccal epithelium. These mucoadhesive foams can deliver an easy-to-use and patient-friendly alternative for conventional dosage forms that highlights the patient-centric advantages of mucoadhesives.

Furthermore, mucoadhesive buccal films have the potential to minimize pill burden in geriatric patients, enable easy administration, eliminate the need for swallowing, and enable individualized drug therapy (Southward et al., 2025). In one research project, researchers designed the buccal films via 3D printing and loaded them with cannabidiol-NLCs (Abdella et al., 2024). HEC gel containing cannabidiol-NLCs was utilized as ink for 3D printing. Subsequently, the designed films were evaluated for physicochemical and mechanical profiles, release behavior, and predicted *in vivo* performance. A slow and sustained release profile of 84. 11 ± 7.02% in 6 h was shown by the films. Moreover, predicted pharmacokinetic values (AUC_0–10_ h, C_max_, and T_max_) indicated better drug absorption. These findings underscore the potential of buccal films to conquer biopharmaceutical challenges of drugs and facilitate patient compliance with better therapeutic potential.

Pediatric patients usually suffer from oral diseases like gingivitis, periodontitis, aphthous ulcers, and dental caries, particularly in developing countries (Abouhussein et al., 2020). Considering this, Abouhussein et al. fabricated cetylpyridinium chloride mucoadhesive buccal films to improve the conventional treatment strategies. Mucoadhesive films were based on CS blended with HPMC, MC, HEC, and PVA and assessed for physicochemical and mechanical properties, swelling behavior, mucoadhesion, *in vitro* release profile, and microbiological activity. CS films showed the most swelling, while CS-PVA and CS-HEC blends had the best mucoadhesion. PVA blends also had higher strength. The CS-PVA film showed strong antimicrobial activity against *S. mutans*, suggesting it could be a useful treatment for pediatric oral diseases (Abouhussein et al., 2020). All these studies present the potential of mucoadhesives as practical and patient-friendly alternatives for children, older adults, and individuals with swallowing difficulties. Their adaptable formats, taste-masking potential, and reduced dosing needs make them especially suitable for populations with unique therapeutic challenges.

## 6 Industrial perspectives and commercialization

### 6.1 Marketed mucoadhesive products and their performance

In modern times, to address the overlooked clinical needs of patients, many of the mucoadhesive formulations have successfully shifted from experimental development to market authorization, reflecting their practical applications (Bahraminejad et al., 2025). Multiple medical devices and prescription-based mucoadhesive formulations have been approved by the FDA for oral, ophthalmic, topical, and vaginal delivery. In Table 4 commercially available FDA-approved mucoadhesive products and devices are listed along with their key characteristics.

**Table 4:** Key features and market information of FDA-approved mucoadhesive pharmaceutical products.

Product Name (Brand)	Active ingredient/Key Components	Dosage Form	Route of Administration	Technology/Mucoadhesive basis	Company/Developer	Approval/Market Status	Reference
Crinone®	Progesterone	Gel	Vaginal	Polycarbophil-based mucoadhesive gel	Abbvie INC	1997/Prescription	FDA (2013)
Metrogel®	Metronidazole	Gel	Topical	HEC-based mucoadhesive hydrogel technology	Galderma Laboratories LP	2005/Prescription	FDA (2023a)
AzaSite®	Azithromycin	Solution/Drops	Ophthalmic	DuraSite	Thea Pharma INC	2007/Prescription	FDA. AZASITE (2024)
Lialda®	Mesalamine	Delayed release tablet	Oral	Mucoadhesive Multi-Matrix System (MMX) technology	Takeda Pharmaceuticals USA INC	2007/Prescription	(FDA. LIALDA, 2023; Sriram et al., 2021)
Besivance™	Besifloxacin hydrochloride	Suspension/Drops	Ophthalmic	DuraSite	Bausch and Lomb INC	2009/Prescription	FDA. BESIVANCE (2018)
Oravig™	Miconazole	Tablet	Buccal	Lauriad®	Galt Pharmaceuticals LLC	2010/Prescription	(FDA. ORAVIG, 2020; J Tangso et al., 2015)
Suboxone®	Buprenorphine hydrochloride; Naloxone hydrochloride	Film	Buccal, sublingual	PharmFilm®	Indivior INC	2010/Prescription	(Mundhey et al., 2016; FDA. SUBOXONE, 2025)
Belbuca™	Buprenorphine hydrochloride	Film	Buccal	Cellulose, polycarbophil- based mucoadhesive film	Biodelivery Sciences International Inc	2015/Prescription	FDA. Belbuca (2023)
Zubsolv®	Buprenorphine hydrochloride; Naloxone hydrochloride	Tablet	Sublingual	Cellulose-based mucoadhesive co-particles (saliva-swelling)	Orexo US INC	2016/Prescription	(Mundhey et al., 2016; FDA. ZUBSOLV, 2025)
BromSite®	Bromfenac Sodium	Solution/Drops	Ophthalmic	DuraSite	Sun Pharmaceutical Industries Ltd	2016/Prescription	FDA. Bromsite (2023)
Sympazan®	Clobazam	Film	Oral	PharmFilm®	Otter Pharmaceuticals LLC	2018/Prescription	FDA. SYMPAZAN (2024)
Phexxi™	Citric acid + lactic acid + potassium bitartrate	Gel	Vaginal	Mucoadhesive polymer-based delivery system (alginate + xanthan gum)	Evofem INC	2020/Prescription	FDA. PHEXXI (2025)
Xaciato®	Clindamycin phosphate	Gel	Vaginal	Polycarbophil-based mucoadhesive gel	Organon LLC	2021/Prescription	FDA. XACIATO (2023)
Igalmi®	Dexmedetomidine hydrochloride	Film	Buccal, sublingual	Mucoadhesive polymer film (based on polyethylene oxide, HPMC)	Bioxcel Therapeutics INC	2022/Prescription	FDA. IGALMI (2022)
**FDA-Approved/Cleared Mucoadhesive Medical Devices**							
3M Tegasorb™ Thin Hydrocolloid Dressing	No API; contains hydrocolloids	Thin hydrocolloid dressing	Topical	Hydrocolloids-based mucoadhesive dressing	3M Medical Products	1998/FDA 510(k)- K982892, (Predicate: 3M Tegasorb™ Ulcer Dressing), Medical Device	FDA (1998a)
Comfeel® Seasorb Dressing	No API; contains 30% Na-CMC, 70% alginate	Hydrocolloid dressing	Topical	Hydrocolloids-based mucoadhesive dressing	Coloplast Corp.	1998/FDA 510(k)- K983519, (Predicate: Comfeel® Alginate Dressing), Medical Device	FDA (1998b)
Gelclair®	No API; contains purified water, propylene glycol, PVP, sodium hyaluronate, potassium sorbate, sodium benzoate, HEC, PEG-40 hydrogenated castor oil, disodium edetate, benzalkonium chloride, flavoring, saccharin sodium, glycyrrhetinic acid	Oromucosal concentrated gel	Oral mucosa	PVP-based mucoadhesive hydrogel	Sinclair Pharmaceuticals, LTD.	2001/FDA 510(k)-K013056, (Predicate: Radiacare™), Medical Device	FDA (2001)
MuGard™	No API; contains purified water, glycerin, benzyl alcohol, sodium saccharin, carbomer homopolymer a, potassium hydroxide, citric acid, polysorbate 60, and phosphoric acid	Mucoadhesive oral rinse	Oral mucosa	Carbomer-based mucoadhesive hydrogel	Access Pharmaceuticals, INC	2006/FDA 510(k)-K062795 (Predicate: OraMagicRx, Gelclair®), Medical Device	(FDA, 2006; Mugard, 2020)
Gengigel®	No API; contains aqua, xylitol, PEG-40 hydrogenated castor oil, polysorbate 20, sodium phosphate, carbomer (polycarbophil), dichlorobenzyl alcohol, trisodium phosphate, aroma, citric acid, sodium hydroxide, disodium EDTA, glycerophosphocholine	Gel	Oral mucosa	Polycarbophil-based mucoadhesive hydrogel forming a protective mucosal film	Ricerfarma SRL	2007/FDA 510(k)-K053342, (Predicate: Gelclair®), Medical Device	(Gengigel, 2021; FDAa)
Episil®	No API; contains dioleate, soy phosphatidyl choline, ethanol, propylene glycol, polysorbate 80, and peppermint oil	Oromucosal liquid (in-situ bioadhesive gel)	Oral mucosa	Lipid-based self-assembling film	Camurus AB	2011/FDA 510(k)- K101769 (Predicate: Gelclair®), Medical Device	FDA. EPISIL 510 (2011)

Note: Technology information is taken from published literature or company data where not explicitly stated in FDA labeling.

These commercially available mucoadhesive products have been extensively assessed for their performance in clinical settings (Bassi da Silva et al., 2017). To examine their safety, efficacy, and patient compliance, clinical trials play an important role (Osifowokan et al., 2024). These trials provide evidence of their capacity to improve patients' compliance and therapeutic outcomes by increasing the residence time of the drug, ultimately improving drug bioavailability compared to their comparator. Table 5 lists the clinical trials of marketed mucoadhesive products and devices, focusing on their comparative performance for safety, efficacy, and patients’ compliance behavior with their comparator, i.e., a placebo to provide baseline efficacy and safety, another marketed mucoadhesive drug to represent their relative benefit or non-inferiority, and also other conventional drugs.

**Table 5:** Reported performance of marketed mucoadhesive products and medical devices.

Brand name/Active ingredient	Comparator	Dosage Form/Administration Route	Administration Dose/Frequency	Indication	Study Design/Study Duration	No. Of Participants (n)	Monitoring Parameters	Clinical Outcomes	Reference
Igalmi® (Dexmedetomidine HCL)	No comparator/ Placebo	Film / Sublingual	40 μg, 80 μg, placebo / 3 days	Posttraumatic stress disorder and Alcohol use disorder	A phase 1b, placebo-controlled crossover laboratory study / November 2021-May 2023	n = 12 patients included n = 10 patients completed	Sedation, Blood Pressure (BP), trauma-induced anxiety, alcohol craving, subjective effects of alcohol	80 μg linked to sedation and mild changes in BP, no significant AEs, when administered with alcohol did not affect those parameters, lessens subjective alcohol effects, trauma-induced anxiety	Petrakis et al. (2025)
Igalmi® (Dexmedetomidine HCL)	No comparator/ Placebo	Film / Sublingual	30, 60, 90, 120, 180, and 240 μg / twice daily for 7 days	Opioid withdrawal symptoms	Multi-site, phase 1b/2, randomized, double-blind, placebo controlled trial / 30 days (2 phases)	n = 225, n = 90 discontinue, n = 135 participated	Treatment-Emergent Adverse Events (TEAEs), Clinical Opiate Withdrawal Scales (COWS) Subjective Opiate Withdrawal Scales (SOWS), Agitation and Calmness Evaluation Scale (ACES)	Study supports use of Igalmi, Higher doses i.e. 180, 240 μg, shows ↑ frequency of orthostatic hypotension, hypotension, and somnolence, Doses (120, 180, 240 μg) causes ↓ in COWS and SOWS scores, In one participant with 120 μg bradycardia and orthostatic hypotension became severe, overall TEAEs were mild or moderate in severity. Mean score on the ACES: n = 3 were mild, n = 4 were normal, and n = 5 showed mild calmness	Jones et al. (2023)
AzaSite® (Azithromycin)	0.3% Tobramycin four times daily for 5 days	Ophthalmic solution / Ophthalmic	1% Azithromycin / **Days 1–2:** twice daily **Days 3–5:** once daily	Bacterial conjunctivitis	Prospective randomized, active-controlled, double-masked, phase III trial / August 6, 2004, to October 6, 2005	n = 743 (n = 378 in tobramycin group and n = 365 in AzaSite group)	Ocular signs and symptoms, Bacterial infection recurrence, Rate of microbial eradication, AEs	AzaSite is well tolerated and safe, showing equivalent performance to tobramycin but at less frequent doses. Reported AEs were conjunctival hyperemia, Eye irritation, and worsening bacterial conjunctivitis. All parameters were same in both formulations.	Abelson et al. (2007)
AzaSite® (Azithromycin)	Warm compresses	Ophthalmic solution / Ophthalmic	1% Azithromycin/ 1 drop twice daily for first 2 days followed by once daily for next 12 days + Warm compresses	Posterior blepharitis	Phase IV, randomized, parallel- design, open-label study / 2 weeks	n = 21 patients included n = 20 patients completed	Degree of overall symptomatic relief, Severity of eyelid redness, eyelid debris, eyelid swelling, Meibomian Gland (MG) secretion, and MG plugging	Patients in the AzaSite group show no safety issues and excellent results, improving eyelid redness, MG secretions (become normal in 2 patients), and MG plugging (completely resolved in 3 patients)	Luchs (2008)
AzaSite® (Azithromycin)	DuraSite vehicle	Ophthalmic solution / Ophthalmic	1% Azithromycin / Days 1–2: twice daily Days 3–5: once daily	Bacterial conjunctivitis	Prospective, randomized, vehicle-controlled, parallel-group, double-masked multicenter clinical study / 5 days	n = 279 (n = 130 AzaSite group and n = 149 in Vehicle group)	Ocular signs and symptoms, Bacterial infection recurrence, Rate of microbial eradication, AE	AzaSite is well tolerated and safe for children and adults; 88.5% bacterial eradication was observed. Reported AEs were same in both groups.	Abelson et al. (2008)
Belbuca™ (Buprenorphine hydrochloride)	No comparator/ Placebo	Film / Buccal	Titration phase: 150–450 μg every 12 h/≥14 days, Randomized dose:≥150 μg BID (individualized stable dose, for ≥2 weeks)	Moderate to severe chronic low back pain (CLBP)	Multicenter. double-blind, placebo-controlled, enriched-enrollment, randomized-withdrawal study / ≥6 months	Open-Label Titration: n = 749 Double-Blind Treatment: Belbuca; n = 229, Placebo; n = 232	AE, analgesic efficacy of Belbuca	Belbuca™ is safe and well-tolerated in opioid naive patients requiring all-time opioid treatment for CLBP; AE were nausea, constipation, and vomiting	Rauck et al. (2016)
Belbuca™ (Buprenorphine hydrochloride)	Placebo buccal film	Film / Buccal	Titration phase: 150–900 μg every 12 h/titrated every 4-8 days up to 8 weeks, Randomized dose: ≥150-900 μg every 12 h + rescue medication	CLBP	Multicenter, double-blind, placebo-controlled, enriched-enrollment, randomized-withdrawal study / 7 months	Open-Label Titration: n = 810 Double-Blind Treatment: Belbuca; n = 254, Placebo; n = 257	AE, analgesic efficacy of Belbuca	Belbuca™ is safe and well-tolerated in opioid experienced patients requiring all-time opioid treatment for CLBP; AE was vomiting	Gimbel et al. (2016)
Belbuca™ (Buprenorphine hydrochloride)	No comparative	Film / Buccal	Titration phase: 150–900 μg every 12 h/dose increased depending on tolerability and analgesic need, Long-term treatment phase: 150–900 μg every 12 h / 12 months	Moderate-to-severe chronic pain	Open label single-arm study / ≤6week titration period and ≤48 weeks long term treatment period	Titration phase: n = 506 (roll over patients + de novo patients), Long term Treatment: n = 435 entered and n = 158 completed	Pain intensity, AEs	Belbuca™ shows efficacy and tolerability; pain intensity score remained constant during long-term management, and common AEs were nausea, headache, and constipation. *Note: Rollover patients who had completed the 12-week double-blind phase of the studies as opioid-naïve* (Rauck et al., 2016) *and opioid-experienced patients* (Gimbel et al., 2016)	Hale et al. (2017)
Besivance™ (Besifloxacin hydrochloride)	Vehicle as placebo	Ophthalmic suspension / Ophthalmic	0.6% suspension / Three times a day for 7 days	Acute bacterial conjunctivitis	Multicenter, randomize, double-masked, vehicle-controlled, parallel-group trial / 12 days	n = 518, randomization was in 2:1 ratio	Type and incidence of AEs observed at each visit	Besivance™ is safe in 1-year-old and older patients; no serious AEs reported; one TEAEs appear was dysgeusia	Malhotra et al. (2013)
Crinone® (Progesterone)	Utrogestan® n = 58	Gel / Vaginal	8% Progesterone 90 mg / one dose of vaginal gel daily	Luteal support	Prospective randomized Controlled Trial / Started 2 days after oocyte retrieval and in case of positive human chorionic gonadotropin continued up to the 10^th^ week of pregnancy.	n = 111 women Utrogestan®; n = 58, and n = 53 in Crinone® 8%	Changes in vaginal microflora, safety, signs of vaginal inflammation, evaluation of tolerance by questionnaires	In terms of safety and efficiency, there was no significant difference between two formulations, but Crinone is well tolerated and comfortable for patients, and it is an optimal choice for luteal support	Michnova et al. (2017)
Lialda® (Mesalamine)	Asacol (delayed-release Mesalamine (twice-daily, total dose 1.6 g/day)	Tablet / Oral	2.4 g (2 × 1.2 g) / Once daily dosing	Maintenance of endoscopic remission (mucosal healing) of Ulcerative Colitis (UC)	Multicenter, randomized, double-blind, active-control trial / 6-months (April 2005 to September 2009)	n = 826 (n = 415 in Lialda group and n = 411 in delayed-release mesalamine group)	Maintenance of endoscopic remission	MMX mesalamine 2.4 g/day (Lialda) is well-tolerated and non-inferior to delayed-release mesalamine 1.6 g/day for maintenance of endoscopic remission	D'Haens et al. (2012)
Suboxone® (Buprenorphine hydrochloride; naloxone hydrochloride)	Vivitrol (Extended-release naltrexone (XR-NTX), IM injection (4 mL, about 380 mg naltrexone base) after every 28 days	Film / Sublingual	4 mg/1 mg and 8 mg/2 mg / Daily doses of 8-24 mg	Opioid relapse prevention	Multicenter, Open-label, randomized controlled trial / 24 week (Jan 30, 2014 to May 25, 2016)	n = 570 (In XR-NTX group: n = 283, and in BUP-NX group: n = 287)	Opioid relapse-free survival	It is easier to initiate patients to BUP-NX than XR-NTX, positively affecting overall relapse; treatment is less effective with XR-NTX, but once initiated, both were equally effective and safe	Lee et al. (2018)
Zubsolv® (Buprenorphine hydrochloride; naloxone hydrochloride	Generic buprenorphine (8–16 mg daily)	Tablet / Sublingual	5.7/1.4–17.1/4.2 mg, once daily, titrated.	Opioid dependence	Prospective, randomized, active-controlled trial / June 2013 to January 2014	n = 313	COWS, SOWS, and VAS	Zubsolv® is well-tolerated, safe, and effective, showing improvement in reducing cravings, and withdrawal symptoms, and comparable efficacy with generic buprenorphine	Webster et al. (2016)
Zubsolv® (Buprenorphine hydrochloride; naloxone hydrochloride dihydrate)	Conventional sublingual tablet Suboxone®, Cohort 1 (high dose): receive 16/4 mg. Cohort 2 (low dose), receive 4/1 mg, once daily	Rapidly dissolving Tablet / Sublingual	Cohort 1 (high dose): receive 11.4/2.9 mg Cohort 2 (low dose), receive 2.9/0.71 mg once daily	Opioid dependence	Fasting, open-label, two-period, crossover comparative bioavailability study / From 23 February to 7 June 2016	Cohort 1 (n = 64), Cohort 2 (n = 61)	Pharmacokinetic assessment, pharmaceutical assessment, and safety assessment	Zubsolv® shows improved absorption than conventional tablet, with improved taste and decreased dissolve time, make it priority over conventional one.	Jönsson et al. (2018)
Metrogel® (Metronidazole)	Metronidazole tablet (500 mg, orally, twice daily for 7 days	Gel / Vaginal	One applicator twice daily for 5 days	Bacterial vaginosis	Randomized clinical trial / 6 months	Total n = 60, Randomization metrogel group (n = 27), comparator (n = 28)	Safety, efficacy, AE, Cost effectiveness	Both were safe and efficacious, n = 3, experienced nausea with comparator which was cost effective, but patients experiencing side effects with comparator should use metrogel.	Ransom et al. (1999)
Metrogel® (Metronidazole)	Placebo (metronidazole vehicle)	Gel / Topical	0.75% metronidazole gel twice daily + Oral Tetracycline	Rosacea	Multicenter, randomize, double-blind, vehicle-controlled, parallel-group trial / 6 months	Total n = 113, Randomization n = 44 in each group	Count of pustules, Inflammatory papules, measure of relapse, roughness, and scaling	Pustules, and papules eliminated in 59% subjects, 73% shows less erythema, decreased recurrence, increased disease free interval	Dahl et al. (1998)
Xaciato® (Clindamycin phosphate)	Hydroxy ethyl cellulose placebo gel	Gel / Vaginal	2% clindamycin	Bacterial vaginosis	Double-blind, placebo-controlled, randomized study / 30 days	Total n = 513, Randomization: Xaciato® group (n = 204), placebo gel (n = 103)	Safety, efficacy, and acceptability	Highly acceptable and shows rapid improvement in symptoms	Mauck et al. (2023)
MuGard™ (Carbomer-based mucoadhesive hydrogel) Medical Device	Sham control (SC) (flavored saline bicarbonate Rinse)	Mucoadhesive oral rinse / Oral cavity	Rinse with 5 mL for a full minute 4 times per day / 4 weeks	OM	Double blind, placebo-controlled, 2-arm parallel Study / Almost 2-3 months	Total n = 120, Randomization: MuGard™ group (n = 37), SC group (n = 41)	Mouth and Throat Soreness (MTS), subjective measurement of OM severity	Both groups show compliance; none of patient discontinue due AE of MuGard™, nausea and vomiting were noted in SC group, MuGard™ superiority for mitigating, delaying OM progression, OM symptoms, showed tolerability, and safety	Allison et al. (2014)
Episil® (Lipid-based self-assembling film) Medical Device	Outcomes measured before and after the application of Episil®	Oromucosal liquid (forms in-situ bioadhesive gel)	Apply few drops of solution on affected area, spread through tongue, leave it, until film form / 7 Days	For treating OM in patients with hematologic malignancies	Observational comparative study / From May 2018 to March 2019	Study 1; n = 37, (Patients who agreed to complete detailed questionnaire were included in study 2) Study 2; n = 22	OM severity by CTCAE, oral Function improvements regarding speech, pain relief, swallowing	In 19% of patients OM severity reduced, showed minimal AEs i.e., only 3 patients reported sticking and stinging problems, 84% patients showed their satisfaction with it by rating it ‘good’ and ‘very good’, showed improvement in speech, pain relief, swallowing, overall Episil® is a promising treatment for OM, especially in those where steroid ointment is contraindicated	Fukutani et al. (2024)

Furthermore, some of the important mucoadhesive delivery patents, thereby showing their potential for clinical utility and translation perspectives. Table 6 illustrates the different patent studies that were prioritized based on three essential criteria: (1) published within the time frame of the last 10 years, (2) clearly using “mucoadhesive” or equivalent in the claim/abstract, and (3) having the current status of published/granted/active. The prime purpose of MDDS is to target the mucosal surface, promote residence time of the delivery system, and positively influence drug release and bioavailability. Furthermore, the designed table highlights the key components, with particular emphasis on the incorporation of mucoadhesive polymers and other essential excipients required to achieve the mucoadhesive properties of the formulation. Additionally, the outcomes of the cited patents underscore the potential of MDDS to enhance patient compliance through improved therapeutic efficacy.

**Table 6:** Summarizing mucoadhesive delivery patents for underscoring their potential for patient benefits and translational value.

Patent Title	Patent No./Application No.	Jurisdiction	Publishing Year	Formulation/Delivery System	Key Components for Formulation Design	Claimed Benefits (Study Outcomes)	Status
Pharmaceutical formulation	US12285521B2	United States	2025	Oral transmucosal-film delivery system	Alginate salt of a monovalent cation (Na-alginate), plasticizers (PEG, glycerol, sorbitol, xylitol)	－ Remarkable increase in plasma drug concentration via oral mucosa avoid hepatic first-pass metabolism and improved drug's bioavailability. － Films were good in elasticity and mechanical strength.	Active^b^
Orally dissolving mucoadhesive films in managing oral care	EP3481428B1	European Patent Office	2021	Oral Dissolvable Mucoadhesive Film	Indigestible hydrophilic polymers (xanthan gum, CMC and HPMC), Plasticizers (Propylene Glycol and PEG 400), and carbopol	－ Controlled and continuous drug release without mastication or chewing of film made the system more effective for the treatment, including halitosis, dry mouth (xerostomia), gingivitis, denture sores, and sore throat. － The films were strong, elastic, and demonstrated a smooth surface to reduce friction between denture and gums.	Granted^a^
Lipid-based ophthalmic emulsion	US10925892B2	United States	2021	Mucoadhesive Lipid-based ophthalmic emulsion (o/w)	Mucoadhesive polymer (guar and hydroxypropyl guar), Surfactants (polyoxyethylene-40-stearate & sorbitan tristearate)	－ Increased polymeric concentration delivered improved ocular protection, retention, and film stability that exhibited enhanced efficacy in contrast to US10004685B2. － Emulsion was less opaque and reduced blurring effect upon instillation.	Active
Mucoadhesive polymeric drug delivery compositions and methods	WO2021155477A1	WIPO (International)	2021	Mucoadhesive Polymeric Paste (Injectable, Low-Viscosity)	PEG 300, PEG 1000, PLGA, Hyaluronic Acid	－ Exhibited a promising strategy for UTC treatment because of extended localized exposure, controlled release, and prolonged systemic presence of drug.	Published
Mucoadhesive dispersion nanoparticle system and method for production the same	WO2020053609A1	WIPO (International)	2020	Mucoadhesive Dispersion Nanoparticle System (SLNs)	HPMC, Carnauba wax (solid lipid), Red palm oil (liquid lipid), TPGS and Polysorbate 40 (surface active agents)	－ Increased drug retention time by 87% generating prolonged therapeutic effects. － Decrease dose frequency by 40-80 times. － SLNs penetrate and show erosion intracellularly to release drug.	Published
A mucoadhesive oromucosal formulation comprising a nicotine complex	WO2018113916A1	WIPO (PCT)	2018	Mucoadhesive Oromucosal Formulation (Liquid mouth spray, oromucosal gel, solid formulation, or lozenge)	Mucoadhesive water-soluble anionic polymers can be: xanthan gum, carrageenan or carbomer(s) (e.g., Carbopol 974 G, Carbopol 71 G)	－ Prolonged intraoral residence time and controlled drug release was monitored. － Decreased wash off of free drug towards GIT helped to reduced side effects.	Published
Ophthalmic emulsion	US10004685B2	United States	2018	Mucoadhesive ophthalmic emulsion	Mucoadhesive polymer (galactomannan polymer, i.e., hydroxypropyl guar), Mineral Oil, Hydrophilic Surfactant (Polyoxyethylene-40-stearate), Hydrophobic Surfactant (Sorbitan tristearate), Stabilizing agents (DMPG)	－ Incorporation of mucoadhesive helps to enhance the residence time and maintaining the emulsion on ocular surface. － Targeted delivery of lipophilic compounds facilitate lubrication and tear film stabilization. － Suitable to use as dry eye therapeutics.	Active
Mucoadhesive nanoparticle composition comprising immunosuppressant and methods of use thereof	US9878000B2	United States	2018	Mucoadhesive Nanoparticle Delivery System (Aqueous suspension)	Amphiphilic block copolymers: Hydrophobic block (polylactide), Hydrophilic block (Dextran) and Phenylboronic acid	－ Prolonged retention at ocular mucosa helped to reduce dose frequency. － Enhanced hydration of the mucous membrane facilitated to preserve or restoring the function and integrity of the mucosal lining.	Active
Muco-adhesive, controlled release formulations of levodopa and/or esters of levodopa and uses thereof	US10098845B2	United States	2018	Oral solid dosage form (dual-component system)	Rate-controlling polymer (Cellulose Acetate), Muco-adhesive polymer (Eudragit® E100 or CS), Enteric coating polymer (Methacrylic Acid Copolymers like Eudragit® L100-55)	－ Rapid increase in plasma drug concentration and extend for a prolonged period of time. － Enteric coating helped to prevent the drug from the stomach's degradable environment and facilitate it release into the small intestine. － Improved retention assisted to control the drug release and facilitated the enhanced drug absorption.	Active
MBT for the treatment of orofacial herpes	EP2509586B1	European Patent Office	2018	MBT	Bioadhesive polymer (Milk protein concentrate), Sustained release agent (HPMC), Tablet binder (PVP)	－ Well tolerated, safe and sustained mucoadhesive tablets were achieved. － Rapid and sustained drug release was monitored that promoted better healing and reduced infection spread and recurrence.	Active
Mucoadhesive buccal in situ gel formulation	EP3173067A1	European Patent Office	2018	Mucoadhesive Buccal In Situ Gel Formulation	Mucoadhesive Polymers (Carbopol 934), Surface Active Agent (Tween 80, Cremophor RH 40, or ascorbic palmitate)	－ Enhanced retention at oral mucosal surface and a controlled drug release profile facilitated to eliminate the pain, irritation, and inflammation effectively.	Active
Mucoadhesive patch with opposite ratios of nonionic and anionic hydrocolloids in adhesive and backing layer	US9320721B2	United States	2016	Mucoadhesive Patch	**Nonionic Hydrocolloids:** Ethyl cellulose and HPC, **Anionic Hydrocolloids:** Carbopol 971 and Gum arabic	－ Firm attachment of patch to mucosal surface and rapid *in vitro* drug release (91% after 1 h) were observed. － 77% of subjects found the patch easy to use (avg. adhesion time was 5.6 h), and 62.5% judged the adhesion as good, and the taste was neutral. － Patches were designed for the treatment of gingivitis, periodontitis, herpes labialis, and herpes genitalis and could deliver the antiseptic, antimicrobial, and local anesthetic responses effectively.	Granted (Expired - Fee Related)
Sublingual apomorphine	US9326981B2	United States	2016	Mucoadhesive films or strips	Mucoadhesive polymers (alginates, carrageenan, Xanthan gum, polyacrylate, or carboxymethylcellulose; or pullulan, pectin, locustbean gum, Na-alginate, gum Arabic	－ Safety and efficacy of apomorphine therapy have been improved. － Drug penetrate the mucosal tissue and enhances bioavailability. － Delivered rapid onset of action for use a rescues therapy in Parkinson's disease.	Active

WIPO: World Intellectual Property Organization, PCT: Patent Cooperation Treaty, PEG: Polyethylene glycol, PLGA: Poly (lactic-co-glycolic acid), UTC: Upper Tract Urothelial Carcinoma, SLNs: Solid Lipid Nanoparticles, HPMC: Hydroxypropyl Methyl Cellulose, TPGS: Tocopheryl polyethylene glycol 1000 succinate, HPC: Hydroxypropyl cellulose, DMPG: Dimyristoyl phosphatidylglycerol, CMC: Carboxymethyl cellulose, GIT: Gastrointestinal tract, PVP: Polyvinylpyrolidone.

a Granted = Approved and issued.

b Active = Granted and currently valid (not expired, not abandoned, and fees are up to date).

Distinct studies (patents) exhibit clinical benefits, such as improved drug bioavailability, targeted therapy, reduced dosing frequency, and the translational potential of mucoadhesive delivery systems. These delivery systems can be adopted for a wide range of active ingredients, making them attractive for the pharmaceutical sector. Active/granted patents explicitly explain the marketed potential of MDDS. These technologies are remarkably beneficial because of numerous advantages: (1) available in different forms like buccal films, transdermal patches, mucoadhesive tablets, gels, and solutions (Kulkarni et al., 2023), (2) providing multiple patient-oriented benefits, like prolonged retention time, controlled drug release, improved drug absorption and bioavailability, reduced dosing frequency, minimized side effects, and improved patient compliance (Bácskay et al., 2025), that directly demonstrate their translation value.

### 6.2 Industry strategies for formulation development

At the industrial level, the manufacturer must consider multiple perspectives in the manufacturing of commercial products, in which the bioavailability of the drug product is considered to be the most important one (Stielow et al., 2023). In addition to bioavailability, the developer must also account for the stability and tolerability of the final drug substance (Stielow et al., 2023; Chow, 2014). Therefore, transitioning mucoadhesive products from laboratory-scale into industrial-scale development requires detailed formulation planning (Reddy Palem et al., 2024). Industrial strategic development of these products requires a balance between large-scale manufacturing and scientific innovations on a small-scale (Federsel, 2009). Their formulation planning depends on the polymer-excipient optimization, drug-polymer compatibility, platform technology approaches, scalable manufacturing methods, patient acceptability, and cost-effectiveness (Sangavi et al., 2025; Ricarte et al., 2019; Ahuja et al., 2010).

From an industrial formulation development perspective, fabrication strategy is an important component. It can directly influence the performance-related factors, i.e., polymer distribution, drug content uniformity, mechanical properties, mucoadhesion, and drug-release behavior. Traditional techniques like solvent casting for mucoadhesive films are widely used due to their simplicity and flexibility (Hassan et al., 2023). The hot-melt extrusion can provide more continuous manufacturing, but careful control of thermal and shear stresses is required for thermolabile polymers or drugs (Shipp et al., 2022). Emerging strategies like electrospinning offer additional control on drug release behavior; however, process parameter optimization and QbD strategies are required for translational purposes (Hassan et al., 2023).

#### 6.2.1 Polymer-excipient optimization

During industrial development, after selecting a drug molecule with its appropriate MDDS, the industrial formulators focus on selecting the appropriate polymer with certain characteristics, such as having mucoadhesive strength, site specificity, a safety profile, and the swelling behavior of the polymer, to increase the tolerability, safety, and bioavailability of the mucoadhesive product (Ali et al., 2006; Desai et al., 2025). Moreover, it should be non-toxic, non-irritant, and cost-effective and should have a strong covalent bond with mucin (Jawadi et al., 2022; Kavitha et al., 2011).

To improve the therapeutic efficacy, mucoadhesion, and residence time of marketed drug products, several mucoadhesive bases and technologies have been utilized, in which polymers play an important role to make the strong foundation for mucoadhesion as presented in Table 4. These may include natural (i.e., CS, alginate, xanthan gum), synthetic (i.e., polycarbophil, polyethylene oxide), and semisynthetic polymers (i.e., HPMC, HEC), used for their mucoadhesive properties and potential to contribution to controlled release. The selected polymers in marketed products represent a perfect balance between efficacy, safety, and formulation requirements, ensuring their importance in the clinical performance of MDDS (Kim et al., 2017; Amol et al., 2024). However, to achieve polymer optimization, formulators are working on the use of a combination of polymers to attain dual purposes, i.e., controlled release of the drug and mucoadhesion (Crispino et al., 2025). For example, polycarbophil is used in DuraSite technology for delivering azithromycin, where the polymer not only gives mucoadhesion and increases the ocular residence time of the drug but also provides stability to the formulation (Sahin et al., 2025).

In addition to selecting the suitable polymer, the excipients are also commonly selected according to the requirements of particular DDS (Arribada et al., 2025). In the selection of these, it should be carefully evaluated that, in addition to fulfilling all the primary requirements of the drug, the selected one should not affect the physical as well as the therapeutic efficacy of the active drug. In pursuit of this, the FDA provides the Inactive Ingredient Database, which lists those excipients that have been used in products that have already been approved by the FDA. These excipients can be used for the initial development of pharmaceuticals. Once the excipient for a particular route of administration in a specific dosage form has appeared in an approved pharmaceutical, it will not require a detailed review the next time to be included in the development of a new drug product. The applicant could consider this safe for the new drug product with the same route and dosage form. So, the information on excipients used in this database can be used as an aid for industries in the development of drug products (FDA, 2019a).

#### 6.2.2 Drug–polymer compatibility and stability

Compatibility between drug and polymer is another strategy to ensure that the presence of polymer should not affect the physical and therapeutic efficacy of the drug (Liu et al., 2004). To check the long-term drug-polymer compatibility, the pharma industries deploy preformulation screening with X-ray diffraction, differential scanning calorimetry, and stress testing (Tiwari et al., 2025; Kandarapu et al., 2001). Stress testing includes isothermal stress testing, which holds importance in the pharmaceutical industry, in which a mixture of drug and excipient is stored at high temperature with or without moisture for a particular duration. Then drug content is determined by high-performance liquid chromatography (Kandarapu et al., 2001). These techniques used to check the long-term compatibility often hold importance for biologically labile active ingredients such as nucleic acids, peptides, and proteins. Different coated and layered designs, such as single-layer film coating and bilayer tablet formulation, are introduced to overcome the drug-polymer interactions while sustaining the mucoadhesion of the polymer (Nguyen et al., 2019; Mohana Raghava Srivalli et al., 2013).

#### 6.2.3 Platform technology approaches

Innovation in drug delivery technologies is an influential strategy that pharma companies employ to excel over their competitors (Wentz et al., 2019; Kandasamy, 2024). Many of them are utilizing these advanced drug delivery technologies, e.g., DuraSite®, PharmFilm®, Lauriad® and MMX® technology, to improve therapeutic response and patient compliance. These have been utilized to ensure targeted and prompt delivery of active drug products as shown in Table 4.

***DuraSite®,*** a polycarbophil-based ophthalmic DDS, was first designed by InSite Vision, and after that it was developed by Sun Pharmaceuticals (Wong et al., 2021; Hosseini et al., 2016). It forms a mucoadhesive polymeric gel matrix, which prolongs the residence time of the drug on the eye surface, which improves the therapeutic efficacy and bioavailability of the drugs used in ophthalmic treatments (Singh et al., 2020; Shafiee et al., 2013a). FDA-approved drug products that utilize this technology are AzaSite®, BromSite®, and Besivance™ (FDA. AZASITE, 2024; FDA. BESIVANCE, 2018; FDA. Bromsite, 2023).

***PharmFilm®,*** a rapidly dissolving oral thin film DDS*,* is based on film-forming, mucoadhesive polymers, i.e., HPMC, PVA, and pullulan (Singh et al., 2017). When applied, it sticks to the oral or buccal mucosal surface, gets hydrated, and releases the drug, which is rapidly absorbed, accelerates the onset of action, and bypasses the first-pass metabolism. Additionally, it holds the characteristics of uniform drug distribution, dose accuracy, and convenience, which make it suitable to be used by geriatric and pediatric patients (Therapeutics, 2024). The commercial success of this technology is demonstrated by the mucoadhesive marketed drug products such as Suboxone® and Sympazan® to improve drug delivery and patient compliance (Mundhey et al., 2016; FDA. SYMPAZAN, 2024).

***Lauriad®,*** a mucoadhesive buccal targeted DDS, was developed by BioAlliance Pharma (Desai et al., 2025). It was designed to prolong the residence time of the drug, supporting local as well as systemic drug delivery through buccal mucosa (Mauceri et al., 2025). It is based on HPMC, a mucoadhesive polymer, which enables it to stick to the buccal or gingival mucosa and get hydrated, facilitating prolonged drug release. A marketed drug product that utilizes this technology is Oravig™, used for the treatment of oropharyngeal candidiasis, improving patient compliance (FDA. ORAVIG, 2020; J Tangso et al., 2015).

***MMX® technology*** is an oral colon-targeted DDS specifically designed to release the drug in the large intestine (Yeung et al., 2025). It was originally developed by Cosmo Pharmaceuticals for making the oral formulation of mesalamine, i.e., Lialda®, used for the treatment of ulcerative colitis (D'Haens et al., 2012; Gvozdeva et al., 2025). It is formulated as a tablet that has a core containing the active ingredient, which is then surrounded by the double matrix system consisting of hydrophilic and lipophilic excipients, enclosed within pH-dependent, gastro-resistant coatings (Nardelli et al., 2017). In this double matrix system, the lipophilic matrix is dispersed within the hydrophilic network, and the active ingredient is incorporated within the microparticles of the lipophilic matrix. The gastro-resistant coating helps the formulation to remain intact in the stomach. However, when the tablet reaches the terminal ileum, the coating disintegrates, the hydrophilic polymer swells, and the tablet turns into a gel mass. The aqueous fluid slowly penetrates the core due to the lipophilic matrix. The drug starts to diffuse from the lipophilic matrix and slowly covers the whole colonic mucosa. In this matrix system, the exposure of the drug is prolonged and homogeneous, delivering the drug at the target site in a therapeutic amount (Sriram et al., 2021; Chen, 2020).

Most of the firms go for these technologies because they can be employed for various therapeutic agents, instead of developing the product from scratch. Moreover, these approaches provide regulatory insights, reduce the development time, and allow lifecycle strategies such as reformulations or adding new therapeutic uses of drug products (Kandasamy, 2024).

### 6.3 Design thinking and human factors in product development

Design thinking and human factors are crucial attributes to be considered during the commercialization of MDDS in addition to maintaining the therapeutic efficacy of the product (Menditto et al., 2020). As in the product development process, the stability and pharmacokinetics are prioritized; patient usability and acceptance should be important considerations in a similar manner (Religioni et al., 2025; Xiong et al., 2025).

Patient acceptability is a distinguishing human factor that depends on various factors such as administration schedule, ease of use, texture or feel, tolerability, irritation, patient comfort, smell, and the taste-masking effect of the product during the development of either a conventional DDS or MDDS (Menditto et al., 2020; Religioni et al., 2025; Xiong et al., 2025). Certain additional factors are also considered for MDDS in particular, such as the mucoadhesion strength, salivary milieu, disintegration time, release profile, thickness of film, and speech interference (Jovanović et al., 2021). These should be the key points to be considered for designing Mucoadhesive Oral Gels (MOG), tablets, films, and patches to ensure sustained release and comfort, as they directly interact with the mucosal surface, influencing patient compliance and therapeutic outcomes (de Carvalho et al., 2023; Boddupalli et al., 2010).

To reduce the dosing frequency, mucoadhesive products are developed, which leads to improved compliance. Integration of design thinking is meant to consider the lifestyle of the patient, such as their working environment, meal time, and mobility, so that products could be developed with a reduced dosing schedule and minimal disruption (Sangavi et al., 2025). A comparison of buprenorphine-naloxone film and buprenorphine-naloxone tablets was performed among 90 patients in a randomized controlled trial over the treatment period of 31 days. The film showed reduced dosing frequency compared to tablets. Ratings for these two products regarding convenience, ease of use, and taste were determined. Among 90 patients, 52 prioritized the film, and 20 reported interest in tablets, while 14 reported interest in either film or tablet. Although films exhibited rapid dissolution and were preferred over the tablets, the problems regarding their use reported by the patients were difficulty in picking up the film and film getting stuck to wet fingers, the roof of the mouth, teeth, and/or cheeks. Moreover, placement of the film was done under the supervision of clinicians to avoid overlapping, as incorrect placement could lead to reduced mucosal adherence (Lintzeris et al., 2013). Although the mucoadhesive products have advantages, at the same time human factors could compromise their potential.

Special populations, i.e., the elderly and children, should also be involved in design thinking testing rather than extrapolating the data from healthy volunteers, as they may often face problems regarding the use of these products, such as swallowing a dosage form that sticks to the wall of the esophagus, causing discomfort (Dias et al., 2022). In elderly patients, reduced saliva production and dexterity, while in children, film flexibility and taste pose unique challenges (Menditto et al., 2020). Patient preference for a particular dosage form could be assessed by comparative studies. For example, a comparative clinical trial was conducted to compare the MBT with MOG for clinical efficacy. MBT (50 mg) was found to be superior to MOG (500 mg) for the treatment of oropharyngeal candidiasis due to reduced dosing frequency, strong adhesion, and good compliance and tolerability even in xerostomic patients. No adverse events were reported in patients with MBT, but a few patients showed discomfort, bad taste, burning sensation, and pain (Bensadoun et al., 2008). In another trial, two marketed mucoadhesive products named Suboxone® (sublingual film) and Zubsolv® (sublingual tablet) were compared. Zubsolv® shows rapid absorption and is prioritized over Suboxone® due to decreased dissolution time and improved taste (Jönsson et al., 2018). In this way, maximum therapeutic potential could be achieved for the mucoadhesive products by considering certain factors during their development; among these are retention at the target site, palatability, burning sensation, and oral comfort (Patel et al., 2011).

Mucoadhesive strength and duration are the key elements that influence the human factors involved in the product's effectiveness. Weak mucoadhesive strength causes the early detachment of the drug from the mucosal surface and could lead to dosing failure, while excessive mucoadhesive strength may cause mucosal irritation and discomfort, which results in reduced therapeutic effectiveness and may cause the patient to show non-compliant behavior (Jovanović et al., 2021). Moreover, the FDA often highlights human factor engineering studies to ensure that packaging, labeling, and instructions for the users minimize errors, which is particularly useful for self-administered mucoadhesive medical devices (FDA, 2016).

Design thinking demands that **t**here be a need to balance patient usability (i.e., absence of pain on removal and ease of removal of patches) with pharmacokinetic needs such as long residence time for controlled release of product (Menditto et al., 2020; Radu et al., 2025). These findings suggested that clinical efficacy cannot be separated from patient usability and that real-world outcomes are strongly influenced by the human factors such as acceptability, ease of use, and comfort, which are the central points for design optimization.

### 6.4 Regulatory pathways and challenges

The EMA and FDA are the two regulatory agencies that ensure the efficacy, safety, and quality of medical devices and pharmaceutical products before giving their approval for commercialization (Makrani et al., 2025). These agencies provide particular pathways for approval depending on their classification, such as whether they are classified as drugs, devices, or combination products (FDA, 2018; FDA, 2022; FDA. Development and Approval Process, 2022)

#### 6.4.1 FDA regulatory pathway

In the United States, drugs are tested prior to their sale by the manufacturer. To assess safety, animal studies are performed first; after that, a series of tests are conducted in humans to determine whether the drug is safe enough to treat the particular disease or whether it provides the intended health outcome. The company then sends the test reports to the Center for Drug Evaluation and Research (CDER), a division of the FDA, to demonstrate the drug's efficacy and safety for a specific use. The team at CDER then reviews the report; if it is evident from the reports that the proposed drug has more benefits than the risks, then it gets approval for sale (FDA. Development and Approval Process, 2022). Examples of FDA-approved/cleared mucoadhesive products, including drug products and medical devices, are summarized in Table 4.

To be regulated by the FDA, primarily mucoadhesive products should be classified into three classes, such as (1) pharmaceutical drugs that contain API, e.g., Crinone®; (2) combination products that incorporate the drug with a particular delivery system, i.e., DuraSite®; and (3) medical devices that do not contain the API, e.g., MuGard™ used as a mucoadhesive oral rinse (FDA, 2013; FDA. AZASITE, 2024; FDA, 2006). The FDA approves medical devices through premarket approval or 510(k) premarket notification, while drugs are evaluated through Abbreviated New Drug Applications (ANDAs) under section 505(j) or New Drug Applications (NDAs), including applications under section 505(b)(1) and, where applicable, 505(b)(2) (FDA. Development and Approval Process, 2022; FDA, 2024).

The FDA 505(b)(2) application may provide a potential regulatory pathway for MDDS containing an established API but incorporating a novel delivery technology or modified dosage form. The 505 (b)(2) pathway is an NDA route in which the application relies, in whole or part, on studies published in the scientific literature or on the information from previously approved drug products. The applicability of this pathway depends on the specific dosage form, formulation, route of administration, and degree of modification (FDA and U, 2020).

Combination products, i.e., DuraSite®, Lauriad®, and PharmFilm®, which may contain a variety of components, are regulated according to their primary mode of action and constituent components. The FDA's office of combination products coordinates the regulatory oversight of combination products and determines the appropriate lead center, which may include CDER, the Center for Biologics Evaluation and Research (CBER), or the Center for Devices and Radiological Health (CDRH), with the relevant center leading the product's regulatory review as appropriate (FDA, 2019b).

For the approval of medical devices, the FDA evaluates eligible devices through the 510(k) pathway, which requires the manufacturer to explain that the device is substantially equivalent to a legally marketed predicate device, which was marketed legally before the 28th of May, 1976 (FDA, 2024; FDA, 2023b). MuGard™, Episil®, Gelclair®, and Gengigel® are mucoadhesive medical devices approved through the 510(k) pathway (FDA, 2001; FDA, 2006; FDAa; FDA. EPISIL 510, 2011).

#### 6.4.2 EMA regulatory pathway

In the European Union (EU), drugs are approved through centralized procedures, which apply particularly to innovative products, and through mutual recognition/decentralized procedures for regionally marketed and generic medicines (Gjeorgiev et al., 2025; Agency, 2025). EMA demands comprehensive data to check the safety, efficacy, and quality of the product (Ahmed, 2024). Sometimes the EMA requires local tolerability studies, particularly when the product is in direct contact with a mucosal surface, such as mucoadhesive sublingual or buccal films, which may require additional studies as compared to mucoadhesive oral tablets (Agency, 2015). Mucoadhesive products containing the API, such as Metrogel®, AzaSite®, and Lialda®, are regulated in the EU as medicinal products by established EMA regulatory pathways.

Medical devices are governed by the Medical Devices Regulation (EU) 2017/745, which requires conformity assessment of medical devices with safety and efficacy standards; if they meet the standards, then the devices get the CE marking (Agency, 2017, 2025). Mucoadhesive devices such as wound dressings or oral protective gels that do not contain the API are categorized as either class IIa or IIb devices, based on their risk profile.

#### 6.4.3 Regulatory challenges

There is an overlap between medicinal products and devices in regulatory classification, which poses a challenge to the EMA (Reis et al., 2022b). It can be clarified that the Suboxone® (i.e., sublingual/buccal film) sticks to the mucosa and may appear as a medical device at first glance due to its dosage form. But its therapeutic effect is due to the API, i.e., buprenorphine hydrochloride and naloxone hydrochloride, contained in it. Therefore, Suboxone® is regulated as a medicinal product by the EMA, despite its device-like dosage form (FDA. SUBOXONE, 2025). Although in the FDA approval process, there is a defined classification of mucoadhesive products as a drug, combination product, and medical device. But sometimes, in the case of combination products, it is required for both the drug and drug delivery technology, i.e., DuraSite®, to meet the regulatory standards, which complicates the regulatory approval process. Because to check the safety and efficacy, the clinical data of drug delivery technologies is also required by these regulatory agencies. These can cause ambiguity during early development and impact the requirements, approval process, and timelines of the regulatory pathway (Vigani et al., 2023).

The introduction of novel technologies used in MDDS further complicates the regulatory process, as these may require extensive stability studies, safety testing, and manufacturing validation at a large-scale (Polaka et al., 2022; Price et al., 2014). The high cost of these studies often makes it impractical to introduce innovations or new polymeric materials in the formulation. As a result, small companies or academic researchers often rely on regulatory-approved and well-established polymers for commercially viable technologies. Although it speeds up the regulatory process, the use of effective and novel biomaterials is discouraged (Price et al., 2014).

## 7 Case studies from industry

### 7.1 Success stories of commercial mucoadhesive products

Numerous successful commercial products have been delivered by mucoadhesive platforms across oral (Petrakis et al., 2025; Jones et al., 2023; Rauck et al., 2016; Gimbel et al., 2016; Hale et al., 2017; D'Haens et al., 2012; Lee et al., 2018; Webster et al., 2016; Jönsson et al., 2018; Allison et al., 2014; Fukutani et al., 2024), ocular (Abelson et al., 2007, 2008; Luchs, 2008; Malhotra et al., 2013), topical (Dahl et al., 1998), and vaginal (Michnova et al., 2017; Ransom et al., 1999; Mauck et al., 2023) routes after solving the clinical and patient usability problems. Other routes, including rectal and pulmonary delivery, have comparatively fewer successful marketed mucoadhesive products due to certain physiological and formulation challenges, but they remain important areas for development (Salatin et al., 2022; Kumar et al., 2024; de Souza Carvalho et al., 2014).

The notable successes of AzaSite® (FDA. AZASITE, 2024), Besivance™ (FDA. BESIVANCE, 2018), Suboxone® (Mundhey et al., 2016; FDA. SUBOXONE, 2025), Belbuca™ (FDA. Belbuca, 2023), Oravig™ (FDA. ORAVIG, 2020; J Tangso et al., 2015), Episil® (FDA. EPISIL 510, 2011), and MuGard™ (FDA, 2006; Mugard, 2020) show how the selection of appropriate mucoadhesive technology with clinical benefits could help in the development of commercially successful products.

AzaSite®, an ophthalmic solution, utilizes the DuraSite® (polycarbophil-based) DDS, with prolonged residence time that improves the bioavailability of azithromycin on the ocular surface (Shafiee et al., 2013b). A randomized controlled phase III trial was conducted comparing the AzaSite® with tobramycin as a comparator for the treatment of bacterial conjunctivitis. Bacterial eradication rates reported were 88.1% and 94.3%, and clinical resolution was achieved in 79.9% and 78.3% of the participants for AzaSite® and tobramycin, respectively. While the efficacy of both formulations was the same, AzaSite® showed similar clinical outcomes with 65% less frequent dosing than tobramycin (Abelson et al., 2007). Besivance™ (Besifloxacin hydrochloride ophthalmic suspension) is another marketed mucoadhesive product that uses the DuraSite® platform to treat acute bacterial conjunctivitis (Malhotra et al., 2013; Shafiee et al., 2013b). A multicenter randomized controlled trial showed the efficacy of Besivance™ in patients aged 1 year and older, with no serious AEs reported; ocular TEAEs were reported in 4.9% of patients compared with 6.5% in the vehicle-treated group (Malhotra et al., 2013). The reports of these trials showed DuraSite® as a successful residence-extending platform (Abelson et al., 2007; Malhotra et al., 2013).

Suboxone® (Buprenorphine hydrochloride and naloxone hydrochloride buccal film), with improved palatability, another successful mucoadhesive product, uses PharmFilm® technology. In an open-label, randomized controlled trial, patients found the buccal film more convenient to administer than extended-release naltrexone administered by intramuscular injection (Vivitrol). Suboxone® exhibited positive outcomes for relapse prevention, with 57% of patients remaining relapse-free over 24 weeks, compared with 65% of patients receiving Vivitrol (Lee et al., 2018).

Belbuca™ (buprenorphine hydrochloride), a polycarbophil-based mucoadhesive buccal film, has shown its efficacy and safety in both opioid-naïve and opioid-experienced patients with moderate-to-severe chronic lower back pain (Rauck et al., 2016; Gimbel et al., 2016). During long-term treatment in opioid-naïve patients, Belbuca™, 36.3% of the patients completed 48 weeks, with 14.0% of treatment-related AEs. While 3.2% discontinued the treatment due to AEs. The dose of Belbuca™ remained unchanged in 86.2% of patients, supporting its tolerability and efficacy during long-term treatment (Rauck et al., 2016). In a randomized withdrawal study conducted in opioid-experienced patients, mean pain intensity decreased from 6.7 to 2.8 after the opioid titration period. The Belbuca™ group showed greater reduction in pain score than the placebo group at week 12 (difference of −0.98 [95% CI: −1.32, −0.64]; p < 0.001), with more patients achieving ≥30% and ≥50% pain reductions (P < 0.001 for both) (Gimbel et al., 2016).

Oravig™, an MBT that uses Lauriad® technology, is the topical application approved by the FDA, used for the treatment of OPC for many years (FDA. ORAVIG, 2020; J Tangso et al., 2015). It improves the contact time of the drug by adhering to the oral mucosa and providing the sustained release of the drug over several hours (Mauceri et al., 2025). It is generally well tolerated, safe, effective, user-friendly, and convenient as the first new topical application with minimal AEs such as headache, diarrhea, and nausea. Moreover, its reduced dosing frequency, i.e., once-daily dosing, helps to improve the patient's compliance, making it superior to the other formulations needing multiple daily doses (FDA. ORAVIG, 2020).

Moreover, Episil® and MuGard™ are successful mucoadhesive medical devices used for the treatment of OM and mucosal pain relief, respectively (FDA, 2006; FDA. EPISIL 510, 2011). MuGard™ (Carbomer-based mucoadhesive oral rinse) showed safety and a good tolerability profile when compared to SC (i.e., flavored saline bicarbonate rinse) in a double-blind, placebo-controlled trial. Patients receiving MuGard™ showed compliance. None of the patients discontinued due to its AEs. Nausea and vomiting were noted in the SC group, and MuGard™ showed superiority in mitigating and delaying OM progression and its symptoms (Allison et al., 2014). While the Episil®, a bioadhesive oromucosal liquid medical device, is used to protect the ulcerated area of the mucous membrane and helps to reduce the pain in patients with OM. To evaluate the efficacy of Episil® for treating OM in patients with hematologic malignancies, a comparative study was conducted by Fukutani et al. in which 84% of the patients rated their experience as ‘good’ and/or ‘very good’; however, in 19% of the patients, the severity of OM reduced. Episil proved to be a promising approach for improving speech, swallowing, eating soft foods, and QoL with minimal AEs (Fukutani et al., 2024).

Collectively, these studies show that the success of these mucoadhesive products and medical devices depends not only on their inherent mucoadhesive technology but also on synchronizing the formulation design with patient requirements, showing improved clinical advantages in well-structured clinical trials, and utilizing strong regulatory and industrial approaches for commercialization. By incorporating technological innovations with patient-oriented usability and collaborations, mucoadhesive systems have shifted from large-scale use to become long-term therapeutic solutions in commercial markets.

Beyond mucoadhesive products, established marketed products can provide useful benchmarks for formulation development of MDDS in the future. Mucinex® (guaifenesin), an orally administered extended-release tablet, is used for the treatment of productive cough due to upper respiratory tract infections (Tripathi et al., 2019). It's a bilayer tablet with immediate- and extended-release layers to provide symptomatic relief for up to 12 h with reduced dosing frequency (Mucinex, 2026). Although Mucinex® is not a mucoadhesive tablet, its successful marketing implementation explains an important principle that could be applicable to MDDS: product performance must be aligned well with a clinically relevant release profile and a simple dosing regimen rather than prolonged residence time of the drug alone. For future development of mucoadhesive systems, this principle highlights the need to balance residence time, drug-release profile, therapeutic effectiveness, patient acceptability, and producibility during formulation development (Administration, 2024).

### 7.2 Lessons learned from failed or withdrawn formulations

Formulation withdrawals and failures in the area of MDDS offer valuable insights. Sometimes the mucoadhesive technologies have strong potential, but their progress is stopped due to other serious dangers such as safety, commercial, and regulatory concerns, and potential misuse. Rather than enlisting individual product codes, the key lessons can be grouped under the recurring themes of lack of observable clinical outcomes, safety or abuse risks, regulatory challenges, and manufacturing scalability issues.

Recently, manufacturers declared the discontinuation of some formulations of Transmucosal Immediate Release Fentanyl (TIRF) for commercial, regulatory, and public health reasons. The FDA has released updates and guidance on Risk Evaluation and Mitigation Strategies (REMS) for TIRF formulations that demonstrate abuse and safety concerns. During the period 2024-2025, some manufacturers are required to stop production of various TIRF products. The TIRF product contains fentanyl as an active ingredient, which is a prescription opioid used to relieve transient pain. These products are used in cancer patients who are also routinely taking other opioid medicines around the clock for pain management. Since 2011, the only purpose for the establishment of REMS required by the FDA is that only opioid-tolerant patients were dispensed and prescribed these products. When it was observed that patients who were not opioid tolerant were also receiving these products, the REMS was modified in 2020; the program adds further strictness for pharmacies, patients, and prescribers to reduce inappropriate prescribing, overdose, and misuse while ensuring access (FDA. Transmucosal Immediate-Release Fentanyl and TIRF, 2024).

The failure and withdrawal of other medicines may often relate to no clear clinical outcomes as compared to the existing therapies. Clinicians or the payers will not adopt it if the mucoadhesive product reformulation does not produce a notable improvement in patient-centered clinical outcomes such as symptomatic relief, reduced dose frequency, and improved compliance (González-Bueno et al., 2018). Therefore, for the locally acting mucoadhesive products, the regulatory authorities ask for clinical outcome studies rather than depending solely on pharmacokinetic biomarkers (FDAb). If the clinical outcomes are uncertain, the complexity and the high cost of these trials can terminate the program (Bentley et al., 2019). Manufacturing scalability problems such as poor content uniformity, instability, high cost of materials, and variable adhesion also destroy the program and can make these products commercially impractical (Goh et al., 2024; Antony Jose et al., 2025). Moreover, withdrawal of the product due to certain commercial reasons, i.e., low sales, competitive behavior, and market strategy, is also common (Petrova, 2013).

Regardless of increasing clinical translation of MDDS through oral (Petrakis et al., 2025; Jones et al., 2023; Rauck et al., 2016; Gimbel et al., 2016; Hale et al., 2017; D'Haens et al., 2012; Lee et al., 2018; Webster et al., 2016; Jönsson et al., 2018; Allison et al., 2014; Fukutani et al., 2024), topical (Dahl et al., 1998), vaginal (Michnova et al., 2017; Ransom et al., 1999; Mauck et al., 2023), and ocular (Abelson et al., 2007, 2008; Luchs, 2008; Malhotra et al., 2013) routes, rectal delivery remains comparatively unexplored. Mucoadhesive rectal delivery systems may present opportunities for local treatment of inflammatory bowel disease by prolonging residence time of the formulation and increasing local drug delivery at the affected mucosa (Farahani et al., 2026). However, variable mucus turnover, discomfort, fecal clearance, rectal fluid volume, and lower patient compliance may limit reproducible retention and clinical performance (Ramadan et al., 2018; Hua, 2019). Further formulation and clinical research is therefore warranted to establish the translational potential of rectal MDDS for inflammatory bowel disease.

### 7.3 Academia–industry partnerships in mucoadhesive research

The collaborative work between industry stakeholders and academic innovators provides benefits for the commercialization of mucoadhesive technologies (Arvidsson et al., 2016). Industries contribute experience in regulatory submissions, carrying out clinical trials, and Good Manufacturing Practice (GMP) scale-up. This partnership has been critical to overcoming the gap between laboratory findings and successful commercialization of mucoadhesive products (Souto et al., 2020).

Licensing of the platform technologies has been a proven approach. One such example is the PharmFilm® technology, initially developed by the MonoSolRx/MonoSol and afterward advanced through partnership with Aquestive Therapeutics and other collaborators (Steinbach, 2018). Academic research into orodispersible films established the framework, but the clinical translation required collaboration with industry's regulatory strategy, production infrastructure, and market coverage. This mucoadhesive technology enabled the approvals of Suboxone® (buprenorphine hydrochloride and naloxone hydrochloride buccal film) and Sympazan® (clobazam oral film), which continue to be commercial leaders in their therapeutic categories (BioSpace, 2008, 2010).

Another successful example is the clinical collaboration between academia and industry. For example, Access Pharmaceuticals collaborated with many of the oncology centers affiliated with universities to conduct the clinical trials of MuGard™, a mucoadhesive oral rinse for the treatment of oral rinse. MuGard™ is a validated supportive therapy, evidenced by the published clinical trial in a peer-reviewed journal, that provided the base to adopt it by payers and clinicians (Allison et al., 2014). Ultimately, the performance record of the mucoadhesive products supports that industry-academic collaborations cannot be taken as optional, as they provide the strong base for the clinical translation of mucoadhesive platforms into broadly accessible therapies.

## 8 Challenges in clinical translation

### 8.1 Manufacturing scalability and process transfer

Manufacturing scalability and process validation of MDDS are considered after well-optimized studies. Industrial-scale production machines often help to determine the adaptability and reproducibility of the manufacturing process that is going to be conducted. Production at an industrial scale is difficult as compared to the lab-scale batch, as the different mucoadhesive dosage forms, like patches, films, and hydrogels, are produced by different unit operations (hot melt extrusion, solvent casting, and spray coating) and have their own risks (Maniruzzaman et al., 2017; Kong et al., 2015).

Scalability of the aforementioned approaches differs substantially. Solvent casting is a widely used technique for designing mucoadhesive films at the laboratory scale, but solvent evaporation and drying can become energy-intensive during scale-up. Additionally, mechanical attributes and film thickness can be affected due to drying parameter variations (Hassan et al., 2023). Hot-melt extrusion provides a continuous manufacturing route and avoids solvent evaporation, but the associated thermal and shear stress challenges may limit its suitability for thermolabile drugs or polymers (Shipp et al., 2022). For producing the fibrous patches, electrospinning is an attractive approach due to its tunable morphology and drug-release characteristics. However, industrial scalability incorporates multiple physicochemical and operational challenges. Conventional single-needle electrospinning presents a low production rate (0.01–2 g/h), thereby being unviable for commercial manufacturing (Omer et al., 2021). A multi-needle scale-up approach is introduced to overcome the low production rate hurdle; however, nozzle clogging and electrostatic field interference affect the efficiency. While needleless (free-surface) electrospinning successfully eliminates capillary clogging to achieve continuous high-throughput manufacturing, it requires substantially higher driving voltages to overcome polymer surface tension (Omer et al., 2021; Halim et al., 2025). These differences highlight that fabrication technology should be selected according to the drug's stability, polymer characteristics, required dosage-form properties, and intended manufacturing scale.

After getting approval from the FDA for marketing the particular product, any changes made in its formulation, such as changing the excipients, afterward require scaling up the formulation again. A guidance document is provided by the FDA for Scale-Up and Post-Approval Changes (SUPAC), a guidance document for industry for immediate release and modified release, i.e., SUPAC-IR/MR for solid oral dosage forms (Pawar et al., 2012).

Scaling up of MDDS often faces significant challenges. The quality of the polymer determines the mucoadhesion strength in the MDDS. So, variations in the polymer's processing conditions, its MW distribution, and its purity cause irregularities in the duration and mucoadhesive strength of the product (Lotfipour et al., 2021). Most of the time, the mucoadhesive properties are improved by incorporating blends of different polymers, but maintaining their constant ratio and distribution at a larger scale becomes challenging (Benitez-Martinez et al., 2023; Joyce et al., 2021), disturbing the overall mucoadhesive performance. Maintaining uniformity in surface chemistry, particularly after the functionalization/surface treatment, is often difficult across different batches (Abbas et al., 2025). Product specifications should define the critical parameters such as polymer composition, blend ratios, and mucoadhesive strength to achieve the optimal mucoadhesive strength (Kurra et al., 2016). These considerations align with a QbD framework, in which raw material characteristics and process parameters are controlled to confirm uniform product quality and mucoadhesive performance during scale-up (Patel et al., 2026).

### 8.2 Interpatient variability in mucosal environments

Variation in mucosal environment among patients creates the major barrier for the clinical translation of MDDS. Variability of the mucosal environment in terms of flow rate of saliva, enzymatic activity, local pH, mucosal thickness, and mucin composition can markedly influence mucoadhesion, dissolution, and swelling, which may ultimately affect drug bioavailability (Špiljak et al., 2025). For example, in xerostomic patients, films show reduced mucoadhesion due to reduced hydration, while gels are washed away before effective adhesion due to excessive salivation (Vinke et al., 2020). In patients with OM, proteolytic activity is increased due to inflamed mucosa, which may alter the mucus composition; as a result, the bonds between mucus and polymer become loose and local irritation is increased, causing the patient discomfort (Mariaule et al., 2021). Other factors, such as disease state and patient age, also influence the mucosal environment (Lynge Pedersen et al., 2015). Moreover, clinical adoption of the vaginal route for vaginal DDS is limited by several barriers, including mucus turnover, variations in vaginal fluid volume and pH, enzymatic degradation (Ahmed et al., 2026), menopause-associated changes in vaginal epithelial thickness (de Laat et al., 2023), and the complex dynamics of vaginal microflora (Joseph et al., 2021). In addition, the compatibility of mucoadhesive formulations with the vaginal microflora (Ahmed et al., 2026) and the selection of an appropriate mucoadhesive formulation technique may further influence drug retention and absorption (de Laat et al., 2023). Such interpatient variability may consequently contribute to differences in clinical endpoints, including residence time, local drug concentration, and treatment response, thereby complicating the interpretation of clinical trial outcomes (Hwang et al., 2026).

### 8.3 Long-term safety and patient acceptance

The mucoadhesive polymers and excipients used for MDDS should not only be productive for increasing the mucoadhesion of the drug on the mucosal surface but also have a good safety profile without causing target-site effects such as hypersensitivity, local irritation, and mucosal barrier modifications (Camargo, 2023). Although the safety studies are performed for the materials used in the formulation under the defined conditions for a specified duration, the time of mucoadhesion can differ and relies on various factors such as mucosal environment, material composition, and design of the formulation (Bassi da Silva et al., 2017; Abrantes et al., 2016). For MDDS, the modified and prolonged interactions of the mucoadhesive polymers with the mucosal surface create biocompatibility issues (Abbas et al., 2025). Degradation products of synthetic polymers can be cytotoxic, affecting mucosal surface integrity and causing damage to cellular components, particularly when exposed for longer durations and used in high concentrations (Cui et al., 2011). Slow-degrading and non-biodegradable polymers can remain longer in tissues, triggering inflammation and toxicity (Shastri, 2003). In addition, degradation of these polymers in acidic environments can change the mucosal pH and increase the infection risk (Zhang et al., 2024). The delayed adverse effects and continuous inflammatory response due to the turnover of the mucosal surface can negatively influence the long-term safety of these products. Extended tolerability and biocompatibility studies should be performed for the drugs and devices, respectively, to check the safety endpoints (Agency, 2025). Another important concern is the long-term safety of thiolated polymers intended for MDDS. The degree of thiolation, polymer structure, degradation products, and residual reagents may influence their safety profile (Yang et al., 2023). Therefore, these chemically modified polymers warrant additional safety characterization, including the assessment of their potential genotoxicity (Seabra et al., 2014). Although the previously reviewed literature showed good biocompatibility and low genotoxic effects of thiolated polymers, these findings are primarily based on short-term *in vitro* and acute *in vivo* assessments. Evidence regarding the long-term genotoxicity of thiolated polymers remains limited and requires further assessment to support their clinical translation (Noreen et al., 2024).

Patient acceptance for particular dosage forms depends on various factors such as administration schedule, ease of use, tolerability, irritation, patient comfort, and the taste-masking effect of the product, and it could be challenging for clinical translation of mucoadhesive products or devices if not monitored properly (Ahmed et al., 2024; Sharma et al., 2012). For example, special populations, i.e., the elderly and children, may often face problems swallowing the dosage form, which sticks to the wall of the esophagus, causing discomfort. In elderly patients, xerostomia, while in children, film flexibility and taste pose unique challenges (Rai et al., 2023). Moreover, mucoadhesive strength and duration are important key elements involved in the product's effectiveness. Weak mucoadhesive strength causes early detachment, while excessive mucoadhesion causes irritation and discomfort, which results in reduced therapeutic effectiveness and may cause the patient to show non-compliant behavior (Jovanović et al., 2021). These findings suggested that clinical efficacy cannot be separated from patient usability, as the real-world outcomes are strongly influenced by these factors.

### 8.4 Reproducibility of adhesion and drug release

Regulatory acceptance of mucoadhesive products depends on reproducible mucoadhesive performance and drug release, but their reproducibility is often hindered due to a lack of standardized methods (Gilhotra et al., 2013). Many of the mucoadhesive *in vitro* studies are performed, such as shear stress, tensile strength, and wash-off tests, but poor correlation and inter-laboratory variability with *in vivo* results limit their use as standardized assays (Baus et al., 2019; Müller et al., 2022). *In vitro-in vivo* correlations are generally weak because the adhesion tests in the laboratory fail to mimic the enzymatic activity, changing mucus environment, and clearance mechanisms observed *in vivo* (Müller et al., 2022). Recent studies on formulation further demonstrated how the material composition can be optimized to change the drug release behavior. Catechins-loaded PVA microneedles incorporating gelatin microspheres were developed in one of the studies conducted by Sabbagh et al. Different microsphere concentrations, i.e., 0%, 5%, 10%, and 15%, were applied to PVA microneedles to demonstrate how the different microsphere concentrations influence the swelling and drug release behavior. The formulation with 10% gelatin microspheres showed the highest catechin release, reaching 42.9% at 12 h and 84.4% at 24 h, while the PVA control exhibited significantly lower release. These findings indicated that modifying the material composition and structural characteristics of a delivery system can present a means of altering drug release kinetics. Such optimization at the formulation level is relevant to MDDS development, where the residence time, mucoadhesive strength, swelling behavior, and drug release must be balanced to attain reproducible clinical efficacy (Sabbagh et al., 2024).

Extensive *in vitro* studies remain critical, conducted under controlled conditions for assessing biocompatibility, degradation behavior, and cytotoxicity of polymers and other excipients (Müller et al., 2022). For example, accelerated degradation studies giving insights into the breakdown of polymers often misrepresent the real-time byproduct interactions and degradation through *in vivo* studies (Weiss et al., 2008). Moreover, effects of chronic exposure to the excipients may not be detected by the short-term cytotoxicity assays and require advanced techniques, i.e., long-term co-culture systems, real-time degradation monitoring, and repeated dosing studies, to minimize the adverse biological effects before the preclinical evaluation (Prior et al., 2024; Goers et al., 2014; Hong et al., 2022; Huang et al., 2020).

## 9 Emerging innovations and future trends

The future development of MDDS is moving from empirical formulation optimization to more predictive and patient-centered designs. Emerging strategies include smart or stimuli-responsive delivery systems, nanotechnology integration, artificial intelligence (AI)-assisted polymer design, microbiome-aware delivery, and also prioritizing sustainable materials like greener polymers and manufacturing in order to reduce environmental impact. Importantly, development of reproducible relationships between material properties, mucosal biology, patient adherence, and relevant outcomes is essential for translational progress.

Smart mucoadhesive systems help to merge mucoadhesive polymers with stimuli-responsive attributes and evolve passive adhesion into responsive and programmable podiums (Gonciarz et al., 2025). By establishing these smart mucoadhesives, they help to actively sense and react to exogenous (temperature, magnetic, and light) and endogenous (pH, enzymes, and glucose) stimuli, thereby achieving local bio-interactions, improved release behavior, and better residence time (de Lima et al., 2022).

Stimuli-responsive biopolymers like carbohydrate polymers (CS, cellulose, and alginate) exhibited the potential for responsive drug delivery (Madineh et al., 2025). This approach could help to improve local efficacy and lower systemic exposure; however, investigating detailed mucosal safety testing along with stimulus-response behavior under the dynamic and heterogeneous conditions of human mucosa requires adequate attention.

Moreover, the incorporation of nanotechnology, i.e., liposomes, SLNs, NLCs, dendrimers, polymeric NPs, and many more, widens the potential of MDDS by delivering protection to labile ingredients, desired particle size, controlled drug release pattern, and tuned mucoadhesive features. Additionally, this strategy is advantageous for treating diverse pathological conditions (Desai et al., 2023). Recent experimental outcomes magnify the significance of NPs-incorporated MDDS for drug targeting and improved bioavailability (Kim et al., 2023). However, increasing structural complexity also introduces characterization, batch-to-batch reproducibility, long-term safety, and scale-up challenges. Regardless of this, nanomaterial biosafety data and regulatory clarity are still substantial for future clinical considerations.

Tedious trial-and-error is traditionally associated with polymers and formulation development; Artificial intelligence (AI) and Machine Learning (ML) deliver an opportunity to overcome these labor-intensive trial-and-error cycles toward accelerated, target-driven discovery loops. Multi-scale fingerprinting schemes are utilized in modern polymer informatics ecosystems. Basically, AI describes polymer attributes such as individual atoms, repeating units, and whole polymer chains; subsequently, the ML models use this information to predict polymer-specific features, such as stability, solubility, strength, or biodegradability. Moreover, instead of relying on forward property prediction, the newer models can assist in inverse design. For instance, methodologies like Bayesian optimization, genetic algorithms, and generative models can facilitate proposing the specific molecular structure for acquiring several objectives at the same time. Additionally, by incorporating virtual forward synthesis, it's possible to evaluate whether the AI model-suggested polymer is realistic to design or not. Overall, this integrated AI-driven approach can shorten the path from polymer design to experimental validation, accelerating the development of high-performance and sustainable materials (Tran et al., 2024).

Incorporation of organ-on-chip and multi-organ-on-a-chip technologies within the design process of MDDS constitutes an innovative complementary approach in advancing the translational process (Mitchell et al., 2026). These Microphysiological Systems (MPS) are overcoming critical drawbacks associated with static *in vitro* experiments and animal studies that are usually unable to reproduce the physiological response in humans (Mitchell et al., 2026; Özkan et al., 2024). MPS technology offers a highly predictive human-relevant platform for the systematic evaluation of various advanced delivery approaches, including engineered NPs, extracellular vesicles, gene vectors, and hybrid biomaterials, by characterizing the kinetics of barrier penetration, organ-specific delivery, cellular uptake, and off-target toxicities under dynamic fluid flow. Moreover, the integration of MPS technology with computational Physiologically-Based Pharmacokinetic (PBPK) modeling allows quantitative prediction of the pharmacokinetic and bioavailability characteristics of MDDS formulations (Mitchell et al., 2026). Table 7 indicates the quantitative pharmacokinetic evidence of enhanced drug exposure following administration of MDDS. Development of microbiome-aware or microbiome-active DDS is another emerging strategy (Kamath et al., 2025). Microbial communities at mucosal surfaces, i.e., oral cavity, GIT, respiratory tract, and vagina, can influence drug stability, metabolism, local inflammation, and therapeutic response. Unlike conventional systems that merely coexist with or attempt to evade the microbiota, microbiome-active DDS treat the microbiome as an active design element, utilizing specific microbial cues to trigger spatiotemporally controlled drug activation, retention, or release (Wilson et al., 2017). This concept is particularly relevant to MDDS because prolonged residence at mucosal surfaces could facilitate interaction with localized microbial environments. However, the clinical translation of these platforms remains limited by significant inter-individual microbiome variability, unresolved regulatory pathways for hybrid biologic-material systems, and the complexities of achieving scalable, GMP-compliant manufacturing (Kamath et al., 2025; Dinesh et al., 2023).

**Table 7:** Quantitative pharmacokinetic evidence of enhanced drug exposure following administration of mucoadhesive drug delivery systems.

Drug	MDDS formulation	Route	Comparator	PK Analysis	C_max_ (MDDS vs control)	T_max_ (MDDS vs control)	AUC (MDDS vs control)	Bioavailability/Relative Bioavailability/AUC ratio	References
Rizatriptan	Mucoadhesive buccal film (Proloc/HPMC/Eudragit RS100)	Buccal	Oral solution	Non-compartmental	169.43 ± 28.67 ng/mL vs 83.85 ± 17.35 ng/mL	1.5 h (Both)	994.86 ± 95.79 ng h/mL vs 406.45 ± 61.08 (0–12 h)	Relative Bioavailability: 244.77%	Nair et al. (2021)
Dimenhydrinate	Mucoadhesive buccal film (HEC/Xanthan gum)	Buccal	Oral solution	Non-compartmental	1395 ± 136 ng/mL	NR	4644 ± 237 ng h/mL vs 1851 ± 86 ng h/mL (AUC_0_–_7_ _h_)	∼2-fold AUC	Yildiz et al. (2015)
Loratadine	Sodium-hyaluronate mucoadhesive nanosuspension	Intranasal	Oral/Intranasal reference formulation	Non-compartmental	Oral: 13.29 ± 5.716 nM vs 6.388 ± 2.205 nM; Intranasal: 39.991 ± 14.180 nM vs 38.357 ± 9.778 nM	NR	Oral: 36.588 ± 9.785 vs 17.812 ± 1.962; Intranasal: 202.708 ± 43.311 vs 110.353 ± 10.414 (AUC_0-∞_) [h nmol L^−1^]	Relative bioavailability: 5.54-fold for oral; 1.84-fold for intranasal	Alshweiat et al. (2020)
L-Dopa	CS-loaded polymeric NPs	Intranasal	L-Dopa solution	Non-compartmental	70.01 ± 5.77 μg/mL vs 50.018 ± 3.25 μg/mL	90 min vs 60 min	11054.16 ± 1153.50 vs 6494.582 ± 272.04	Absolute bioavailability: 45.20 ± 4.72% vs 26.56 ± 1.11%	Ahmad et al. (2022)
Fexofenadine	CS-coated liposomes	Intranasal	Oral powder formulation	Non-compartmental	220 ± 30 μg/mL vs 340 ± 290 μg/mL	0.5 h vs 0.9 ± 0.8 h	1040 ± 120 ng h/mL vs 920 ± 280 ng h/mL	Absolute bioavailability: 34.7 ± 6.3% vs 6.2 ± 2.0%	Qiang et al. (2012)
Bimatoprost	Bimatoprost 0.03% in DuraSite	Ocular	Conventional bimatoprost 0.03% solution	Non-compartmental	Aqueous humor (AH): 26.57 ± 19.16 ng/mL vs 12.11 ± 21.72 ng/mL and iris-ciliary body (ICB): 65.0 ± 26.20 ng/mL vs 10.73 ± 9.01 ng/mL	AH: 0.5 vs 1 h; ICB: 0.5 (Both)	AH: 24.29 vs 12.31; ICB: 95.14 vs 14.44 AUC0.25–24 h (ng/g·h or ng/mL·h)	AH: 2.0-fold AUC; ICB: 6.6-fold AUC	Shafiee et al. (2013a)
Sumatriptan	CS-coated liposomes (CCL)	Intranasal	Nasal sumatriptan solution	Non-compartmental	IV: 234 ng/mL; Solution: 6 ng/mL; Nanoliposomes: 17 ng/mL; CCL: 30 ng/mL	IV: 15 min; Solution: 90 min; Nanoliposomes: 90 min; CCL: 60 min	IV: 14860; Solution: 1051; Nanoliposomes: 3478; CCL: 5575 (AUC_0–240_ (ng.min/ml))	Not Reported	Assadpour et al. (2022)
Ondansetron	Mucoadhesive nasal microspheres (*Caesalpinia pulcherrima* galac*tomannan*)	Intranasal	Oral solution	Non-compartmental	34 ± 20.23 vs 29 ± 19.32 ng/mL	60 vs 15 min	43.04 ± 1.67 vs 23.98 ± 4.32 ng min/mL (0–∞)	58.65 ± 4.54% relative F vs 34.24 ± 2.89%	Suryawanshi et al. (2015)

Therapeutic Drug Monitoring (TDM) is mandatory for drug dosage optimization and reducing toxicity. However, the conventional methods for TDM are tedious; to conquer this challenge, wearable sensors have emerged as a promising alternative (Liu et al., 2023). For instance, physiological and biochemical signals can be monitored via smart wearable patch systems that merge biosensors and delivery systems for personalized healthcare. Consequently, drug carriers that respond to stimuli can initiate ingredient release on demand upon the action of actuators (Khadka et al., 2023). This technique could be integrated into MDDS to enable personalized dosing. Furthermore, in drug development and delivery, artificial intelligence and ML are transforming the pharmaceutical sector (Jena et al., 2024). These models can facilitate customizing the formulation to individual mucosal features and estimate residence time, supporting personalized treatment, improving effectiveness, and reducing unwanted side effects to potentially improve patient compliance and therapeutic outcomes. However, clinical validation data and regulatory approvals need consideration to explore the full therapeutic potential of these models.

Another parameter is sustainability that modifies the material selection for MDDS. Biodegradable and biocompatible polymers like CS, alginate, and hyaluronic acid stand out as sustainable and non-toxic materials (Chicea et al., 2024; Dubashynskaya et al., 2024). This aspect could help to overcome the regulatory challenges associated with synthetic polymer consumption. This could make the MDDS more confined to enhance its translational potential. Future development should consider not only polymer biodegradability but also solvent use, manufacturing energy requirements, formulation waste, packaging, and the lifecycle of increasingly complex delivery platforms. Importantly, sustainability should complement rather than substitute for rigorous assessment of safety, reproducibility, performance, and regulatory suitability.

## 10 Conclusion

The MDDS have evolved from simple bioadhesive formulations into sophisticated, clinically relevant platforms capable of addressing long-standing challenges in pharmacotherapy across multiple mucosal routes. Advances in polymer science, formulation engineering, and surface functionalization supported by nanotechnology where appropriate have enabled MDDS to enhance drug residence time, improve mucosal permeation, and achieve sustained and controlled release profiles. These attributes have translated into tangible pharmacological benefits, including improved bioavailability, reduced dosing frequency, and enhanced patient adherence, particularly for therapies requiring localized or non-invasive administration. The growing number of clinically evaluated and regulatory-approved mucoadhesive products across oromucosal, nasal, ocular, GI, vaginal, and rectal routes underscores the translational feasibility and therapeutic versatility of MDDS. Importantly, successful examples demonstrate that optimal performance arises not solely from advanced material design but from the careful integration of mechanistic understanding, patient-centric considerations, and robust formulation strategies. Conversely, the commercial failure or withdrawal of certain products highlights that clinical efficacy alone is insufficient without parallel attention to usability, long-term safety, manufacturing scalability, and regulatory compliance.

From a translational standpoint, this review highlights the critical role of QbD, excipient safety profiling, and early regulatory engagement in guiding MDDS development toward clinical and commercial success. The increasing complexity of advanced mucoadhesive systems, particularly those incorporating nanostructured components or functionalized polymers, necessitates rigorous evaluation of biocompatibility, reproducibility, and long-term performance under real-world conditions. In this context, strong academia-industry collaboration remains essential to bridge the gap between laboratory innovation and patient-ready products. Looking ahead, future progress in MDDS will likely be driven by rational polymer selection, precision functionalization strategies, and a deeper understanding of mucus biology and interpatient variability, complemented by AI-assisted formulation optimization and human-relevant models such as organ-on-chip platforms. Emerging opportunities include stimuli-responsive systems, personalized and disease-adapted formulations, microbiome-aware delivery systems, and hybrid platforms that may reduce treatment burden and support adherence. Realizing these opportunities will require their integration with standardized characterization, QbD principles, scalable manufacturing, rigorous safety and clinical validation, and early regulatory engagement. Collectively, these advances could provide a more evidence-based pathway for translating innovative MDDS from experimental platforms into safe, effective, and patient-acceptable therapies.

## Ethics declaration

Not applicable.

## Declaration of competing interest

The authors declare that they have no known competing financial interests or personal relationships that could have appeared to influence the work reported in this paper.

## Data Availability

Data will be made available on request.
